# The Spectrum of Singly Ionized Atomic Iodine (I ii)

**DOI:** 10.6028/jres.064A.045

**Published:** 1960-12-01

**Authors:** William C. Martin, Charles H. Corliss

## Abstract

The I ii spectrum has been excited in electrodeless lamps and photographed from 655 A to 11084 A. Wavelengths and estimated intensities are given for almost 2,400 lines. A revision and extension of the earlier analyses of this spectrum has increased the number of known even levels from 43 to 124, and the number of odd levels from 55 to 190. New *g_J_*-factors are given for 46 levels, and the previous designations of 40 levels are changed. Improved measurements in the vacuum ultraviolet region give a correction of 7.4 cm^−1^ to be subtracted from the values listed in *Atomic Energy Levels*, Vol. 3 (1958), for all levels above the ground configuration. The approximately 1,800 classified lines now include all of the strongest lines. The ^1^S_0_ of the ground configuration 5*s*^2^5*p*^4^ has been found, and this configuration has been fitted to intermediate coupling theory. Magnetic dipole transitions between levels of the ground configuration, ^3^P_2_–^1^D_2_ (7282 A) and ^3^P_1_–^1^S_0_ (4460 A), have been observed and their nature confirmed by the Zeeman effect. The line 5*p*^4 3^P_2_–^1^D_2_ shows hyperfine structure which is in approximate agreement with a theoretical calculation of the expected structure. New levels have been found for almost all higher configurations. All previously known series have been extended and new ones found. From one of the new series, $5p^{3}{(}^{4}S{}^{\circ})5-12g^{5}G_{6}^{{}^{\circ}}$, the principal ionization energy for I ii (154304 ±1 cm^−1^) has been derived. The results of the analysis are compared with theoretical expectations in a number of cases.

## 1. Introduction

One of the purposes served by the systematic compilation of *Atomic Energy Levels* [1] 1 being carried out at NBS is to point out inadequacies in the existing analyses of atomic spectra. The work reported here was partly stimulated by the need revealed under the scrutiny of this program for new observations and analyses of the iodine and bromine spectra. Another reason for our interest in these particular spectra is that iodine and bromine are the halogens most frequently used in the electrodeless metal-halide lamps [2] developed in this laboratory. These lamps have proved to be excellent sources of metallic spectra. We have also found that the electrodeless lamp gives a strong pure iodine spectrum when used as described below. Since the spectrum of the halogen contained in a metal-halide lamp appears along with that of the metal, the user of these lamps needs a complete and accurate knowledge of the iodine and bromine spectra. The present or recently completed work of this laboratory includes new descriptions and analyses of the first and second spectra of both iodine and bromine.

The spectrum of singly ionized iodine has previously been analyzed by P. Lacroute [3] and by K. Murakawa [4]. The results of their work, together with some preliminary revisions and extensions made possible by the new observations reported here, are given in *Atomic Energy Levels* (AEL), Vol. III. Our observations are superior to earlier measurements in being more complete (particularly in the vacuum ultraviolet region) and more accurate. Also important is our ability to distinguish better between the first and second spectrum.

## 2. Observations

The sources for the spectrum in the region 2000 to 11000 A were electrodeless lamps made from 5-mm i.d. quartz or vycor tubing about 10 cm long with a hemispherical window blown at one end and a side arm attached. These were thoroughly evacuated and outgassed. and a few crystals of iodine distilled into them before sealing off. The discharge was excited with the Raytheon Microtherm microwave generator which operates at 2,450 Mc with 125-w output. We made all observations with the lamp end-on except when Zeeman patterns were photographed. The spectra were dispersed with gratings having 30,000, 15,000, and 7,500 lines per inch, each mounted in parallel light to give stigmatic images. From 2000 to 2400 A the plate factor was 2.2 A/mm; from 2400 to 4400 A, 1.0 A/mm; from 4400 to 9000 A, 2.0 A/mm; from 9000 to 10400 A, 5 A/mm; and from 10400 to 11100 A, 10 A/mm.

Most of the wavelength values given in table 10.1, the line list (sec. 10, Appendix), are averages of measurements made on more than one plate. The intensities are visual estimates, meaningful only for lines in the same spectral region. Almost all of the stronger I ii lines show hyperfine structure under high resolution. Where the structures were completely or partially resolved, we measured the individual components. With few exceptions, however, only the weighted average of the component wavelengths is given in table 10.1. The intensity given for such a line is the sum of the estimated component intensities. Murakawa [4, 5] has observed interferometrically the hyperfine structure of a number of I ii lines.

Because of the line-broadening due to structure, the wavelength measurements here are not as accurate as one might wish. However, the disagreement between the observed wavenumbers and the corresponding differences of the “best” values for the relevant energy levels is usually less than 0.1 cm^−1^ for lines in the region above 2500 A.

When the iodine in the side arm of the discharge tube was kept at 30°C, corresponding to about 1-mm vapor pressure, most lines of the first spectrum appeared in the discharge, with only the strongest lines of I ii showing. As the temperature was lowered, the second spectrum increased in intensity until it became stronger than the first spectrum. The discharge ceased at about −28°C, when the pressure was between 10^−2^ and 10^−3^ mm Hg. We were able to assign most lines to the proper spectrum by including on every spectrogram these four spectra in juxtaposition: iron arc, “high” temperature (20 to 30° C) iodine spectrum, “low” temperature (≈−25°C) iodine spectrum, and iron arc.

This procedure leaves the origin of some of the weaker lines doubtful for two reasons: the weaker I i lines appear in the low pressure discharge but not at the higher pressure, while the I iii spectrum is also faintly excited at low pressures. The latter observation was surprising, but a comparison of the intensities in our list with those given by observers [3, 6, 7] of rather high energy discharges in iodine vapor leaves no doubt that our low pressure source gives the strongest I iii lines weakly. However, we believe that by this comparison we have eliminated the I iii lines from the list. Those unclassified lines above 5000 A which have intensity less than 50 comprise practically all the remaining lines of doubtful origin. These lines may belong to either the first or second spectrum.

We have observed the Zeeman effect in a field of about 37000 gauss for most of the strong I ii lines in the region 4000 to 9000 A. The technique is described by Kiess and Corliss [8]. Zeeman patterns obtained from these observations are given in table 10.2.

The spectrum was dispersed in the region 584 to 2000 A by a 2-m radius concave grating of 30,000 lines per inch ruled directly on pyrex. This grating, which was ruled under the direction of R. W. Wood at Johns Hopkins, was originally mounted in a vacuum spectrograph by K. T. Compton and J. C. Boyce [9]. W. R. Bozman has designed a new housing for the grating and plateholder of the old instrument. It consists primarily of a cylindrical section of steel pipe 75 cm in diameter and 210 cm long fitted with aluminum end covers which are sealed to the main tube by means of O-ring flanges. The slit is contained in a side tube. The optical arrangement is essentially that of Compton and Boyce except that the angle of incidence has been decreased slightly to about 13.1° to give the normal spectrum at 1920 A. The plate factor is 4.26 A/mm at the normal and the plateholder covers the range 0 to 2570 A in the first order. The height of the plateholder is adjustable externally so that several different exposures may be made on one plate.

We mounted a glass discharge tube end-on to the slit housing by means of an O-ring seal. After a side tube was charged with iodine, the lamp was evacuated and excited as described above. The slit of the evacuated spectrograph was open directly to the light from the discharge. We used Eastman SWR plates below 1400 A and Ilford Q–2 plates in the region above 1400 A. The first order spectrum was measured from 500 to 2500 A; and from 800 to 1200 A all but the weaker lines were measured in the second order. Most lines were measured three or four times. Calculated wavelengths of lines in the I i spectrum [8] served as the principal standards in the region 1200 to 2060 A. Lines of helium, oxygen, nitrogen, and carbon also were used for standards. Some bands, due principally to carbon monoxide, appeared on the spectrograms.

Lines of bromine were found on all our vacuum region plates. The existing measurements of the bromine spectra were not complete enough for the elimination of these lines from our list. We have excited, photographed, and measured the Br I and Br ii spectra in the vacuum ultraviolet. Analyses of these spectra are underway in this laboratory and will be reported later.

We have obtained averaged values of the I ii 5*p*^4^ ground-configuration energy levels relative to the lower odd levels by using the measurements of lines from 1100 to 1300 A. These odd levels are known relative to each other with good accuracy from measurements above 2000 A. We then calculated the wavelengths of all singly-classified lines observed in the vacuum ultraviolet. Since these wavelengths are on a consistent scale and the I ii spectrum is fairly rich below the I i limit (1200 A), we have thought it worthwhile to give these calculated wavelengths in table 10.1. Together with the I i wavelengths above 1200 A they comprise a consistent set of wavelengths over a large part of the vacuum region and should prove useful in reducing low- and medium-dispersion plates obtained with metal iodide lamp sources. An estimated uncertainty in the calculated I ii wavelengths of ±0.005 A at 1100 A gives ±0.002 A at 665 A, where the first calculated lines occur. The new I ii wavelengths in the vacuum ultraviolet region yield, for the energy levels above those of the ground configuration, values which are 7.4 cm^−1^ less than the corresponding values given in AEL.

## 3. Ground Configuration

**Figure 1:**
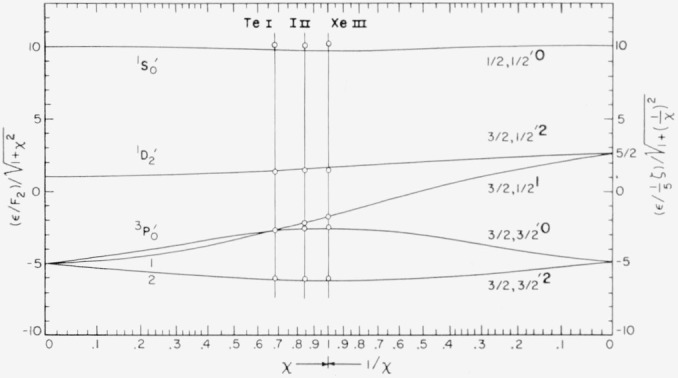
The levels of 5*p*^4^ for three atoms fitted to theoretical intermediate-coupling curves.

The 5*s*^2^ 5*p*^4^ ground configuration of singly ionized iodine gives the Russell-Saunders terms ^3^P, ^1^D, and ^1^S, as shown in table 1. The energy levels for these terms are listed in table 10.3, which includes all presently known even levels for I ii. Lacroute’s values for ^3^P_1_, ^3^P_0_, and ^1^D_2_ are about 3 cm^−1^ too high relative to the ground state ^3^P_2_. The level he designated ^1^S_0_ is not real. In the three atoms for which the configuration 5*p*^4^ is known (Te i, I ii, and Xe iii) one finds good agreement with intermediate coupling theory. Robinson and Shortley [10] have calculated the coupling parameters for 5*p*^4^ in these atoms by making ^3^P_1_, ^3^P_2_, and ^1^D–^3^P*_c_* exactly fit the theoretical curves given in Condon and Shortley [11], p. 301, and reproduced in figure 1. This method gives a good fit for all levels except ^1^S_0_ which lies too high in all three cases. (The position for I ii 5*p*^4 1^S_0_ in figure 1 of reference [10] must be corrected since only the incorrect value of Lacroute was available to Robinson and Shortley—this reduces the disagreement to about one third of its former value.) As Robinson and Shortley point out, this does not mean that ^1^S_0_ is perturbed—an interaction would push the term *down.* We have calculated the parameters by the method Condon and Shortley originally used for Te i, i.e., by making ^3^P_1_, the mean of ^1^D_2_ and ^3^P_2_, and the mean of ^1^S_0_ and ^3^P_0_ fit exactly. This brings ^1^S_0_ down to excellent agreement while leaving the value of *χ*=⅕*ζ _p_*/F_2_ (see Condon and Shortley for notation) essentially unchanged in all cases, but the agreement of the J = 2 levels is not as good as in Robinson and Shortley. As a compromise we have averaged the values of the parameters obtained by these two methods. The results are given in table 2 and the corresponding values of the observed energy levels are plotted in figure 1, again making ^3^P_1_ fit exactly. Since the Landé, Slater, and coupling ratios all are functions only of the parameter *χ* the good agreement obtained for them by Robinson and Shortley is retained here. The new ^1^S term makes the agreement of the observed Slater ratio (^1^S–^1^D)/(^1^D–^3^P*_c_*) in I ii (1.481) with the theoretical value (1.365 for *χ* = 0.844) about the same as in Te i and better than in Xe iii.

The eigenvectors of the two second-order energy matrices for *p*^4^ in intermediate coupling, for J=0 and J=2, respectively, are functions of *χ* only. By calculating these eigenvectors and squaring the elements of each, one obtains the percentage composition of each level of a given J in terms of the pure Russell-Saunders levels of that J. The results of this calculation for *χ*=0.844 (I ii 5*p*^4^) are that ^1^D_2_ and ^3^P_2_ have 90.3 percent Russell-Saunders purity (i.e., they are 9.7% mixed), while the purity for ^1^S_0_ and ^3^P_0_ is 83.5 percent. Hence the *LS* designations assigned these levels are not without some meaning.

## 4. Magnetic Dipole Transitions

**Figure 2:**
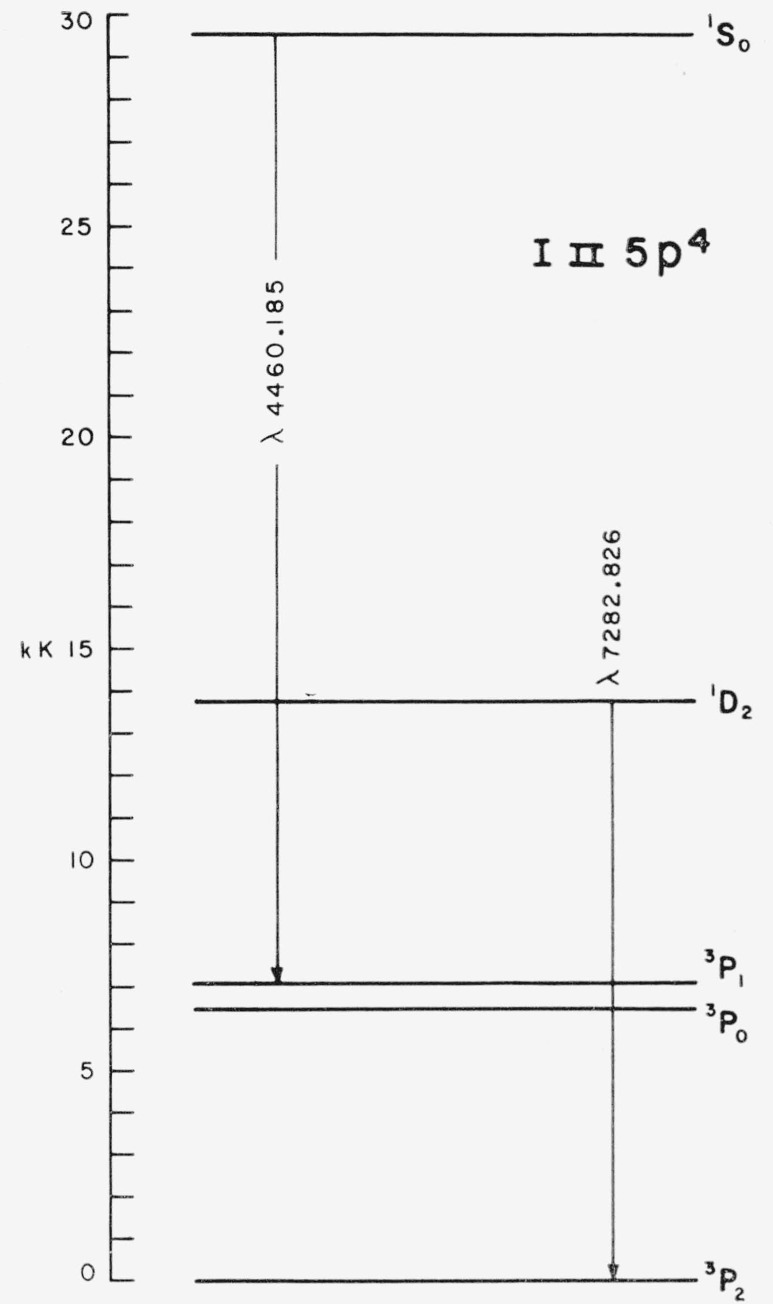
Two magnetic dipole transitions observed in I ii.

“Forbidden” transitions between pairs of levels belonging to the *p*^4^ configuration have been observed in laboratory sources of the spectra Oi (the aurora line at 5577 A, ^1^S_0_–^1^D_2_), Se i [12], Te i [13], Xe iii [14], and Po i [15]. We have found two such transitions in I ii: 5*p*^4 3^P_1_–^1^S_0_ (4460.185 A) and 5*p*^4 3^P_2_–^1^D_2_ (7282.83 A). A diagram of the ground configuration levels showing these transitions is given in figure 2. The observed wavenumber of each line is that predicted by the separation of the relevant levels as determined from lines in the vacuum ultraviolet. The transition probabilities as derived from magnetic dipole strengths tabulated against *χ* by Shortley et al. [16] are 99 sec^−1^ for the line at 4460 A, and 9.1 sec^−1^ for the line 7282 A (*χ* = 0.844; both lines have zero transition probability in pure Russell- Saunders coupling, where *χ*=0). Of course the observed intensities in table 10.1 have no absolute meaning, and for these two lines in different spectral regions do not give even relative experimental intensities. Both transitions appear much stronger in the high pressure (I i) discharge than with low pressure (I ii) excitation. This first spectrum character is not surprising since these transitions occur between levels which are series limits for the I i spectrum. The levels lie about 100,000 cm^−1^ above the I i ground state, while the excitation energy for any I ii line in this region is almost twice that amount. In his note on the xenon spectra, Edlén [14] mentioned that such lines were at first experimentally referred to the next lower ionization than that to which they actually belonged.

**Figure 3:**
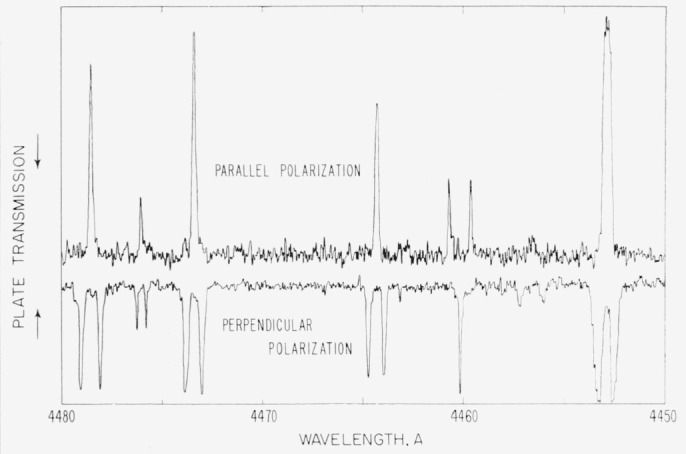
Microphotometer traces of an iodine spectrogram showing the transverse Zeeman effect in the region near 4460 A. The “upside-down” Lorentz triplet observed for the I ii line at 4460.185 A is definite proof of its magnetic-dipole character.

The line at 4460 A is pure magnetic dipole radiation, and its classification was made certain by the transverse Zeeman effect observation shown in figure 3. After the line had been classified, a search through the Zeeman data of Kiess and Corliss yielded this pattern. In a field of about 37,000 gauss the line splits into a triplet with separation of 1.51 Lorentz units (L.U.). But the unshifted perpendicularly polarized component and the symmetrically displaced parallel components form a pattern that is the inverse of the other triplets appearing in the figure, and can arise only from a magnetic dipole transition. The derived *g_J_*-value for 5*p*^4 3^P_1_ is, of course, 1.51. By observing the Zeeman effect for the analogous transition in Pb i, 6*p*^2 3^P_1_–^1^S_0_at 4618A, H. Niewodniczanski [17] gave the first definite experimental proof for the occurrence of magnetic dipole radiation in atomic spectra.

**Figure 4:**
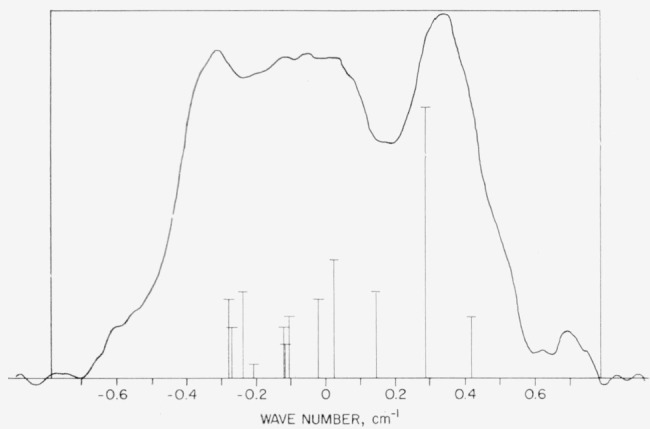
Microphotometer trace showing the hyperfine structure of the forbidden line 5*p*^4 3^*P*_2_–^1^*D*_2_ at 7282 A. The theoretical relative intensities and calculated positions of the components of this pattern are also indicated. The position of the trace with respect to the zero of the wavenumber scale is arbitrary.

The resolution obtained in the first order spectrum with a grating of 30,000 lines per inch was sufficient to resolve partly the hyperfine structure of the line 5*p*^4 3^P_2_–^1^D_2_ (7282 A), as shown in figure 4. There seem to be three or four components on our plates, though only one is well resolved from the rest. In order to compare the observed structure of this line with theory, we have calculated the hyperfine splittings of the 5*p*^4 3^P_2_ and 5*p*^4 1^D_2_ levels in 1^127^ ii. The required constants were taken as follows: nuclear spin=5/2, nuclear magnetic moment=2.81 n.m., nuclear electric quadrupole moment = −0.62× 10^−24^ cm^2^ [18]. The formulas of Goudsmit [19] and Casimir [20] give the magnetic splitting factors, A(J), and the electric quadrupole factors, B(J), respectively. Trees [21] has published convenient general expressions for evaluating these formulas. We have also taken the effects of intermediate coupling into account. The calculated splittings are A(^3^P_2_) = 0.033 cm^−1^, B(^3^P_2_) = −0.00017 cm^−1^, A(^1^D_2_) = 0.078 cm^−1^, B(^1^D_2_) = 0.00042 cm^−1^. If it is assumed that 5*p*^4 3^P_2_–^1^D_2_ is predominately a magnetic dipole transition, the same intensity ratios as for electric dipole radiation should apply to the hyperfine components [22, 23]. The “theoretical” hyperfine pattern in figure 4 is based on this assumption and the calculated splittings. In view of the approximations made in the theory, the agreement of the calculated and observed patterns seems satisfactory and may be regarded as a confirmation of the suggested origin of this line.

**Figure 5:**
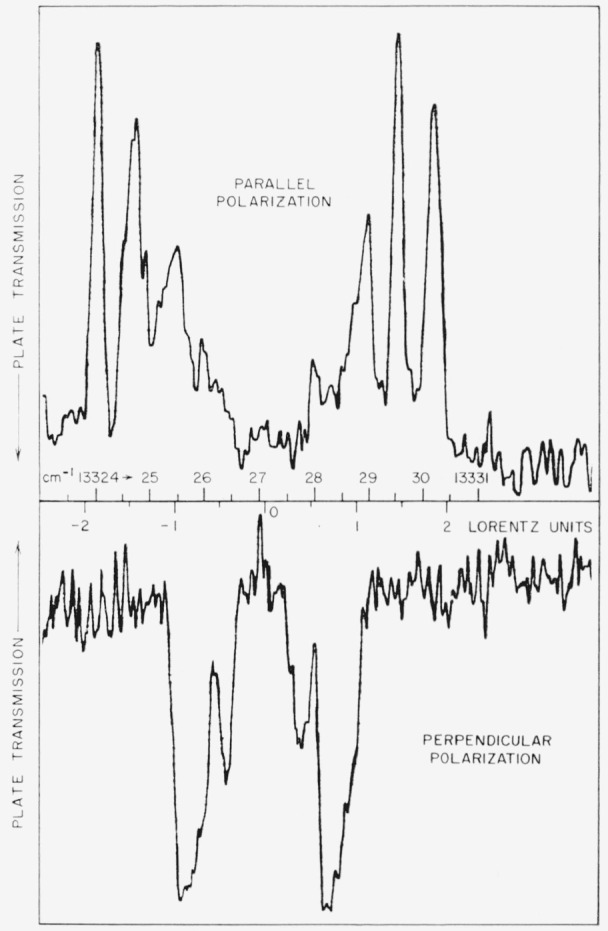
Microphotometer traces of the transverse Zeeman pattern for the line at 7282 A. The spectrogram from which these traces have been made was obtained with the source in a field of 35,500 gauss. In order to locate the Zeeman components with respect to the undisplaced hyperfine pattern, an exposure with the source in zero magnetic field was also made on this spectrogram (not shown). The zero of the above Lorentz unit scale is taken at the same position, relative to the observed hyperfine pattern of figure 4, as the zero of the wavenumber scale in figure 4.

Since the Zeeman effect for a forbidden line showing hyperfine structure has rarely been observed, three separate several-hour exposures were made especially to obtain the magnetic splitting of the line 7282 A in a field of 35,500 gauss. A microphotometer tracing of one of the Zeeman patterns obtained is shown in figure 5. Assuming the calculated intermediate coupling *g_J_*-values of 1.451 for 5*p*^4 3^P_2_ and 1.049 for 5*p*^4 1^D_2_, one obtains the calculated Zeeman-component positions given in table 3. The observed values, which are averages for measurements of the three patterns, are also given. The agreement of calculated and observed positions and the reversal of the respective polarizations from those expected for an electric dipole transition are definite proof of the origin of this line. The average values of the *g_J_*-factors derived from the measured patterns are 1.457 for ^3^P_2_ and 1.046 for ^1^D_2_.

A broadening due to unresolved hyperfine structure is apparent for most of the components in figure 5. Since the splitting of either level by the external magnetic field is here several times larger than the zero-field hyperfine splitting, the formula for the Back-Goudsmit case [24] should give a fair approximation for the total energy of each magnetic- hyperfine state. This formula predicts a splitting of each Zeeman component into 2I+1 = 6 equally spaced hyperfine components. The expected overall width of a given Zeeman component due to this structure can be obtained from the previously calculated values of the hyperfine splitting factors for the two levels (see above) and the standard formula for the Back-Goudsmit effect [24]. These calculated widths are given in table 3. Neglecting for a moment the observed asymmetries, one can see that the general features of the pattern are in accord with these widths. In particular, what might at first be taken as large deviations from the predicted relative intensities of table 3 can probably for the most part be explained as effects due to the different widths. For example, the appearance of the distinct and relatively narrow *π*-components at ±1.85 L.U., as contrasted with the barely observable structures at ±0.65 L.U.—where components with the same total intensities as those at ±1.85 L.U. should occur—is, no doubt, due principally to the fact that the predicted overall width of the innermost components is 10 times that of the outermost components. Since the additional broadening of all components due to Doppler and instrumental effects has not been considered, the calculated widths of table 3 should be taken only as indicative. Previous experience with uncooled electrodeless lamps excited as described above indicates effective gas temperatures of 4,000 to 8,000 °C when the lamp is in a strong magnetic field. At these temperatures the Doppler width of each transition contributing to one of the Zeeman components of 7282 A is comparable to the “total width” of table 3 for the narrowest components (0.06 cm^−1^).

The asymmetries, with respect to total width and peak intensity, observed between members of the symmetrically positioned pairs of components in figure 5 are confirmed on the other two Zeeman spectrograms of this pattern. The complete Back- Goudsmit effect would divide each Zeeman component into six equally intense components without altering the overall symmetry of the usual Zeeman pattern. It seems likely that the asymmetries are caused more by deviations from the intensity rule for the Back-Goudsmit case than by the remaining asymmetries in the level positions. With the present resolution, no significant asymmetries in the positions of the centers-of-gravity of corresponding components are observed.

The line ^3^P_2_–^1^D_2_ is allowed in electric quadrupole radiation as well as in magnetic dipole radiation. Although it is certain that 7282 A is almost pure magnetic dipole in character, it might be possible to deduce from a symmetric Zeeman pattern a slight admixture of electric quadrupole radiation in the line. This was done for a similar transition, 6*p*^2 3^P_1_–^1^D_2_ in Pb i, by Jenkins and Mrozowski [25]. These investigators also were able to confirm an interesting “interference” effect [23] between the magnetic dipole and electric quadrupole radiation in this line. An application of this type of analysis to the pattern in figure 5 does not appear feasible because of the asymmetries due to hyperfine structure. It is possible that a detailed calculation of this pattern by use of the formulas for the “intermediate field” case would be worthwhile.

## 5. Higher Even Terms

The higher terms of I ii are of three types, according to whether the parent term in I iii is 5*p*^3 4^S°, ^2^D°, or ^2^P°. As shown in table 1, these are denoted by affixing to the valence electron no prime, one prime, or two primes, respectively. No meaningful analysis exists for I iii, but one can estimate the positions of 5*p*^3 2^D and 5*p*^3 2^P from their positions in Sb i and Te ii. The result is that the ^2^D_1½,2½_ levels should lie about 12,000 and 15,000 cm^−1^, respectively, above ^4^S, and the ^2^P_½,1½_ at approximately 25,000 and 30,000 cm^−1^, respectively.

We have indicated in our discussion of 5*p*^4^ the extent to which Russell-Saunders designations are meaningful for that configuration. For many of the higher levels such designations have considerably less meaning. The overlapping of the “terms” belonging to a given configuration and parentage signifies the usual heavy-atom departure from Russell-Saunders coupling, and makes the L-S naming of some of these higher levels little more than a convenient accounting system. With the reader thus warned we shall, however, use this notation.

Earlier investigators found the levels of the even groups (^4^S°)6*p*, (^2^D°)6*p*′, and (^4^S°)4*f.* Transitions from these levels to lower odd levels are responsible for the strongest I ii lines in the air region. The intensities of the 5*d*–4*f* transitions indicate that the strong line at 2993.866 A is due to 5*d* ${\;}^{3}D_{1}^{{}^{\circ}}-4f$ ^3^F_2_. This requires that the designations of the levels previously called 5*d* ${\;}^{3}D_{1}^{{}^{\circ}}$ and 6*s*′ ${\;}^{3}D_{1}^{{}^{\circ}}$ be interchanged, as well as those of 4*f* ^3^F_2_ and 6*p*″ ^3^D_2_. Murakawa [4] noted that the ${\;}^{3}D_{1}^{{}^{\circ}}$ levels were almost indistinguishable, and Lacroute [3] mentioned “l’analogie” of the J=2 levels involved. Neither of these authors had available a complete line list with a uniform intensity scale. We unfortunately did not notice Murakawa’s incorrect designation of the 4*f* levels as 5*f*—an error retained by Lacroute—until after the publication of AEL, Vol. III. Our measurement of these strong multiplets has resulted in some designation changes. While some configuration mixing undoubtedly takes place for each of these pairs, the intensities of most of the other combinations of these levels confirm our changes. In the next section it will be shown that the present choice of ${\;}^{3}D_{1}^{{}^{\circ}}$ is supported by theory. The relative position of the level here designated 4*f* ^3^F_2_ is supported by the position of ^3^F_2_ in the newly-found 5*f* configuration. Also on the basis of our intensities and *g*-values, we have reversed Murakawa’s designations of the pairs 6*p*′ ^3^F_3_, ^3^D_3_ and 6*p*′ ^1^P_1_, ^3^D_1_.

The 7*p* ^5,3^P levels, all of which have been found, overlap the 4*f* levels but do not make as strong combinations. The level 7*p* ^5^P_2_ was previously known.

The terms of (^2^P°)6*p*″ are now complete. Lacroute gave five of these levels correctly, although the level he designated 6*p*″ *y*_1_ has a J-value of 2. As noted above, his 6*p*″ ^3^D_2_ fits better in the 4*f* configuration. We assign his 6*p*″ ^3^P_2_ to 6*p*″ ^1^D_2_. The 6*p*″ group overlaps both the 4*f* and 7*p* levels. From the line intensities it appears that the 4*f* levels and 7*p* ^5^P_3_ are relatively pure, while most of the remaining levels of 7*p* and 6*p*″ are configuration-mixed.

The analysis has yielded all but two of the levels arising from 4*f*′ and 7*p*′. Since these groups overlap and mix, any individual assignment to configuration and Russell-Saunders designation at best represents only the principal contribution to a complete description of a given level. Lacroute found five of these levels, assigning them to 7*p*′. We have assigned all but one of these to 4*f*′. Four other levels listed by Lacroute for 7*p*′ are not real.

All the levels of the complete 5*f* configuration are new except ^3^F_3_, which was given by Lacroute as 7*p*′ ^4^P_1_. Most of the levels of *nf* are now known through *n*=8. Four levels are assigned to 9*f*—three of them tentatively—on the basis of series regularity.

The level given in table 10.3 as 8*p* ^3^P_2_ was erroneously assigned a J-value of 3 in the AEL table, while 8*p* ^3^P_1_ was given by Lacroute as 7*p*′ ^3^D_2_. The 8*p* levels fall amongst those of 4*f*′ and 7*p*′, but the present designations seem most likely on the basis of combination intensities and series considerations.

A group of 13 higher even levels which may belong to any of four configurations, as shown in table 10.3, have been given only numerical designations.

## 6. Odd-Parity Configurations

Table 10.4 is a complete list of the presently known odd levels in I ii. The terms of 5*s*5*p*^5^, 5*p*^3^ (^4^S°)6s, (^4^S°)5*d*, and (^2^D°)6*s*′ were previously known. The 5*p*^5^, 6*s*, and 5*d* configurations are probably mixed, but the new intensities leave no doubt that Lacroute’s designations for 5*p*^5^ ${\;}^{3}P_{0}^{{}^{\circ}}$ and ${\;}^{5}D_{0}^{{}^{\circ}}$ must be reversed. The resulting new position of ${\;}^{5}D_{0}^{{}^{\circ}}$ is confirmed in the 6*d* configuration.

**Figure 6:**
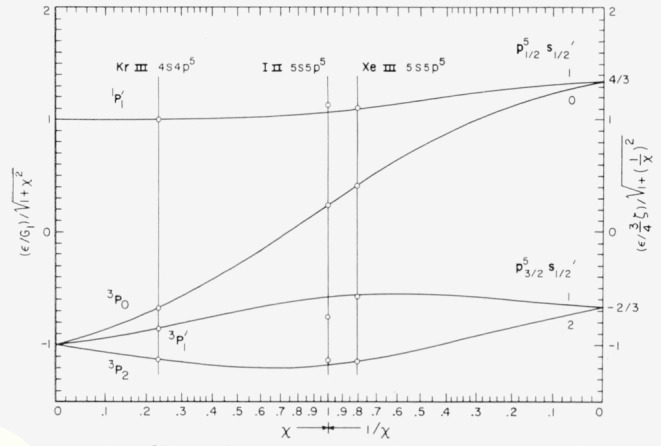
Observed levels of *sp*^5^ for three atoms fitted to intermediate-coupling curves.

A comparison of the predictions of intermediate coupling theory for *p*^5^*s* with the experimental results for a number of rare-gas type atoms is made in the book of Condon and Shortley ([11], p. 304). It seems of interest to make this comparison for the known examples of *sp*^5^ which exhibit intermediate coupling to a significant extent. There are only three of these, and the results for them are shown in table 4 and figure 6. The levels for Kr iii 4*s* 4*p*^5^ and Xe iii 5*s* 5*p*^5^ were fitted to the intermediate coupling formulas in Condon and Shortley by the method used there: the parameter *χ* is chosen so that the levels J=0,2, and the mean of the two levels of J=1, fit the theory exactly. The value of *χ* chosen for I ii 5*s* 5*p*^5^ gives a somewhat better fit than is obtained by the Condon and Shortley method. Still, the fit for I ii is not as good as for the other two atoms. Interaction with either 5*d* ${\;}^{3}D_{1}^{{}^{\circ}}$ or 6*s*′ ${\;}^{3}D_{1}^{{}^{\circ}}$, or with both, probably causes the apparent depression of 5*p*^5^ ${\;}^{3}P_{1}^{{}^{\circ}}$ in I ii. In any case this calculation definitely supports the designation change for 5*d* ${\;}^{5}D_{0}^{{}^{\circ}}$ and 5*p*^5^ ${\;}^{3}P_{0}^{{}^{\circ}}$ mentioned above. The other known cases of *sp*^5^, in Cl ii through Sc vi of the S i isoelectronic sequence, are quite close to L-S coupling (*χ*<0.1).

The level adopted here for 5*p*^5^ ${\;}^{3}P_{0}^{{}^{\circ}}$ in Xe iii is listed as 5*d* ${\;}^{5}D_{0}^{{}^{\circ}}$ in AEL, Vol. III. The analogy with I ii and Kr iii leaves little doubt that our change should be made. The three combinations [26] on which the level is based were not sufficient to determine its configuration unambiguously. Humphreys’ [26] level for 5*p*^5^ ${\;}^{3}P_{0}^{{}^{\circ}}$ in Xe iii would seem questionable; it is far lower than the expected position of 5*d* ${\;}^{5}D_{0}^{{}^{\circ}}$, the only other possible designation. The known levels 5*d* ${\;}^{5}D_{3,2,1}^{{}^{\circ}}$ of Xe iii agree with the order found in I ii.

A diagonalization of the energy matrix in intermediate coupling for the configuration 5*p*^3^ 6*s* in I ii has increased our confidence in the configuration and parentage assignments made here for all the known odd levels based on 5*p*^3 2^D° and 5*p*^3 2^P° parents. The matrices of the electrostatic and spin-orbit interactions for *sp*^3^ are given on pages 199 and 268, respectively, of Condon and Shortley [11]. The value of F_2_(5*p*,5*p*) used in our calculation, 1425 cm^−1^, was obtained from the relation $6F_{2}{=}^{3}P_{0}^{{}^{\circ}}{-}^{3}D_{3}^{{}^{\circ}}$ for *sp*^3^. It is supported by the value of F_2_ for the 5*p*^4^ configuration given above, 1405 cm^−1^. If one knows F_2_ and the levels of either J=2 or J=1, the value of G_1_(6*s*,5*p*) may be obtained from the diagonal-sum rule. At the time the value of G_1_ adopted here (1000 cm^−1^) was chosen, however, it was based on values obtained from the equation 5*p*^3^(^4^S°)6*s* ${(}^{3}S_{1}^{{}^{\circ}}{-}^{5}S_{2}^{{}^{\circ}})=4G_{1}$. This gives G_1_ = 952 cm^−1^ for I ii and G_1_=1035 cm^−1^ for Xe iii. By carrying out the calculation for G_1_ = 500, 1000, and 1500 cm^−1^ we have assured ourselves that the intermediate value is very close to the “best” value for fitting the observed levels. The other parameter needed for the calculation, *ζ_p_*, was taken to be 5930 cm^−1^, as in the 5*p*^4^ configuration.

The calculated energies and *g_J_*-values for the levels of 5*p*^3^6*s* are given in table 5, together with the observed values. An exact fit is assumed for the ${\;}^{3}D_{0}^{{}^{\circ}}$ and ${\;}^{3}P_{0}^{{}^{\circ}}$ levels. The average deviation of the predicted and observed values, 322 cm^−1^, seems reasonable for a calculation neglecting configuration mixing. Table 6 gives the calculated composition of the levels of J=1 and J=2 in terms of pure Russel-Saunders states.

It will be seen that the aforementioned designation of the level at 94825 cm^−1^ as 6*s*′ ${\;}^{3}D_{1}^{{}^{\circ}}$, instead of the level at 92133 cm^−1^, is supported by this calculation. The indicated perturbation of 6*s*′ ${\;}^{3}D_{1}^{{}^{\circ}}$ is in the direction to be expected if it is caused mainly by interaction with 5*d* ${\;}^{3}D_{1}^{{}^{\circ}}$. The other ambiguities resolved by this calculation were between the 5*d*′ and 5*d*″ levels on the one hand and those belonging to 6*s*″ on the other. As between the 5*d*′ and 6*s*″ groups, the present assignments are in general supported by the relative intensities of combinations of levels belonging to these configurations with levels of the distinct 6*p*′ and 6*p*″ groups.

The intensities would not have served, however, to distinguish the 6*s*″ levels from those of 5*d*″. The assignments of the two levels 5*d*″ ${\;}^{1}D_{2}^{{}^{\circ}}$ and 5*d*″ ${\;}^{3}D_{3}^{{}^{\circ}}$ may be considered questionable, since the former is well below a level of J=2 assigned to 5*d*′ and the latter is near the level designated 5*d*′ ${\;}^{1}F_{3}^{{}^{\circ}}$. However, these assignements fit in with the other levels of J=2 and J=3 within each parentage group better than any other arrangement. The 5*d*′ group is now known except for one level of J=5, and only ${\;}^{3}F_{4}^{{}^{\circ}}$ is lacking in the 5*d*″ group.

The sorting-out of these lower odd levels, together with an approximate knowledge of the various series limit positions which they approach, have enabled us to assign definite configuration and parentage designations to the complex group of 91 odd levels between 118074 cm^−1^ (5*d*′ ${\;}^{3}P_{1}^{{}^{\circ}}$) and 145837 cm^−1^ $\left(20_{2,1}^{{}^{\circ}}\right)$. Only eight of the levels expected in this region are missing, four of them from 9*d* ^5,3^D°. The levels of 6*d* ^5,3^D° and 7*s* ^5,3^S° were previously known and correctly identified. About 15 other previously listed higher odd levels are included in this analysis, usually with a changed designation or a new assignment to a definite configuration.

It is interesting to note that the smallness of the ${\;}^{3}D_{1}^{{}^{\circ}}{-}^{3}D_{2}^{{}^{\circ}}$ intervals in the 7*s*′ and 8*s*′ terms (2.37 and 36.12 cm^−1^, respectively) is in accord with the theoretical predictions for the corresponding levels of 6*s*′. In table 5 the predicted positions of 6*s*′ ${\;}^{3}D_{1}^{{}^{\circ}}$ and ${\;}^{3}D_{2}^{{}^{\circ}}$ are both very near 94100 cm^−1^. The large observed separation of these levels, 1134 cm^−1^, is apparently due to perturbations in opposite directions for the two levels.

We have assigned 15 levels to the 6*d*′ group, but there is really no good basis for giving them term designations; hence they are listed numerically. The Russell-Saunders designations given the 5*d*′ and 5*d*″ levels probably should not be taken as more than suggestive, but the names given here fit the observed combinations better than any other arrangement.

**Figure 7:**
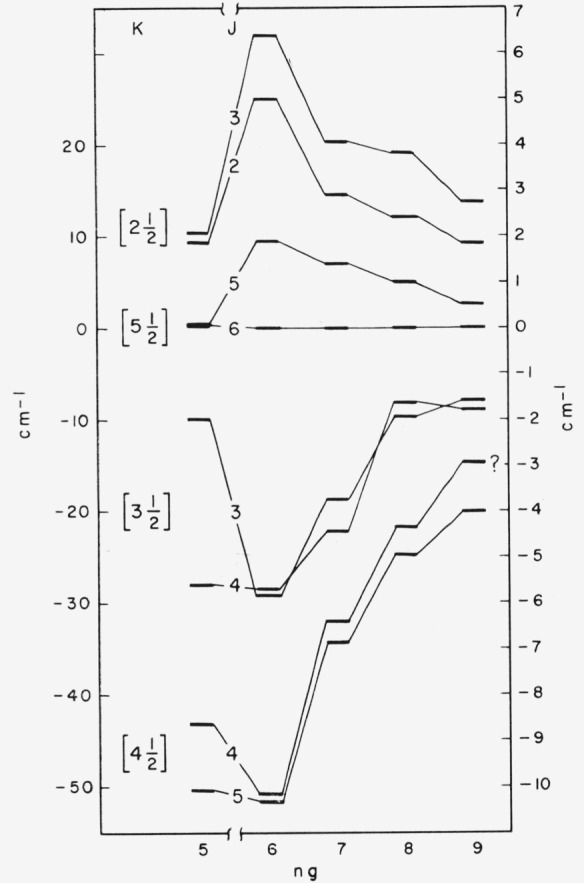
Levels of the (^4^*S*°) *ng* groups. The ordinate scale on the left applies to the 5*g* levels, the higher levels being plotted according to the scale on the right. The Russell-Saunders designations for these levels may be obtained by comparing this figure with the level values given in table 10.4.

The levels of the newly-found (^4^S°)*ng* ^5,3^G° terms in table 10.4 are based on combinations with 4*f* ^5,3^F and, in the case of the higher series members, with 5*f* ^5,3^F. Although we have assigned multiplicities to the *ng* levels on the basis of these transitions—intercombinations are definitely weaker than intrasystem transitions—it is clear from figure 7 that these levels do not fall into the triplet-quintet pattern of Russell- Saunders coupling. The numbers under “K” in this figure refer to pair-coupling [27]; they are the four possible results of adding the orbital angular momentum of a *g*-electron to the J of the parent ${\;}^{4}S_{1\frac{1}{2}}^{{}^{\circ}}$ level. While these levels certainly do not show good pair-coupling, it seems probable that this notation describes the observed structures better than the L-S scheme in table 10.4.

Each *ng* triplet level is paired with a quintet level in the three higher “pairs” of figure 7. In the known I ii terms of the type (^4^S°)*nl* ^5^L, ^3^L, where *l*= *p, d, f*, or *g*, a characteristic partial inversion of levels occurs except in *np* ^5^P. (Both of the known terms 6*p*, 7*p*, ^5^P show large deviations from the Landé interval rule in the direction of partial inversion.) However, this deviation from Russell-Saunders structure is compounded by overlapping of the triplet and quintent terms only in the (^4^S°)*ng* groups. Even the (^4^S***°***)*nf* ^3^F terms are well above the corresponding quintets.

It would seem from figure 7 that some of the *ng* levels are perturbed by amounts comparable to the (small) level separations. The position of the 5*g* ${\left[5\frac{1}{2}\right]}_{5}$ level and the crossing-over behavior of the ${\left[3\frac{1}{2}\right]}_{3,4}$ pair with each increase in *n* are probably the most obvious examples. Perhaps the best overall description of the grouping observed for these *ng* levels would be “frustrated pair-coupling.”

In addition to the identified series members based on the 5*p*^3^(^4^S°) parent, the higher group of odd levels beginning with $20_{1,2}^{{}^{\circ}}$ at 145837.7 cm^−1^ probably contains levels belonging to each of the designations 6*d*″, 7*d*′, 7*s*″, 8*s*′, and 5*g*′. We have given only numerical designations to these levels, no attempt having been made to assign them to definite parents and configurations.

## 7. Series and the Ionization Energy

In a singly-ionized atom, the Rydberg denominator n* for a series member is defined by the equation 4T= R/(n*)^2^; where 4T is the position of the level measured from the series limit, and R is the Rydberg constant. (The quantity T is the “reduced” absolute term value. For convenience in using the Rydberg Interpolation Table [28], the value of R was taken as 109737.4 cm^−1^ in the series calculations reported here.) Many unperturbed series obey quite closely the Ritz formula n*=n+μ+αT, where *n* runs through the successive integers for the series and *μ* and *a* are constants for a given series. Since the fraction by which n* exceeds an integer is a linear function of T for such a series, the position of the series limit is taken as that value which gives the “best” straight line when these successive fractions are plotted against the corresponding values of T.

**Figure 8:**
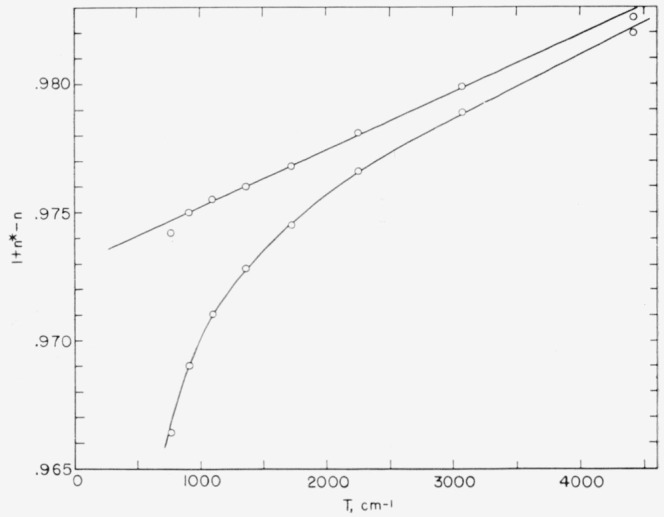
The (^4^*S*°) *ng* ^5^*G*°_6_ series plotted for two different limit values, 154304 cm^−1^ (straight line) and 154308 cm^−1^ (curved line). The series runs from n=5, at the right, through n=12.

Figure 8 shows two such plots for the new (^4^S*°*)*ng* ${\;}^{5}G_{6}^{{}^{\circ}}$ series, *n=5* through 12. The approximately straight line is obtained by taking the 5*p*^3 4^S° limit, the ground state of I iii, to lie 154304 cm^−1^ above the 5*p*^4 3^P_2_ ground level of I ii. The curved line, representing a very unlikely behavior for this series, results from assuming the limit to be only 4 cm^−1^ above the straight-line value. It is seen that the Rydberg denominator fractions for the higher series members become increasingly sensitive to the limit chosen or, equivalently, to the series level values as *n* increases. The last point on the left in the straightline plot, for *n*=12, would fall on the straight line if the position of 12*g* ${\;}^{5}G_{6}^{{}^{\circ}}$ were raised by 0.2 cm^−1^. Since this level is based on two weak lines—transitions to 4*f* ^5^F_5_ and 5*f* ^5^F_5_–the observational uncertainty in its position is about 0.1 cm^−1^. In any case a series of this regularity leaves small doubt about the reality of any of its members.

**Figure 9:**
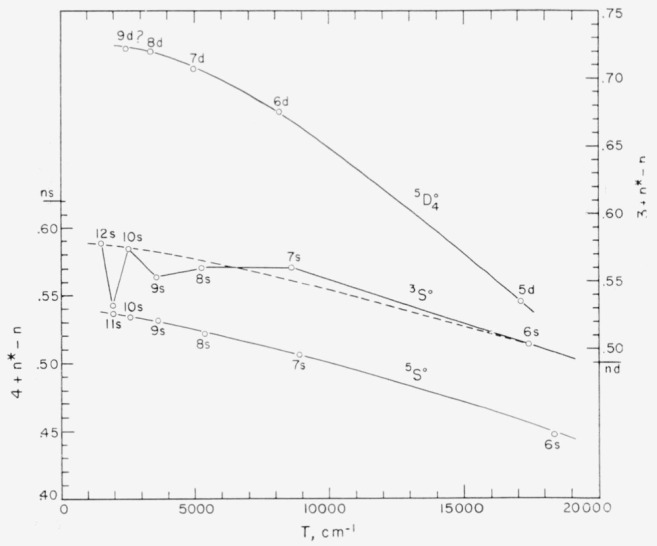
Three series in I ii based on the I iii 5p^3 4^S° limit at 154304 cm^−1^ above the ground level of I ii.

Another example of a fairly regular series, *ns* ^5^S°, is shown in figure 9, along with the perturbed *ns* ^3^S° series. The behavior of the latter series is certainly too irregular to be caused by a single perturbing level. By analogy with the ^5^S° series, one might guess that the dashed line shown in figure 9 approximates a hypothesized “unperturbed” behavior of the ^3^S° series. The fact that 9*s* ${\;}^{3}S_{1}^{{}^{\circ}}$ combines strongly with 5*p*^4^ ^1^D_2_, whereas the analogous transition for the other *ns* ^3^S° levels does not occur or is quite weak, suggests that the level 6*d*′ $15_{1}^{{}^{\circ}}$ is mainly responsible for the depression of 9*s* ${\;}^{3}S_{1}^{{}^{\circ}}$. This $15_{1}^{{}^{\circ}}$ level, only 375 cm^−1^ above 9*s* ${\;}^{3}S_{1}^{{}^{\circ}}$, combines with 5*p*^4 1^D_2_ in a transition just twice as strong as the 5*p*^4 1^D_2_–9*s* ${\;}^{3}S_{1}^{{}^{\circ}}$ line. Similarly, an interaction probably exists between 11*s* ${\;}^{3}S_{1}^{{}^{\circ}}$ and either or both of the slightly higher levels $25_{1}^{{}^{\circ}}$ and $29_{1}^{{}^{\circ}}$. The perturbations of the ^3^S° series are actually not very large. For example, the perturbation required to remove the point for 11*s* ^3^S° from the dashed line to the position shown is only 90 cm^−1^ (a change of 22 cm^−1^ in the reduced term value).

The *nd* ${\;}^{5}D_{4}^{{}^{\circ}}$ series is shown in figure 9 as an example of an apparently regular series which nevertheless fails to obey the simple Ritz formula. It is clear that a limit position determined by assuming such a series to be Ritzian will be too low. A previous value reported [29] by us for the principal ionization energy of I ii, 19.12 ev, corresponds to a limt of 154260 cm^−1^ derived from the *ns* ${\;}^{5}S_{2}^{{}^{\circ}}$ and *nd* ${\;}^{5}D_{4}^{{}^{\circ}}$ series.

On the basis of the *ng* ${\;}^{5}G_{6}^{{}^{\circ}}$ series in figure 8, it seems reasonable to take the position of I iii 5*p*^3 4^S° as 154304 ±1 cm^−1^ above the ground level of I ii. By combining this result with a recent value [30] for the conversion factor, (wavenumber)/(energy in electron-volts) =8066.03 ±0.14 cm^−1^ ev^−1^, we obtain 19.1301 ±0.0004 ev for the principal ionization energy of I ii. If the conversion factor adopted in the AEL volumes is applied to our new limit, the resulting energy is 19.126 ev.

## 8. Conclusion

The earlier analyses of the I ii spectrum, which yielded 43 even and 55 odd levels, have been revised and extended to include 124 even and 190 odd levels. The revisions include changed designations for over 40 levels, improved values for all previously known levels, and new Landé *g*_J_ factors for 46 levels. All previously known series have been extended and new ones found. The ionization potential of I ii is well determined by one of the new series, (^4^S°)*ng* ${\;}^{5}G_{6}^{{}^{\circ}}$. Wherever feasible, the results of the analysis have been compared with theory and with experimental findings for homologous and isoelectronic atoms.

This improved analysis was made possible by the observation of almost 2400 I ii lines excited in an electrodeless lamp. We have measured the Zeeman patterns of 83 lines. About 1800 lines are now classified, as compared to approximately 500 lines classified in previous analyses. None of the remaining unclassified lines is very strong. The present list includes approximately 300 lines in the vacuum ultraviolet region, 655 to 2000 A, and extends to 11085 A in the infrared. The measurement of the vacuum ultraviolet spectrum has yielded an accurate value for the connection between the higher levels and those of the ground configuration.

We have observed two magnetic dipole transitions among levels of the 5*p*^4^ ground configuration and verified their nature by the Zeeman effect.

## Figures and Tables

**Table 1:** Predicted terms of *I ii*

Configuration	Predicted terms				
	
5*s*^2^ 5*p*^4^ 5*s* 5*p*^5^	^3^P _^3^P°_ ^1^D _^1^P°_ ^1^S				
	
	${\;}_{n\geq 6}^{ns}$	${\;}_{n\geq 6}^{np}$	${\;}_{n\geq 5}^{nd}$	${\;}_{n\geq 4}^{nf}$	${\;}_{n\geq 5}^{ng}$
					
5*s*^2^ 5*p*^3^(^4^S°)*nx*	$\{{\;}_{{\;}^{3}S^{{}^{\circ}}}^{{\;}^{5}S^{{}^{\circ}}}$	^5^P	^5^D°	^5^F	^5^G°
		^3^P	^3^D°	^3^F	^3^G°
5*s*^2^ 5*p*^3^(^2^D°)*nx*′	$\{{\;}_{{\;}^{1}D^{{}^{\circ}}}^{{\;}^{3}D^{{}^{\circ}}}$	^3^(PDF)	^3^(SPDFG)°	^3^(PDFGH)	^3^(DFGHI)°
		^1^(PDF)	^1^(SPDFG)°	^1^(PDFGH)	^1^(DFGHI)°
5*s*^2^ 5*p*^3^(^2^P°)*nx*″	$\{{\;}_{{\;}^{1}P^{{}^{\circ}}}^{{\;}^{3}P^{{}^{\circ}}}$	^3^(SPD)	^3^(PDF)°	^3^(DFG)	^3^(FGH)
		^1^(SPD)	^1^(PDF)°	^1^(DFG)	^1^(FGH)

**Table 2:** Intermediate-coupling parameters and predicted level positions for the 5*p^4^* configuration in *Te i, I ii*, and *Xe iii*

	Te i	I ii	Xe iii
			
*χ*=⅕*ζ _p_/*F_2_	0.684 *cm*^−1^	0.844 *cm*^−1^	1.004 *cm*^−1^
*ζ*(5*p*)	4090	5930	8180
F_2_(5*p*,5*p*)	1195	1405	1630
			
${\;}^{3}P_{2}\{{\;}_{\text{obs}.}^{\text{pred}.}$	−171	−220	−480
	0	0	0
${\;}^{3}P_{1}\{{\;}_{\text{obs}.}^{\text{pred}.}$	(4751)	(7087)	(9795)
	4751	7087	9795
${\;}^{3}P_{0}\{{\;}_{\text{obs}.}^{\text{pred}.}$	4697	6325	7878
	4707	6448	8131
${\;}^{1}D_{2}\{{\;}_{\text{obs}.}^{\text{pred}.}$	10708	13929	17580
	10559	13727	17100
${\;}^{1}S_{0}\{{\;}_{\text{obs}.}^{\text{pred}.}$	22731	28924	36162
	23199	29501	37398

**Table 3:** Zeeman effect for the forbidden line at 7282 A The observed component positions are average values from 3 patterns obtained with a magnetic field of about 35,500 gauss. Only half the pattern is given since the centers of gravity of any two corresponding components were, within the errors of observation, located symmetrically with respect to the zero of the magnetic displacement scale in figure 5.

π-components $M_{J}{(}^{1}D_{2})\to M_{J}^{\prime }{(}^{3}P_{2})$	Theoretical relative intensity	Calculated position	Observed position	Calculated total width
				
2→1	2	0.647 L.U.	…………………	0. 615 cm^−1^
1→0	3	1.049	1. 042 L.U.	.390
0→1	3	1.451	1. 459	.165
1→2	2	1.853	1.860	.060
				
*σ*-components				
1→1	2	0.402 L.U.	0.393 L.U.	0.225 cm^−1^
2→2	8	.804	.808	.450

**Table 4:** Intermediate-coupling parameters and predicted level positions for the configuration *sp^5^* in three atoms

	Kr iii 4*s*4*p*^5^	I ii 5*s*5*p*^5^	Xe iii 5*s*5*p*^5^
			
χ=¾*ζ _p_*/G_1_	0. 236	1.000	1.240
*ζ*(*np*)	3741 cm^−1^	5900 cm^−1^	9621 cm^−1^
G_1_(*ns,np*)	11890	4425	5819
			
${\;}^{3}P_{2}^{{}^{\circ}}\{{\;}_{\text{obs}.}^{\text{pred}.}$	(115932) cm^−1^	81555 cm^−1^	(98263) cm^−1^
	115932	81908	98263
${\;}^{3}P_{1}^{{}^{\circ}}\{{\;}_{\text{obs}.}^{\text{pred}.}$	119358	85296	103686
	119381	84222	103569
${\;}^{3}P_{0}^{{}^{\circ}}\{{\;}_{\text{obs}.}^{\text{pred}.}$	(121544)	(90405)	(112694)
	121544	90405	112694
${\;}^{1}P_{1}^{{}^{\circ}}\{{\;}_{\text{obs}.}^{\text{pred}.}$	141899	95514	118909
	141876	95956	119026

**Table 5:** Predictions of intermediate-coupling calculation for *I ii 5p^3^ 6s* compared with observation The parameters were taken as follows: *ζ* (5*p*) = 5930 cm^−1^, F_2_(5*p*,5*p*) =1425 cm^−1^ and G_1_(6s,5p) = 1000 cm^−1^.

Designation	Predicted position	Observed position	Difference	Predicted *g_J_*	Observed *g_J_*
					
6*s* ${\;}^{5}S_{2}^{{}^{\circ}}$	81176 cm^−1^	81033 cm^−1^	143 cm^−1^	1.954	1.95
6*s* ${\;}^{3}S_{1}^{{}^{\circ}}$	84862	84843	19	1.877	1.753
6*s*′ ${\;}^{3}D_{1}^{{}^{\circ}}$	94096	94825	−729	0.703	0.653
6*s*′ ${\;}^{3}D_{2}^{{}^{\circ}}$	94103	93691	412	1.235	1.16
6*s*′ ${\;}^{3}D_{3}^{{}^{\circ}}$	(96651)	96651	0	1.333	1.36
6*s*′ ${\;}^{1}D_{2}^{{}^{\circ}}$	98035	97701	334	1.048	0.996
6*s*″ ${\;}^{3}P_{0}^{{}^{\circ}}$	(105201)	105201	0	……………	……………
6*s*″ ${\;}^{3}P_{1}^{{}^{\circ}}$	105749	106103	−354	1.383	……………
6*s*″ ${\;}^{3}P_{2}^{{}^{\circ}}$	110015	110459	−444	1.430	1.36
6*s*″ ${\;}^{3}P_{1}^{{}^{\circ}}$	111173	111962	−789	1.037	……………

**Table 6:** Calculated percentage compositions for the levels of *I ii 5p^3^ 6s* The composition of each level is given in the column under its assigned designation.

	6*s* ${\;}^{5}S_{2}^{{}^{\circ}}$	6*s*′ ${\;}^{3}D_{2}^{{}^{\circ}}$	6*s*′ ${\;}^{1}D_{2}^{{}^{\circ}}$	6*s*″ ${\;}^{3}P_{2}^{{}^{\circ}}$
				
	%	%	*%*	%
6*s* ${\;}^{5}S_{2}^{{}^{\circ}}$	91.7	4.5	0.3	3.6
6*s*′ ${\;}^{3}D_{2}^{{}^{\circ}}$	0.8	65.6	22.5	11.1
6*s*′ ${\;}^{1}D_{2}^{{}^{\circ}}$	0.4	13.7	75.6	10.2
6*s*″ ${\;}^{3}P_{2}^{{}^{\circ}}$	7.1	16.2	1.6	75.1

	6*s* ${\;}^{3}S_{1}^{{}^{\circ}}$	6*s*′ ${\;}^{3}D_{1}^{{}^{\circ}}$	6*s*″ ${\;}^{3}P_{1}^{{}^{\circ}}$	6*s*″ ${\;}^{1}P_{1}^{{}^{\circ}}$
				
	%	%	%	%
6*s* ${\;}^{3}S_{1}^{{}^{\circ}}$	87.4	7.9	0.2	4.6
6*s*′ ${\;}^{3}D_{1}^{{}^{\circ}}$	3.0	80.1	0.8	16.0
6*s*″ ${\;}^{3}P_{1}^{{}^{\circ}}$	3.6	4.9	77.1	14.4
6*s*″ ${\;}^{1}P_{1}^{{}^{\circ}}$	6.0	7.1	21.9	65.0

## Footnotes

1 Figures in brackets indicate the literature references on page 452.

## 10. Appendix

**Table 10.1:** Observed lines of *I* ii

λ(vac) observed	λ(vac) calculated	Intensity	Observed wavenumber, cm^−1^	Classification
655.804		2	152484.6	
657.029		1	152200.3	5*p*^4 3^P_2_— $51_{1,}^{{}^{\circ}}\;{\;}_{2,\;3}$
659.004		6	151744.1	5*p*^4 3^P_2_— $49_{1,}^{{}^{\circ}}\;{\;}_{2}$
661.412		1	151191.7	5*p*^4 3^P_2_–14*d* ${\;}^{3}D_{3}^{{}^{\circ}}$
663.977		8	150607.6	5*p*^4 3^P_2_–13*d* ${\;}^{3}D_{3}^{{}^{\circ}}$
664.031		3	150595.4	5*p*^4 3^P_2_— $48_{1,}^{{}^{\circ}}\;{\;}_{2}$
664.520	.512	8	150484.6	5*p*^4 3^P_2_— $47_{1,}^{{}^{\circ}}\;{\;}_{2}$
665.055	.041	8	150363.5	5*p*^4 3^P_2_— $46_{2}^{{}^{\circ}}$
665.135	·.132	5	150345.4	5*p*^4 3^P_2_— $45_{1,}^{{}^{\circ}}\;{\;}_{2}$
665.699	.695	150	150218.0	5*p*^4 3^P_2_— $43_{1,}^{{}^{\circ}}\;{\;}_{2}$
666.660	.653	150	150001.5	5*p*^4 3^P_2_— $42_{2}^{{}^{\circ}}$
667.270		100	149864.4	5*p*^4 3^P_2_— $41_{1,\;2,\;3}^{{}^{\circ}}$
668.264	.250	15	149641.4	5*p*^4 3^P_2_–12*d* ${\;}^{3}D_{3}^{{}^{\circ}}$
668.976	.981	25	149482.2	5*p*^4 3^P_2_— $40_{3}^{{}^{\circ}}$
672.505	.491	40	148697.8	5*p*^4 3^P_2_–11*d* ${\;}^{3}D_{3}^{{}^{\circ}}$
674.063		4	148354.1	5*p*^4 3^P_2_–12*s* ${\;}^{3}S_{1}^{{}^{\circ}}$
675.748	.732	1	147984.2	5*p*^4 3^P_2_— $34_{2}^{{}^{\circ}}$
676.493	.480	80	147821.2	5*p*^4 3^P_2_— $33_{3}^{{}^{\circ}}$
678.856	.853	200	147306.6	5*p*^4 3^P_2_—10*d* ${\;}^{3}D_{3}^{{}^{\circ}}$
679.155	.152	5	147241.8	5*p*^4 3^P_2_— $30_{2}^{{}^{\circ}}$
679.618	.616	50	147141.5	5*p*^4 3^P_2_—10*d* ${\;}^{3}D_{2}^{{}^{\circ}}$
680.971	.967	15	146849.2	5*p*^4 3^P_2_— $26_{3}^{{}^{\circ}}$
681.266	.269	6	146785.5	5*p*^4 3^P_2_— $25_{1,\;2}^{{}^{\circ}}$
682.180	.179	25	146588.9	5*p*^4 3^P_2_—11*s* ${\;}^{3}S_{1}^{{}^{\circ}}$
682.685	.682	40	146480.4	5*p*^4 3^P_2_— $24_{1,\;2}^{{}^{\circ}}$
683.414		1	146324.2	
683.569	.565	80	146291.0	5*p*^4 3^P_2_— $21_{1}^{{}^{\circ}}$
685.685	.694	1	145839.6	5*p*^4 3^P_2_— $20_{1,\;2}^{{}^{\circ}}$
689.294		15	145076.0	
689.817	.822	250	144966.0	5*p*^4 3^P_2_—9*d* ${\;}^{3}D_{3}^{{}^{\circ}}$
690.082		5	144910.3	
690.272		3	144870.4	
690.453	.455	80	144832.4	5*p*^4 3^P_2_—9*d* ${\;}^{3}D_{2}^{{}^{\circ}}$
691.194	.193	15	144677.2	5*p*^4 3^P_2_— 8*s*′ ${\;}^{3}D_{2}^{{}^{\circ}}$
691.295		5	144656.0	5*p*^4 3^P_1_— $49_{1,\;2}^{{}^{\circ}}$
693.567	.566	40	144182.2	5*p*^4 3^P_2_—10*s* ${\;}^{3}S_{1}^{{}^{\circ}}$
696.630		1	143548.2	
696.840		1	143505.0	5*p*^4 3^P_1_— $48_{1,\;2}^{{}^{\circ}}$
697.358	.353	1*h*	143398.4	5*p*^4 3^P_1_— $47_{1,\;2}^{{}^{\circ}}$
697.606		10	143347.4	
697.940	.936	5	143278.8	5*p*^4 3^P_1_— $46_{2}^{{}^{\circ}}$
698.032	.036	3	1.43259.9	5*p*^4 3^P_1_— $45_{1,\;2}^{{}^{\circ}}$
698.650	.656	8	143133.2	5*p*^4 3^P_1_— $43_{1,\;2}^{{}^{\circ}}$
699.697	.712	1	142919.0	5*p*^4 3^P_1_— $42_{2}^{{}^{\circ}}$
701.007		1	142651.9	
701.909		40	142468.6	
706.055	.044	400	141632.0	5*p*^4 3^P_2_— 8*d* ${\;}^{3}D_{3}^{{}^{\circ}}$
706.319		10	141579.1	
706.622	.611	3	141518.4	5*p*^4 3^P_2_— 8*d* ${\;}^{3}D_{1}^{{}^{\circ}}$
706.901	.898	150	141462.5	5*p*^4 3^P_2_— 8*d* ${\;}^{3}D_{2}^{{}^{\circ}}$
707.136	.133	100	141415.5	5*p*^4 3^P_0_— $33.5_{1}^{{}^{\circ}}$
708.122	.113	7	141218.6	5*p*^4 3^P_1_— $36_{2}^{{}^{\circ}}$
709.733	.720	50*w*	140898.0	5*p*^4 3^P_1_— $34_{2}^{{}^{\circ}}$
709.856	.871	1	140873.6	5*p*^4 3^P_2_— 8*d* ${\;}^{5}D_{2}^{{}^{\circ}}$
711.746	.742	6	140499.6	5*p*^4 3^P_2_— 6*d*′ $15_{1}^{{}^{\circ}}$
712.918	.914	200	140268.6	5*p*^4 3^P_1_— $31_{0}^{{}^{\circ}}$
713.152		2	140222.6	
713.502	.493	25	140153.8	5*p*^4 3^P_1_— $30_{2}^{{}^{\circ}}$
713.557	.565	10	140143.0	5*p*^4 3^P_0_—11*s* ${\;}^{3}S_{1}^{{}^{\circ}}$
713.654	·.645	1.00	14012:3.9	5*p*^4 3^P_2_— 9*s* ${\;}^{3}S_{1}^{{}^{\circ}}$
715.832	.830	150	139697.6	5*p*^4 3^P_1_— $25_{1,}^{{}^{\circ}}{\;}_{2}$
716.161	·.157	10	139633.4	5*p*^4 3^P_2_— 6*d*′ $14_{3}^{{}^{\circ}}$
716.583	.580	150	139551.2	5*p*^4 3^P_2_— 6*d*′ $13_{1,}^{{}^{\circ}}{\;}_{2}$
717.388	.390	80	139394.6	5*p*^4 3^P_1_— $24_{1,}^{{}^{\circ}}{\;}_{2}$
718.360	.36.5	1	139206.0	5*p*^4 3^P_1_— $21_{1,}^{{}^{\circ}}{\;}_{2}$
719.546	.539	1000	138976.5	5*p*^4 3^P_2_— 6*d*′ $12_{1,}^{{}^{\circ}}{\;}_{2}$
722.169		15	138471.7	5*p*^4 1^D_2_— $51_{1,}^{{}^{\circ}}{\;}_{2,3}$
722.980	.980	1000	1:38316.4	5*p*^4 3^P_2_— 6*d*′ $10_{2}^{{}^{\circ}}$
724.550		2	138016.7	5*p*^4 1^D_2_— $49_{1,}^{{}^{\circ}}{\;}_{2}$
725.432		10	137848.9	
725.976	.979	4	137745.6	5*p*^4 3^P_1_— 9*d* ${\;}^{3}D_{2}^{{}^{\circ}}$
726.019	.035	2	1377:37.4	5*p*^4 3^P_0_—10*s* ${\;}^{3}S_{1}^{{}^{\circ}}$
726.798	.794	3	137589.8	5*p*^4 3^P_1_— 8*s*′ ${\;}^{3}D_{2}^{{}^{\circ}}$
726.987	.985	10	137554.0	5*p*^4 3^P_1_— 8*s*′ ${\;}^{3}D_{1}^{{}^{\circ}}$
727.246	.246	800	137505.1	5*p*^4 3^P_2_— 6*d*′ $9_{3}^{{}^{\circ}}$
730.42:3		1	136907.0	
731.005	.002	7	136798.0	5*p*^4 3^P_0_— 7*s*″ ${\;}^{3}P_{1}^{{}^{\circ}}$
731.169		5	136767.3	
731.216	.213	7	136758.5	5*p*^4 1^D_2_— $47_{1,}^{{}^{\circ}}{\;}_{2}$
731.860	.853	20	136638.2	5*p*^4 1^D_2_— $46_{2}^{{}^{\circ}}$
731.961	.964	25	136619.3	5*p*^4 1^D_2_— $45_{1,}^{{}^{\circ}}{\;}_{2}$
732.092	.081	50	136594.9	5*p*^4 1^D_2_— $44_{2,3}^{{}^{\circ}}$
732.276	.269	15	136560.5	5*p*^4 3^P_2_— 6*d*′ $7_{1,}^{{}^{\circ}}{\;}_{2}$
732.910	.905	5	136442.4	5*p*^4 3^P_2_— 6*d*′ $6_{1,}^{{}^{\circ}}{\;}_{2}$
734.430	.433	1	136160.0	5*p*^4 3^P_1_— 7*s*″ ${\;}^{3}P_{1}^{{}^{\circ}}$
735.402	.401	2	1:35980.0	5*p*^4 3^P_1_— 7*s*″ ${\;}^{3}P_{0}^{{}^{\circ}}$
737.546	.545	500	135584.8	5*p*^4 3^P_2_— 7*d* ${\;}^{3}D_{3}^{{}^{\circ}}$
740.342	.343	25	135072.7	5*p*^4 3^P_0_— 8*d* ${\;}^{3}D_{1}^{{}^{\circ}}$
740.950	.959	300	134961.9	5*p*^4 3^P_2_— 7*d* ${\;}^{3}D_{2}^{{}^{\circ}}$
741.131	.129	6	134928.9	5*p*^4 1^D_2_— $37_{2}^{{}^{\circ}}$
741.207	.213	6	1:34915.1	5*p*^4 3^P_2_— 6*d*′ $5_{1}^{{}^{\circ}}$
743.054	.052	20	134579.7	5*p*^4 1^D_2_— $36_{2}^{{}^{\circ}}$
743.848		60	134436.1	$\left\{ \;\begin{array}{l} 5p^{4}{\;}^{3}P_{1}—8d{\;}^{3}D_{1}^{{}^{\circ}}\\ 5p^{4}{\;}^{3}P_{0}—8d{\;}^{5}D_{1}^{{}^{\circ}} \end{array}\right.$
744.188	.180	40	134374.6	5*p*^4 3^P_1_— 8*d* ${\;}^{3}D_{2}^{{}^{\circ}}$
744.822	.821	20	134260.3	5*p*^4 1^D_2_— $34_{2}^{{}^{\circ}}$
746.143	.136	1	134022.6	5*p*^4 3^P_2_— 6*d*′ $3_{3}^{{}^{\circ}}$
746.523	.517	15	133954.3	5*p*^4 3^P_2_— 6*d*′ $2_{3}^{{}^{\circ}}$
748.781	.780	800	133550.4	5*p*^4 3^P_2_— 7*s*′ ${\;}^{3}D_{3}^{{}^{\circ}}$
748.978	.978	7	133515.3	5*p*^4 1^D_2_— $30_{2}^{{}^{\circ}}$
749.552	.550	15	133413.0	5*p*^4 3^P_1_— 6*d*′ $15_{1}^{{}^{\circ}}$
750.197		250	133298.3	$\left\{ \;\begin{array}{l} 5p^{4}{\;}^{3}P_{2}—8s{\;}^{3}S_{1}^{{}^{\circ}}\\ 5p^{4}{\;}^{3}P_{2}—6d\prime \;1_{2}^{{}^{\circ}} \end{array}\right.$
751.187	.185	25	133122.6	5*p*^4 1^D_2_— $26_{3}^{{}^{\circ}}$
752.656	.660	6	132862.8	5*p*^4 1^D_2_—11*s* ${\;}^{3}S_{1}^{{}^{\circ}}$
752.798	.801	7	132837.8	5*p*^4 3^P_2_— 8*s* ${\;}^{5}S_{2}^{{}^{\circ}}$
753.263	.273	40	132755.8	5*p*^41^D_2_— $24_{1,\;2}^{{}^{\circ}}$
753.364	.381	3	132738.0	5*p*^4 3^P_2_—5*d*″ ${\;}^{3}P_{1}^{{}^{\circ}}$
754.342	.348	1	132565.9	5*p*^41^D_2_— $21_{1}^{{}^{\circ}}$
754.540	.546	8	132531.1	5*p*^4 3^P_0_— 6*d*′ $12_{1}^{{}^{\circ}}$
754.913	.918	35	132465.6	5*p*^4 3^P_1_— 6*d*′ $13_{1,\;2}^{{}^{\circ}}$
756.935	.942	2	132111.7	5*p*^4 1^D_2_— $20_{1,\;2}^{{}^{\circ}}$
758.201	.203	25	131891.1	5*p*^4 3^P_1_— 6*d*′ $12_{1}^{{}^{\circ}}$
761.739	.747	120	131278.6	5*p*^4 3^P_1_— 6*d*′ $11_{0}^{{}^{\circ}}$
763.644	.649	12	130951.1	5*p*^4 1^D_2_—8*s*′ ${\;}^{3}D_{2}^{{}^{\circ}}$
765.594		600	130617.5	$\left\{ \;\begin{array}{l} 5p^{4}{\;}^{3}P_{2}—\;7s\prime {\;}^{3}D_{2}^{{}^{\circ}}\\ 5p^{4}{\;}^{3}P_{2}—\;7s\prime {\;}^{3}D_{1}^{{}^{\circ}} \end{array}\right.$
770.771	.777	300	129740.2	5*p*^4 3^P_0_—7*d* ${\;}^{3}D_{1}^{{}^{\circ}}$
772.078	.086	1	129520.6	5*p*^4 1^D_2_—7*s*″ ${\;}^{3}P_{1}^{{}^{\circ}}$
772.344	.351	400	129476.0	5*p*^4 3^P_1_— 6*d*′ $7_{1,\;2}^{{}^{\circ}}$
773.055	.058	500	129356.9	5*p*^4 3^P_1_— 6*d*′ $6_{1,\;2}^{{}^{\circ}}$
786.413	.409	15	127159.6	5*p*^4 1^D_2_— 8*d* ${\;}^{5}D_{1}^{{}^{\circ}}$
786.460		20	127152.0	$\left\{ \;\begin{array}{l} 5p^{4}{\;}^{3}P_{1}—\;6d\prime \;4_{0}^{{}^{\circ}}\\ 5p^{4}{\;}^{1}D_{2}—8d\;{\;}^{5}D_{3}^{{}^{\circ}} \end{array}\right.$
788.329	.338	3	126850.6	5*p*^4 3^P_0_—8*s* ${\;}^{3}S_{1}^{{}^{\circ}}$
788.815	.811	700	126772.4	5*p*^4 1^D_2_— 6*d*′ $15_{1}^{{}^{\circ}}$
791.147	.149	350	126398.8	5*p*^4 1^D_2_—9*s* ${\;}^{3}S_{1}^{{}^{\circ}}$
791.851	.847	7	126286.4	5*p*^4 3^P_0_— 5*d*″ ${\;}^{3}P_{1}^{{}^{\circ}}$
791.967		15	126267.9	
792.313		70	126212.7	$\left\{ \;\begin{array}{l} 5p^{4}{\;}^{3}P_{1}—\;6d\prime \;1_{2}^{{}^{\circ}}\\ 5p^{4}{\;}^{3}P_{1}—\;8s\;{\;}^{3}S_{1}^{{}^{\circ}} \end{array}\right.$
794.237	.238	500	125907.0	5*p*^4 1^D_2_— 6*d*′ $14_{3}^{{}^{\circ}}$
794.757	.758	400	125824.6	5*p*^4 1^D_2_— 6*d*′ $13_{1,\;2}^{{}^{\circ}}$
797.819	.836	2	125341.7	5*p*^4 3^P_2_— 6*d* ${\;}^{3}D_{1}^{{}^{\circ}}$
798.158	.159	1000	125288.5	5*p*^4 3^P_2_— 6*d* ${\;}^{3}D_{3}^{{}^{\circ}}$
798.427	.419	400	125246.3	5*p*^4 3^P_2_— 6*d* ${\;}^{3}D_{2}^{{}^{\circ}}$
802.618	.637	1	124592.3	5*p*^4 1^D_2_— 6*d*′ $10_{2}^{{}^{\circ}}$
805.361	.365	5	124167.9	5*p*^4 3^P_0_— 7*s*′ ${\;}^{3}D_{1}^{{}^{\circ}}$
809.188	.192	60	123580.7	5*p*^4 3^P_2_— 5*d*″ ${\;}^{1}F_{3}^{{}^{\circ}}$
809.523		150	123529.5	$\left\{ \;\begin{array}{l} 5p^{4}{\;}^{3}P_{1}—\;7s\prime \;{\;}^{3}D_{2}^{{}^{\circ}}\\ 5p^{4}{\;}^{3}P_{1}—\;7s\prime \;{\;}^{3}D_{1}^{{}^{\circ}} \end{array}\right.$
814.094	.103	50	122835.9	5*p*^4 1^D_2_— 6*d*′ $7_{1,\;2}^{{}^{\circ}}$
814.459	.476	3	.122780.9	5*p*^4 3^P_2_— 5*d*″ ${\;}^{3}F_{2}^{{}^{\circ}}$
814.880	.888	12	122717.4	5*p*^4 1^D_2_— 6*d*′ $6_{1,\;2}^{{}^{\circ}}$
818.047	.039	700	122242.4	5*p*^4 3^P_2_— 6*d* ${\;}^{5}D_{1}^{{}^{\circ}}$
820.884	.892	150	121819.9	5*p*^4 3^P_2_— 6*d* ${\;}^{5}D_{2}^{{}^{\circ}}$
821.159	.164	30	121779.1	5*p*^4 3^P_2_— 6*d* ${\;}^{5}D_{3}^{{}^{\circ}}$
823.426	.436	170	121443.8	5*p*^4 3^P_0_— 5*d*″ ${\;}^{1}P_{1}^{{}^{\circ}}$
824.841	.858	1	121235.5	5*p*^4 1^D_2_— 7*d* ${\;}^{3}D_{2}^{{}^{\circ}}$
825.122	.113	500	121194.2	5*p*^4 3^P_2_— 5*d*″ ${\;}^{3}F_{3}^{{}^{\circ}}$
825.934	.929	300	121075.0	5*p*^4 3^P_2_— 5*d*″ ${\;}^{3}D_{1}^{{}^{\circ}}$
827.797	.792	400	120802.6	5*p*^4 3^P_1_— 5*d*″ ${\;}^{1}P_{1}^{{}^{\circ}}$
828.888	.897	1	120643.6	5*p*^4 1^D_2_— 7*d* ${\;}^{5}D_{2}^{{}^{\circ}}$
831.168	.187	300	120312.6	5*p*^4 1^D_2_— 7*s*′ ${\;}^{1}D_{2}^{{}^{\circ}}$
831.270	.278	150	120297.8	5*p*^4 1^D_2_— 6*d*′ $3_{3}^{{}^{\circ}}$
834.095	.106	1200	119890.4	5*p*^4 3^P_2_— 7*s* ${\;}^{3}S_{1}^{{}^{\circ}}$
838.070	.075	700	119321.8	5*p*^4 3^P_2_— 5*d*″ ${\;}^{3}D_{2}^{{}^{\circ}}$
840.259	.282	3	119010.9	5*p*^4 1^D_2_— 5*d*″ ${\;}^{3}P_{1}^{{}^{\circ}}$
841.097	.106	700	118892.4	5*p*^4 3^P_0_— 6*d* ${\;}^{3}D_{1}^{{}^{\circ}}$
843.113	.116	600	118608.1	5*p*^4 3^P_2_— 7*s* ${\;}^{5}S_{2}^{{}^{\circ}}$
845.638	.652	25	118253.9	5*p*^4 3^P_1_— 6*d* ${\;}^{3}D_{1}^{{}^{\circ}}$
846.295	.306	900	118162.1	5*p*^4 3^P_1_— 6*d* ${\;}^{3}D_{2}^{{}^{\circ}}$
847.796		600	117952.9	5*p*^4 3^P_1_— 5*d*″ ${\;}^{3}P_{0}^{{}^{\circ}}?$
847.926	.922	1000	117934.8	5*p*^4 3^P_2_— 5*d*″ ${\;}^{3}P_{2}^{{}^{\circ}}$
855.494	.502	400	116891.5	5*p*^4 1^D_2_—7s′ ${\;}^{3}D_{2}^{{}^{\circ}}$
863.590	.590	600	115795.7	5*p*^4 3^P_0_— 6*d* ${\;}^{5}D_{1}^{{}^{\circ}}$
868.380	.383	750	115157.0	5*p*^4 3^P_1_— 6*d* ${\;}^{5}D_{1}^{{}^{\circ}}$
870.343	.341	900	114897.2	5*p*^4 3^P_2_— 5*d*′ ${\;}^{1}P_{1}^{{}^{\circ}}$
871.596	.599	30	114732.0	5*p*^4 3^P_1_— 6*d* ${\;}^{5}D_{2}^{{}^{\circ}}$
872.391	.388	800	114627.5	5*p*^4 3^P_0_— 5*d*″ ${\;}^{3}D_{1}^{{}^{\circ}}$
873.489	.495	1500	114483.4	5*p*^4 3^P_2_— 5*d*′ ${\;}^{3}P_{2}^{{}^{\circ}}$
875.941	.940	1000	114162.9	5*p*^4 1^D_2_— 5*d*″ ${\;}^{1}P_{1}^{{}^{\circ}}$
877.276	.279	800	113989.2	5*p*^4 3^P_1_— 5*d*″ ${\;}^{3}D_{1}^{{}^{\circ}}$
879.844	.847	2000	113656.5	5*p*^4 3^P_2_— 5*d*″ ${\;}^{3}D_{3}^{{}^{\circ}}$
881.881	.889	1500	113394.0	5*p*^4 3^P_2_— 5*d*′ ${\;}^{1}F_{3}^{{}^{\circ}}$
886.492	.511	30	112804.2	5*p*^4 3^P_1_—7*s* ${\;}^{3}S_{1}^{{}^{\circ}}$
890.995	.995	1000	112234.1	5*p*^4 3^P_1_— 5*d*″ ${\;}^{3}D_{2}^{{}^{\circ}}$
893.167	.163	1000	111961.1	5*p*^4 3^P_2_— 6*s*″ ${\;}^{1}P_{1}^{{}^{\circ}}$
895.844	.847	400	111626.6	5*p*^4 3^P_0_— 5*d*′ ${\;}^{3}P_{1}^{{}^{\circ}}$
895.957	.963	200	111612.5	5*p*^4 1^D_2_— 6*d* ${\;}^{3}D_{1}^{{}^{\circ}}$
896.376	.370	400	111560.3	5*p*^4 1^D_2_— 6*d* ${\;}^{3}D_{3}^{{}^{\circ}}$
896.692		400	111521.0	$\left\{ \;\begin{array}{l} 5p^{4}{\;}^{1}D_{2}—6d{\;}^{3}D_{2}^{{}^{\circ}}\\ 5p^{4}{\;}^{3}P_{1}—7s{\;}^{5}S_{2}^{{}^{\circ}} \end{array}\right.$
901.004	.006	200	110987.3	5*p*^4 3^P_1_— 5*d*′ ${\;}^{3}P_{1}^{{}^{\circ}}$
902.130	.133	400	110848.8	5*p*^4 3^P_1_— 5*d*″ ${\;}^{3}P_{2}^{{}^{\circ}}$
905.313	.314	100	110459.0	5*p*^4 3^P_2_— 6*s*″ ${\;}^{3}P_{2}^{{}^{\circ}}$
910.309	.309	400	109852.8	5*p*^4 1^D_2_— 5*d*″ ${\;}^{1}F_{3}^{{}^{\circ}}$
914.957	.951	200	109294.8	5*p*^4 3^P_2_— 5*d*′ ${\;}^{3}S_{1}^{{}^{\circ}}$
917.007	.001	300	109050.4	5*p*^4 1^D_2_— 5*d*″ ${\;}^{3}F_{2}^{{}^{\circ}}$
921.343	.340	400	108537.2	5*p*^4 3^P_2_— 5*d*′ ${\;}^{1}D_{2}^{{}^{\circ}}$
922.085	.088	100	108449.9	5*p*^4 3^P_0_— 5*d*′ ${\;}^{1}P_{1}^{{}^{\circ}}$
925.119	.142	1	108094.2	5*p*^4 1^D_2_— 6*d* ${\;}^{5}D_{2}^{{}^{\circ}}$
925.477	.488	15	108052.4	5*p*^4 1^D_2_— 6*d* ${\;}^{5}D_{3}^{{}^{\circ}}$
929.143	.144	800	107626.0	5*p*^4 3^P_2_— 5*d*′ ${\;}^{3}G_{3}^{{}^{\circ}}$
930.133	.131	100	107511.5	5*p*^4 3^P_2_— 5*d*′ ${\;}^{3}F_{2}^{{}^{\circ}}$
930.506	.506	150	107468.4	5*p*^4 1^D_2_— 5*d*″ ${\;}^{3}F_{3}^{{}^{\circ}}$
931.139	.137	100	107395.4	5*p*^4 3^P_1_— 5*d*′ ${\;}^{3}P_{2}^{{}^{\circ}}$
937.322	.332	20	106686.9	5*p*^4 1^S_0_—7*d* ${\;}^{3}D_{1}^{{}^{\circ}}$
940.904	.902	200	106280.8	5*p*^4 3^P_1_— 5*d*″ ${\;}^{1}D_{2}^{{}^{\circ}}$
941.936	.960	2	106164.3	5*p* ^4 1^D_2_—7*s* ${\;}^{3}S_{1}^{{}^{\circ}}$
942.472	.478	40	106103.9	5*p*^4 3^P_2_— 6*s*″ ${\;}^{3}P_{1}^{{}^{\circ}}$
947.023	.024	100	105594.0	5*p*^4 1^D_2_— 5*d*″ ${\;}^{3}D_{2}^{{}^{\circ}}$
947.749	.744	15	105513.2	5*p*^4 3^P_0_— 6*s*″ ${\;}^{1}P_{1}^{{}^{\circ}}$
948.658	.653	80	105412.1	5*p*^4 1^S_0_— 6*d*′ $5_{1}^{{}^{\circ}}$
953.199		8	104909.9	
953.489		30*d*	104878.0	$\left\{ \;\begin{array}{l} 5p^{4}{\;}^{3}P_{1}—6s\prime \prime \;{\;}^{1}P_{1}^{{}^{\circ}}\\ 5p^{4}{\;}^{1}D_{2}—7s\;{\;}^{5}S_{2}^{{}^{\circ}} \end{array}\right.$
956.503	.515	10	104547.5	5*p*^4 3^P_2_— 5*d*′ ${\;}^{3}D_{1}^{{}^{\circ}}$
958.333	.342	25	104347.9	5*p*^4 1^D_2_— 5*d*′ ${\;}^{3}P_{1}^{{}^{\circ}}$
959.619	.617	100	104208.0	5*p*^4 1^D_2_— 5*d*″ ${\;}^{3}P_{2}^{{}^{\circ}}$
961.979	.982	70	103952.4	5*p*^4 3^P_1_— 5*d*′ ${\;}^{1}S_{0}^{{}^{\circ}}$
963.415	.430	4	103797.4	5*p*^4 1^S_0_—8*s* ${\;}^{3}S_{1}^{{}^{\circ}}$
967.378	.381	700	103372.2	5*p*^4 3^P_1_— 6*s*″ ${\;}^{3}P_{2}^{{}^{\circ}}$
968.670	.677	100	103234.3	5*p*^4 1^S_0_— 5*d*″ ${\;}^{3}P_{1}^{{}^{\circ}}$
972.292	.313	10	102849.8	5*p*^4 3^P_0_— 5*d*′ ${\;}^{3}S_{1}^{{}^{\circ}}$
978.394	.393	300	102208.3	5*p*^4 3^P_1_— 5*d*′ ${\;}^{3}S_{1}^{{}^{\circ}}$
988.434	.433	200	101170.1	5*p*^4 1^D_2_— 5*d*′ ${\;}^{1}P_{1}^{{}^{\circ}}$
992.508	.503	150	100754.8	5*p*^4 1^D_2_— 5*d*′ ${\;}^{3}P_{2}^{{}^{\circ}}$
995.768	.770	700	100425.0	5*p*^4 3^P_1_— 5*d*′ ${\;}^{3}F_{2}^{{}^{\circ}}$
1000.572	.569	1200	99942.8	5*p*^4 3^P_2_— 5*d*′ ${\;}^{3}F_{3}^{{}^{\circ}}$
1000.701	.711	80	99929.9	5*p*^4 1^D_2_— 5*d*″ ${\;}^{3}D_{3}^{{}^{\circ}}$
1003.350	.354	1000	99666.1	5*p*^4 1^D_2_— 5*d*′ ${\;}^{1}F_{3}^{{}^{\circ}}$
1003.457	.458	500	99655.5	5*p*^4 3^P_0_— 6*s*″ ${\;}^{3}P_{1}^{{}^{\circ}}$
1003.612	.605	300	99640.1	5*p*^4 1^D_2_— 5*d*″ ${\;}^{1}D_{2}^{{}^{\circ}}$
1009.936	.935	700	99016.2	5*p*^4 3^P_1_— 6*s*″ ${\;}^{3}P_{1}^{{}^{\circ}}$
1016.381	.375	20	98388.3	5*p*^4 1^S_0_— 5*d*″ ${\;}^{1}P_{1}^{{}^{\circ}}$
1017.977	.973	200	98234.0	5*p*^4 1^D_2_— 6*s*″ ${\;}^{1}P_{1}^{{}^{\circ}}$
1018.53	.581	4000	98175.6	5*p*^4 3^P_2_— 5*d*′ ${\;}^{3}D_{3}^{{}^{\circ}}$
1019.230	.223	400	98113.3	5*p*^4 3^P_1_— 6*s*″ ${\;}^{3}P_{0}^{{}^{\circ}}$
1019.400	.386	800	98096.9	5*p*^4 3^P_0_— 5*d*′ ${\;}^{3}D_{1}^{{}^{\circ}}$
1023.544	.533	200	97699.8	5*p*^4 3^P_2_— 6*s*′ ${\;}^{1}D_{2}^{{}^{\circ}}$
1026.065	.070	350	97459.7	5*p*^4 3^P_1_— 5*d*′ ${\;}^{3}D_{1}^{{}^{\circ}}$
1030.047	.043	800	97082.9	5*p*^4 3^P_2_— 5*d*′ ${\;}^{3}D_{2}^{{}^{\circ}}$
1033.801	.787	400	96730.4	5*p*^4 1^D_2_— 6*s*″ ${\;}^{3}P_{2}^{{}^{\circ}}$
1034.655	.655	10000	96650.6	5*p*^4 3^P_2_— 6*s*′ ${\;}^{3}D_{3}^{{}^{\circ}}$
1042.154	.146	100	95955.1	5*p*^4 3^P_2_— 5*p*^5^ ${\;}^{1}P_{1}^{{}^{\circ}}$
1043.430	.431	2	95837.8	5*p* ^4 1^S_0_— 6*d* ${\;}^{3}D_{1}^{{}^{\circ}}$
1046.373	.373	200	95568.2	5*p*^4 1^D_2_— 5*d*′ ${\;}^{3}S_{1}^{{}^{\circ}}$
1054.582	.571	150	94824.3	5*p*^4 3^P_2_— 6*s*′ ${\;}^{3}D_{1}^{{}^{\circ}}$
1054.742	.738	1500	94809.9	5*p*^4 1^D_2_— 5*d*′ ${\;}^{1}D_{2}^{{}^{\circ}}$
1064.981	.977	60	93898.4	5*p*^4 1^D_2_— 5*d*′ ${\;}^{3}G_{3}^{{}^{\circ}}$
1066.273	.273	250	93784.6	5*p*^4 1^D_2_— 5*d*′ ${\;}^{3}F_{2}^{{}^{\circ}}$
1067.341	.334	2000	93690.8	5*p*^4 3^P_2_— 6*s*′ ${\;}^{3}D_{2}^{{}^{\circ}}$
1075.210	.200	3000	93005.1	5*p*^4 3^P_2_— 5*d* ${\;}^{3}D_{3}^{{}^{\circ}}$
1082.540	.531	400	92375.3	5*p*^4 1^D_2_— 6*s*″ ${\;}^{3}P_{1}^{{}^{\circ}}$
1085.405	.405	700	92131.5	5*p*^4 3^P_2_— 5*d* ${\;}^{3}D_{1}^{{}^{\circ}}$
1101.099	.091	30	90818.4	5*p*^4 1^D_2_— 5*d*′ ${\;}^{3}D_{1}^{{}^{\circ}}$
1101.239	.232	400	90806.8	5*p*^4 3^P_1_— 5*d*′ ${\;}^{3}P_{0}^{{}^{\circ}}$
1103.590	.584	800	90613.4	5*p*^4 3^P_1_— 6*s*′ ${\;}^{1}D_{2}^{{}^{\circ}}$
1105.000	4.993	5000	90497.7	5*p*^4 3^P_2_— 5*d* ${\;}^{3}D_{2}^{{}^{\circ}}$
1111.165	.157	2500	89995.6	5*p*^4 3^P_1_— 5*d*′ ${\;}^{3}D_{2}^{{}^{\circ}}$
1117.219	.219	1500	89508.0	5*p*^4 3^P_0_— 5*p*^5^ ${\;}^{1}P_{1}^{{}^{\circ}}$
1125.251	.254	3500	88869.1	5*p*^4 3^P_1_— 5*p*^5^ ${\;}^{1}P_{1}^{{}^{\circ}}$
1131.504	.511	2000	88378.0	5*p*^4 3^P_0_— 6*s*′ ${\;}^{3}D_{1}^{{}^{\circ}}$
1139.752	.753	1200	87738.4	5*p*^4 3^P_1_— 6*s*′ ${\;}^{3}D_{1}^{{}^{\circ}}$
1139.805	.808	10000	87734.3	5*p*^4 3^P_2_— 5*d* ${\;}^{5}D_{1}^{{}^{\circ}}$
1154.668	.670	1500	86605.0	5*p*^4 3^P_1_— 6*s*′ ${\;}^{3}D_{2}^{{}^{\circ}}$
1159.871	.879	1000	86216.5	5*p*^4 1^D_2_— 5*d*′ ${\;}^{3}F_{3}^{{}^{\circ}}$
1160.562	·565	10000	86165.2	5*p*^4 3^P_2_— 5*d* ${\;}^{5}D_{2}^{{}^{\circ}}$
1166.482	.480	20000	85727.9	5*p*^4 3^P_2_— 5*d* ${\;}^{5}D_{3}^{{}^{\circ}}$
1167.054	.069	1500	85685.8	5*p*^4 3^P_0_— 5*d* ${\;}^{3}D_{1}^{{}^{\circ}}$
1171.007	.014	50	85396.6	5*p*^4 1^S_0_— 5*d*′ ${\;}^{1}P_{1}^{{}^{\circ}}$
1175.841	.840	5000	85045.5	5*p*^4 3^P_1_— 5*d* ${\;}^{3}D_{1}^{{}^{\circ}}$
1178.650	.649	10000	84842.8	5*p*^4 3^P_2_— 6*s* ${\;}^{3}S_{1}^{{}^{\circ}}$
1184.156	.153	200	84448.3	5*p*^4 1^D_2_— 5*d*′ ${\;}^{3}D_{3}^{{}^{\circ}}$
1187.338	.335	15000	84222.0	5*p*^4 3^P_2_— 5*p*^5^ ${\;}^{3}P_{1}^{{}^{\circ}}$
1190.853	.850	10000	83973.4	5*p*^4 1^D_2_— 6*s*′ ${\;}^{1}D_{2}^{{}^{\circ}}$
1198.884	.878	5000	83410.9	5*p*^4 3^P_1_— 5*d* ${\;}^{3}D_{2}^{{}^{\circ}}$
1199.677	.672	1000	83355.8	5*p*^4 1^D_2_— 5*d*′ ${\;}^{3}D_{2}^{{}^{\circ}}$
1200.223	.221	7000	83317.8	5*p*^4 3^P_1_— 5*p*^5^ ${\;}^{3}P_{0}^{{}^{\circ}}$
1205.931	.933	2000	82923.5	5*p*^4 1^D_2_— 6*s*′ ${\;}^{3}D_{3}^{{}^{\circ}}$
1216.125	.121	3000	82228.4	5*p*^4 1^D_2_— 5*p*^5^ ${\;}^{1}P_{1}^{{}^{\circ}}$
1220.887	.885	20000	81907.7	5*p*^4 3^P_2_— 5*d*^5^ ${\;}^{3}P_{2}^{{}^{\circ}}$
1230.222	.221	600	81286.1	5*p*^4 3^P_0_— 5*d* ${\;}^{5}D_{1}^{{}^{\circ}}$
1233.069	.074	100	81098.5	5*p*^4 1^D_2_— 6*s*′ ${\;}^{3}D_{1}^{{}^{\circ}}$
1234.063	.070	20000	81033.1	5*p*^4 3^P_2_— 6*s* ${\;}^{5}S_{2}^{{}^{\circ}}$
1239.969	.970	80	80647.2	5*p*^4 3^P_1_— 5*d* ${\;}^{5}D_{1}^{{}^{\circ}}$
1250.559	.560	700	79964.2	5*p*^4 1^D_2_— 6*s*′ ${\;}^{3}D_{2}^{{}^{\circ}}$
1264.580	.576	20	79077.6	5*p*^4 3^P_1_— 5*d* ${\;}^{5}D_{2}^{{}^{\circ}}$
1275.424	.422	2000	78405.3	5*p*^4 1^D_2_— 5*d* ${\;}^{3}D_{1}^{{}^{\circ}}$
1275.596	.592	1000	78394.7	5*p*^4 3^P_0_— 6*s* ${\;}^{3}S_{1}^{{}^{\circ}}$
1277.190	.186	2500	78296.9	5*p*^4 3^P_1_— 5*d* ${\;}^{5}D_{0}^{{}^{\circ}}$
1285.782	.772	1500	77773.7	5*p*^4 3^P_0_—5*p*^5^ ${\;}^{3}P_{1}^{{}^{\circ}}$
1286.084	.076	4000	77755.4	5*p*^4 3^P_1_— 6*s* ${\;}^{3}S_{1}^{{}^{\circ}}$
1296.416	.425	3000	77135.7	5*p*^4 3^P_1_— 5*d*^5^ ${\;}^{3}P_{1}^{{}^{\circ}}$
1302.580	.573	150	76770.7	5*p*^4 1^D_2_— 5*d* ${\;}^{3}D_{2}^{{}^{\circ}}$
1332.533	.536	12	75045.0	5*p*^4 1^S_0_— 5*d*′ ${\;}^{3}D_{1}^{{}^{\circ}}$
1336.517	.527	20000	74821.3	5*p*^4 3^P_1_—5*p*^5^ ${\;}^{3}P_{2}^{{}^{\circ}}$
1351.219	.22.5	20	74007.2	5*p*^4 1^D_2_— 5*d* ${\;}^{5}D_{1}^{{}^{\circ}}$
1380.501	.497	500	72437.5	5*p*^4 1^D_2_— 5*d* ${\;}^{5}D_{2}^{{}^{\circ}}$
1406.138	.159	10	71116.8	5*p*^4 1^D_2_— 6*s* ${\;}^{3}S_{1}^{{}^{\circ}}$
1418.539	.540	25	70495.1	5*p*^4 1^D_2_—5*p*^5^ ${\;}^{3}P_{1}^{{}^{\circ}}$
1466.673	.693	1000	68181.5	5*p*^4 1^D_2_—5*p*^5^ ${\;}^{3}P_{2}^{{}^{\circ}}$
1485.734	.763	30	67306.8	5*p*^4 1^D_2_— 6*s* ${\;}^{5}S_{2}^{{}^{\circ}}$
1504.761	.788	10	66455.7	5*p*^4 1^S_0_—5*p*^5^ ${\;}^{1}P_{1}^{{}^{\circ}}$
1521.068		15	65743.3	
1700.529		2	58805.2	
1825.049	.059	2	54793.0	5*p*^5^ ${\;}^{3}P_{2}^{{}^{\circ}}—8p$ ^3^P_2_
1826.705		10	54743.4	
1828.550		3	54688.1	
1828.729		3	54682.8	
1859.466		3	53778.9	
1961.837	.839	2	50972.6	5*d* ${\;}^{5}D_{3}^{{}^{\circ}}—8p$ ^3^P_2_
1964.631	.636	3	50900.1	5*d* ${\;}^{3}D_{2}^{{}^{\circ}}—6f$ ^3^F_3_
1978.816	.799	2	50535.3	5*d* ${\;}^{5}D_{0}^{{}^{\circ}}—8p$ ^3^P_2_
1981.360		3	50470.4	
1990.944		3	50227.4	

λ(air)	Intensity	Wave number	Classification
2014.178	1	49632.00	5*p*^5^ ${\;}^{3}P_{0}^{{}^{\circ}}—$ 4*f*′ ^3^D_1_
2023.100	8	49413.17	5*d* ${\;}^{5}D_{3}^{{}^{\circ}}—$ 5*f* ^5^F_4_
2024.580	2	49377.05	5*d*′ ${\;}^{5}D_{2}^{{}^{\circ}}—$ 1_2,3_
2026.155	1	49338.67	
2034.068	15	49146.76	5*d* ${\;}^{5}D_{4}^{{}^{\circ}}—$ 5*f* ^5^F_5_
2035.812	1	49104.66	5*d* ${\;}^{5}D_{4}^{{}^{\circ}}—$ 5*f* ^5^F_4_
2037.806	2	49056.62	5*d* ${\;}^{5}D_{2}^{{}^{\circ}}—$ 5*f* ^5^F_2_
2038.561	1	49038.46	
2041.256	4	48973.72	5*d* ${\;}^{5}D_{2}^{{}^{\circ}}—$ 5*f* ^5^F_3_
2048.816	1	48793.04	5*d* ${\;}^{5}D_{1}^{{}^{\circ}}—$ 4*f*′ ^3^F_2_
2053.160	1	48689.82	
2053.802	1	48674.60	5*d* ${\;}^{5}D_{1}^{{}^{\circ}}—$ 8*p* ^3^P_1_
2060.167	2	48524.24	
2091.300	3	47801.95	
2096.797	7	47676.65	$\left\{ \;\begin{array}{l} 5d\prime \;{\;}^{3}F_{4}^{{}^{\circ}}—\;8f\;{\;}^{3}F_{3}\\ 6p\;{\;}^{5}P_{3}—\;35_{2,3}^{{}^{\circ}} \end{array}\right.$
2103.562	1*h*	47523.34	5*p*^5^ ${\;}^{3}P_{2}^{{}^{\circ}}—$ 6*p*″ ^3^D_3_
2105.120	1	47488.17	5*d*′ ${\;}^{3}G_{4}^{{}^{\circ}}—$ 4_3,4_
2114.745	6	47272.06	
2116.970	1	47222.38	5*p*^5^ ${\;}^{3}P_{1}^{{}^{\circ}}—$ 6*p*″ ^1^D_2_
2117.767	1	47204.61	
2118.690	1*h*	47184.05	
2119.858	2*h*	47158.06	5*d* ${\;}^{3}D_{3}^{{}^{\circ}}—$ 7*p*′ ^1^F_3_
2122.086	8	47108.55	
2122.252	1	47104.87	
2122.372	1	47102.20	5*d* ${\;}^{3}D_{3}^{{}^{\circ}}—$ 7*p*′ ^3^F_4_
2125.156	1	47040.50	
2132.921	1	46869.27	6*s*′ ${\;}^{3}D_{2}^{{}^{\circ}}—$ 4*f*′ ^1^P_1_
2140.992	2	46692.61	6*s*′ ${\;}^{3}D_{2}^{{}^{\circ}}—$ 7*p*′ ^3^F_3_
2141.338	1*h*	46685.06	5*d* ${\;}^{3}D_{3}^{{}^{\circ}}—$ 4*f*′ ^3^G_3_
2143.876	1	46629.80	
2150.143	3	46493.90	
2171.863	2	46028.99	
2172.223	5	46021.36	
2177.480	4	45910.26	5*d* ${\;}^{3}D_{2}^{{}^{\circ}}—$ 8*p* ^3^P_1_
2203.313	8*h*	45372.04	
2208.736	1	45260.65	$\left\{ \;\begin{array}{l} 6p{\;}^{5}P_{2}—\;9d{\;}^{5}D_{3}^{{}^{\circ}}\\ 5d{\;}^{3}D_{1}^{{}^{\circ}}—\;4f\prime {\;}^{3}P_{1} \end{array}\right.$
2210.358	3	45227.44	5*d* ${\;}^{3}D_{2}^{{}^{\circ}}—$ 5*f* ^3^F_2_
2210.876	2	45216.85	
2211.135	1	45211.55	6*s*′ ${\;}^{3}D_{1}^{{}^{\circ}}—$ 4*f*′ ^3^D_1_
2211.533	1	45203.42	6*s* ${\;}^{5}S_{2}^{{}^{\circ}}—$ 7*p* ^3^P_1_
2217.279	2	45086.28	6*s*′ ${\;}^{3}D_{1}^{{}^{\circ}}—$ 7*p*′ ^3^F_2_
2227.385	1	44881.74	5*d* ${\;}^{3}D_{1}^{{}^{\circ}}—$ 4*f*′ ^3^D_2_
2229.246	30	44844.28	5*d* ${\;}^{3}D_{2}^{{}^{\circ}}—$ 5*f* ^3^F_3_
2230.393	2	44821.22	
2231.371	1	44801.58	
2236.618	1	44696.48	6*p* ^5^P_2_—10*s* ${\;}^{5}S_{2}^{{}^{\circ}}$
2236.762	1	44693.60	6*p* ^3^P_2_—10*d* ${\;}^{3}D_{3}^{{}^{\circ}}$
2239.433	3	44640.30	5*d* ${\;}^{3}D_{2}^{{}^{\circ}}—$ 5*f* ^5^F_3_
2239.714	1*h*	44634.70	
2244.666	5	44536.24	
2246.675	2*h*	44496.42	5*p*^5^ ${\;}^{3}P_{2}^{{}^{\circ}}—$7*p* ^3^P_2_
2255.206	1*h*	44328.12	5*p*^5^ ${\;}^{3}P_{2}^{{}^{\circ}}—$7*p* ^3^P_1_
2256.674	3*h*	44299.28	
2260.035	20	44233.41	5*p*^5^ ${\;}^{1}P_{1}^{{}^{\circ}}—$7*p*′ ^1^D_2_
2261.700	4	44200.85	
2262.032	2*h*	44194.36	6*s* ${\;}^{5}S_{2}^{{}^{\circ}}—$4*f* ^3^F_3_
2262.533	1*h*	44184.58	
2267.855	2*h*	44080.90	5*p*^5^ ${\;}^{1}P_{1}^{{}^{\circ}}—$4*f*′ ^3^D_1_
2274.322	3	43955.57	5*p*^5^ ${\;}^{1}P_{1}^{{}^{\circ}}—$7*p*′ ^3^F_2_
2276.270	1*h*	43917.95	
2286.564	4	43720.26	6*s* ${\;}^{3}S_{1}^{{}^{\circ}}—$ 6*p*″ ^3^S_1_
2287.060	1	43710.77	5*d* ${\;}^{5}D_{1}^{{}^{\circ}}—$ 6*p*″ ^1^D_2_
2287.903	15	43694.67	5*d* ${\;}^{3}D_{3}^{{}^{\circ}}—$ 8*p* ^3^P_2_
2288.569	1	43681.96	
2288.693	2	43679.59	
2291.767	2	43621.01	6*p* ^5^P_3_— 10*s* ${\;}^{5}S_{2}^{{}^{\circ}}$
2293.226	25	43593.26	5*d* ${\;}^{3}D_{1}^{{}^{\circ}}—$ 5*f* ^3^F_2_
2302.945	1	43409.30	
2310.139	5	43274.13	
2310.551	2	43266.41	5*d* ${\;}^{5}D_{2}^{{}^{\circ}}—$ 6*p*″ ^3^D_3_
2311.205	1*h*	43254.17	5*d*^5^ ${\;}^{3}P_{2}^{{}^{\circ}}—$ 6*p*″ ^3^P_1_
2314.761	2	43187.73	6*p* ^3^P_1_— 9*d* ${\;}^{3}D_{2}^{{}^{\circ}}$
2315.638	4	43171.38	
2319.150	1	43106.00	5*d*′ ${\;}^{3}D_{2}^{{}^{\circ}}—$ 7*p*′ ^1^D_2_
2319.968	2	43090.81	5*d* ${\;}^{3}D_{3}^{{}^{\circ}}—$ 4*f*′ ^3^F_3_
2320.329	10	43084.10	5*d* ${\;}^{3}D_{3}^{{}^{\circ}}—$ 4*f*′ ^3^G_4_
2322.532	2*c*	43043.24	
2327.395	1	42953.31	5*d*′ ${\;}^{3}D_{2}^{{}^{\circ}}—$ 4*f*′ ^3^D_1_
2331.650	3*c*	42874.93	
2333.076	1	42848.73	
2334.203	20	42828.04	5*d*′ ${\;}^{3}D_{2}^{{}^{\circ}}—$ 7*p*′ ^3^F_2_
2335.502	10*c*	42804.22	5*p*^5^ ${\;}^{3}P_{2}^{{}^{\circ}}—$ 4*f* ^5^F_3_
2330.188	1	42791.66	6*s*′ ${\;}^{1}D_{2}^{{}^{\circ}}—$ 4*f*′ ^1^D_2_
2339.831	1	42725.04	
2340.072	1	42720.64	
2340.277	3	42716.90	6*s*′ ${\;}^{3}D_{2}^{{}^{\circ}}—$ 8*p* ^3^P_1_
2340.778	3	42707.75	5*d*′ ${\;}^{3}F_{2}^{{}^{\circ}}—$ 10_2,3_
2341.947	1	42686.44	6*s*′ ${\;}^{1}D_{2}^{{}^{\circ}}—$ 4*f*′ ^1^F_3_
2342.402	50	42678.15	5*d* ${\;}^{3}D_{3}^{{}^{\circ}}—$ 5*f* ^3^F_4_
2343.067	3	42666.03	5*d*′ ${\;}^{3}P_{0}^{{}^{\circ}}—$ 4*f*′ ^1^P_1_
2344.303	1	42643.54	
2345.301	4	42625.40	5*d*′ ${\;}^{3}G_{3}^{{}^{\circ}}—$ 11_3,4_
2346.266	15	42607.86	5*d*′ ${\;}^{3}D_{2}^{{}^{\circ}}—$ 4*f*′ ^3^G_3_
2347.044	4	42593.74	5*d*′ ${\;}^{3}G_{3}^{{}^{\circ}}—$ 10_2,3_
2348.485	5	42567.61	6*s*′ ${\;}^{3}D_{1}^{{}^{\circ}}—$ 4*f*′ ^3^P_1_
2349.180	1	42555.02	
2350.115	1	42538.09	6*p* ^3^P_1_— 10*s* ${\;}^{3}S_{1}^{{}^{\circ}}$
2352.860	3	42488.46	6*s*′ ${\;}^{1}D_{2}^{{}^{\circ}}—$ 7*p*′ ^1^D_2_
2354.286	8	42462.73	6*s*′ ${\;}^{1}D_{2}^{{}^{\circ}}—$ 7*p*′ ^1^F_3_
2357.473	3	42405.33	6*s*′ ${\;}^{3}D_{2}^{{}^{\circ}}—$ 4*f*′ ^3^F_3_
2359.865	2	42360.56	5*d*′ ${\;}^{3}P_{0}^{{}^{\circ}}—$ 7*p*′ ^1^P_1_
2360.474	7	42351.42	6*p* ^3^P_2_— 9*d* ${\;}^{3}D_{3}^{{}^{\circ}}$
2361.174	10	42338.87	6*s* ${\;}^{3}S_{1}^{{}^{\circ}}—$ 7*p* ^3^P_0_
2361.303	4	42336.56	5*d* ${\;}^{3}D_{3}^{{}^{\circ}}—$ 5*f* ^3^F_3_
2362.399	4	42316.92	
2362.946	1	42307.12	6*p* ^5^P_2_— 8*d* ${\;}^{3}D_{3}^{{}^{\circ}}$
2363.303	1	42300.73	6*p* ^5^P_1_— 8*d* ${\;}^{3}D_{1}^{{}^{\circ}}$
2367.224	3	42230.67	5*d* ${\;}^{3}D_{1}^{{}^{\circ}}—$ 6*p*″ ^1^S_0_
2367.930	1	42218.08	
2368.332	7*c*	42210.92	
2369.574	20	42188.79	
2369.960	10	42181.92	5*p*^5^ ${\;}^{3}P_{1}^{{}^{\circ}}—$ 7*p* ^3^P_2_
2371.622	1	42152.36	5*d′* ${\;}^{3}F_{4}^{{}^{\circ}}—$ 6*f* ^3^F_4_
2372.192	15	42142.24	5*d′* ${\;}^{3}P_{0}^{{}^{\circ}}—$ 4*f*′ ^3^D_1_
2372.596	3	42135.06	5*d* ${\;}^{3}D_{3}^{{}^{\circ}}—$ 5*f* ^5^F_4_
2378.034	10	42038.72	5*d* ${\;}^{5}D_{1}^{{}^{\circ}}—$ 6*p*″ ^3^P_2_
2379.463	5	42013.47	5*d*′ ${\;}^{3}D_{3}^{{}^{\circ}}—$ 7*p*′ ^1^D_2_
2380.761	1	41990.57	6*s*′ ${\;}^{1}D_{2}^{{}^{\circ}}—$ 4*f*′ ^3^G_3_
2380.917	2	41987.82	5*d*′ ${\;}^{3}D_{3}^{{}^{\circ}}—$ 7*p*′ ^1^F_3_
2384.069	20	41932.31	5*d*′ ${\;}^{3}D_{3}^{{}^{\circ}}—$ 7*p*′ ^3^F_4_
2395.244	25*c*	41736.09	
2397.260	25	41701.59	6*s*′ ${\;}^{3}D_{1}^{{}^{\circ}}—$ 4*p*′ ^3^F_2_
2397.753	3	41693.02	5*d*′ ${\;}^{3}F_{2}^{{}^{\circ}}—$ 7_2,3_
2398.225	10*d*	41684.81	5*p*^5^ ${\;}^{1}P_{1}^{{}^{\circ}}—$ 4*f*′ ^3^P_2_
2398.370	4	41682.29	5*d*′ ${\;}^{1}D_{2}^{{}^{\circ}}—$ 10_2,3_
2399.209	4	41667.72	6*p* ^5^P_1_— 8*d* ${\;}^{5}D_{1}^{{}^{\circ}}$
2400.166	6	41651.11	6*p* ^5^P_1_— 8*d* ${\;}^{5}D_{2}^{{}^{\circ}}$
2400.588	3	41643.79	6*p* ^5^P_1_— 8*d* ${\;}^{5}D_{0}^{{}^{\circ}}$
2400.974	1	41637.09	6*p* ^3^P_0_— 8*s*′ ${\;}^{3}D_{1}^{{}^{\circ}}$
2403.863	1	41587.06	5*p*′ ${\;}^{3}F_{3}^{{}^{\circ}}—$ 6*f* ^3^F_4_
2404.090	20	41583.13	6*s*′ ${\;}^{3}D_{1}^{{}^{\circ}}—$ 8*p* ^3^P_1_
2404.926	2	41568.67	6*p* ^3^P_2_— 10*s* ${\;}^{3}S_{1}^{{}^{\circ}}$
2405.384	3*d*	41560.76	6*p* ^5^P_2_— 8*d* ${\;}^{5}D_{1}^{{}^{\circ}}$
2405.887	20	41552.07	6*p* ^5^P_2_— 8*d* ${\;}^{5}D_{3}^{{}^{\circ}}$
2406.303	10	41543.33	$\left\{ \begin{array}{l} 5d\prime {\;}^{3}D_{3}^{{}^{\circ}}—4f\prime {\;}^{1}G_{4}\\ 6p{\;}^{5}P_{2}—8d{\;}^{5}D_{2}^{{}^{\circ}} \end{array}\right.$
2408.008	100	41515.48	5*d*′ ${\;}^{3}D_{3}^{{}^{\circ}}—$ 4*f*′ ^3^G_3_
2411.967	1	41447.34	6*s*′ ${\;}^{3}D_{2}^{{}^{\circ}}—$ 5*f* ^5^F_3_
2412.570	40*d*	41436.98	
2414.154	1	41409.79	
2415.117	3	41393.28	
2415.732	2	41382.74	
2417.853	3	41346.44	5*d*′ ${\;}^{3}G_{3}^{{}^{\circ}}—$ 6_2,3,4_
2419.176	100	41323.83	5*d*′ ${\;}^{3}D_{3}^{{}^{\circ}}—$ 4*f*′ ^3^F_4_
2434.830	4	41058.18	
2437.087	1	41020.15	5*d*′ ${\;}^{3}G_{3}^{{}^{\circ}}—$ 4_3,4_
2437.710	5	41009.67	5*d*′ ${\;}^{3}F_{4}^{{}^{\circ}}—$ 4*f*′ ^1^F_3_
2437.901	20	41006.46	5*d*′ ${\;}^{3}F_{4}^{{}^{\circ}}—$ 7*p*′ ^3^F_3_
2438.285	25	41000.00	5*p*^5^ ${\;}^{3}P_{1}^{{}^{\circ}}—$ 6*p*″ ^3^D_2_
2441.484	3	40946.28	5*d* ${\;}^{3}D_{2}^{{}^{\circ}}—$ 6*p*″ ^1^D_2_
2441.872	2	40939.78	5*p*^5^ ${\;}^{3}P_{1}^{{}^{\circ}}—$ 6*p*″ ^3^P_1_
2444.220	40	40900.45	6*s*′ ${\;}^{3}D_{1}^{{}^{\circ}}—$ 5*f* ^3^F_2_
2446.516	3	40862.07	5*p*^5^ ${\;}^{3}P_{1}^{{}^{\circ}}—$ 7*p* ^5^P_2_
2448.496	30	40829.03	5*d* ${\;}^{5}D_{1}^{{}^{\circ}}—$ 6*p*″ ^3^S_1_
2451.075	4	40786.08	5*d*′ ${\;}^{3}F_{4}^{{}^{\circ}}—$ 7*p*′ ^1^F_3_
2451.194	5	40784.10	5*p*^5^ ${\;}^{3}P_{1}^{{}^{\circ}}—$ 6*p*″ ^3^P_0_
2453.952	5	40738.26	6*p* ^5^P_1_— 9*s* ${\;}^{5}S_{2}^{{}^{\circ}}$
2454.434	2	40730.26	
2454.533	30	40728.62	5*p*^5^ ${\;}^{3}P_{1}^{{}^{\circ}}—$ 7*p* ^5^P_1_
2454.958	3	40721.57	
2456.046	1*h*	40703.53	
2457.367	2	40681.65	6*s* ${\;}^{3}S_{1}^{{}^{\circ}}—$ 4*f* ^3^F_2_
2457.703	6	40676.09	5*d* ${\;}^{5}D_{3}^{{}^{\circ}}—$ 7*p* ^3^P_2_
2458.239	5*h*	40667.22	5*d*′ ${\;}^{1}D_{2}^{{}^{\circ}}—$ 7_2,3_
2459.005	2	40654.55	
2460.444	8	40630.78	6*p* ^5^P_2_— 9*s* ${\;}^{5}S_{2}^{{}^{\circ}}$
2461.126	30	40619.52	5*p*^5^ ${\;}^{3}P_{1}^{{}^{\circ}}—$ 4*f* ^5^F_1_
2464.085	15*c*	40570.74	
2464.684	80	40560.89	5*p*^5^ ${\;}^{3}P_{1}^{{}^{\circ}}—$ 4*f* ^5^F_2_
2466.249	1	40535.15	
2469.167	20	40487.25	6*p* ^5^P_3_— 8*d* ${\;}^{5}D_{4}^{{}^{\circ}}$
2469.824	6	40476.48	6*p* ^5^P_3_— 8*d* ${\;}^{5}D_{3}^{{}^{\circ}}$
2470.369	1	40467.55	6*p* ^5^P_3_— 8*d* ${\;}^{5}D_{2}^{{}^{\circ}}$
2471.295	25*d*	40452.39	
2471.664	2	40446.35	
2473.272	2	40420.06	5*p*^5^ ${\;}^{3}P_{0}^{{}^{\circ}}—$ 6*p*″ ^1^P_1_
2475.766	1	40379.34	6*s* ${\;}^{3}S_{1}^{{}^{\circ}}—$ 6*p*″ ^3^D_2_
2476.213	2	40372.05	5*d*′ ${\;}^{3}G_{4}^{{}^{\circ}}—$ 6*f* ^3^F_4_
2476.738	1	40363.50	
2478.073	5*c*	40341.75	5*d*′ ${\;}^{3}F_{4}^{{}^{\circ}}—$ 4*f*′ ^1^G_4_
2478.994	2	40326.76	5*d* ${\;}^{3}D_{2}^{{}^{\circ}}—$ 6*p*″ ^1^P_1_
2479.467	3	40319.07	6*s* ${\;}^{3}S_{1}^{{}^{\circ}}—$ 6*p*″ ^3^P_1_
2479.797	6	40313.71	5*d*′ ${\;}^{3}F_{4}^{{}^{\circ}}—$ 4*f*′ ^3^G_3_
24.80.029	2	40309.94	
2480.48G	1	40302.51	
2481.103	25	40292.49	5*d*′ ${\;}^{1}D_{2}^{{}^{\circ}}—$ 5_2,3_
2481.289	1	40289.47	
2481.689	2	40282.98	
2482.058	25	40276.98	5*d*′ ${\;}^{3}D_{2}^{{}^{\circ}}—$ 7*p*′ ^3^D_3_
2483.261	1	40257.48	
2483.520	3*c*	40253.28	5*p*^5^ ${\;}^{1}P_{1}^{{}^{\circ}}—$ 7*p*′ ^3^D_1_
2483.951	1	40246.29	5*p*′ ${\;}^{3}F_{3}^{{}^{\circ}}—$ 7*p*′ ^1^D_2_
2484.246	1	40241.52	6*s* ${\;}^{3}S_{1}^{{}^{\circ}}—$ 7*p* ^5^P_2_
2484.387	2	40239.23	5*d* ${\;}^{5}D_{2}^{{}^{\circ}}—$ 7*p* ^3^P_2_
2487.027	10	40196.52	
2489.065	10*c*	40163.61	6*s* ${\;}^{3}S_{1}^{{}^{\circ}}—$ 6*p*″ ^3^P_0_
2491.638	50	40122.14	5*d*′ ${\;}^{3}F_{4}^{{}^{\circ}}—$ 4*f*′ ^3^F_4_
2492.514	8	40108.04	6*s* ${\;}^{3}S_{1}^{{}^{\circ}}—$ 7*p* ^5^P_1_
2493.458	3	40092.86	
2494.738	100	40072.29	5*d*′ ${\;}^{3}F_{4}^{{}^{\circ}}—$ 4*f*′ ^3^G_5_
2496.123	2*c*	40050.05	6*s*′ ${\;}^{3}D_{3}^{{}^{\circ}}—$ 8*p* ^3^P_2_
2497.984	1	40020.22	
2499.316	30	39998.89	6*s* ${\;}^{3}S_{1}^{{}^{\circ}}—$ 4*f* ^5^F_1_
2502.981	70	39940.32	6*s* ${\;}^{3}S_{1}^{{}^{\circ}}—$ 4*f* ^5^F_2_
2503.560	10	30931.09	$\left\{ \begin{array}{l} 5d\prime \;3D_{2}^{{}^{\circ}}—\;4f\prime {\;}^{3}D_{2}\\ 5d\prime \;3G_{4}^{{}^{\circ}}—\;6f{\;}^{5}F_{5} \end{array}\right.$
2506.999	6	39876.32	$\left\{ \begin{array}{l} 6s\prime {\;}^{3}D_{3}^{{}^{\circ}}—\;4f\prime {\;}^{3}F_{2}\\ 6p{\;}^{3}P_{1}—\;8d{\;}^{3}D_{1}^{{}^{\circ}} \end{array}\right.$
2508.941	1	39845.45	
2510.612	8	39818.94	6*p* ^3^P_1_— 8*d* ${\;}^{3}D_{2}^{{}^{\circ}}$
2513.199	20	39777.95	5*d* ${\;}^{5}D_{0}^{{}^{\circ}}—$ 6*p*″ ^3^P_1_
2514.617	10	39755.52	5*d* ${\;}^{5}D_{3}^{{}^{\circ}}—$ 7*p* ^5^P_3_
2515.022	10	39749.12	5*d* ${\;}^{5}D_{3}^{{}^{\circ}}—$ 4*f* ^3^F_4_
2519.494	5	39678.57	
2519.898	1	39672.21	6*s*′ ${\;}^{1}D_{2}^{{}^{\circ}}—$ 7*p*′ ^3^P_2_
2521.989	1	39639.32	
2525.206	3	39588.82	
2525.988	4	39576.57	5*d*′ ${\;}^{3}D_{2}^{{}^{\circ}}—$ 7*p*′ ^3^P_1_
2526.612	8	39566.79	5*d* ${\;}^{5}D_{0}^{{}^{\circ}}—$7*p* ^5^P_1_
2527.350	10	39555.24	6*p* ^5^P_3_— 9*s* ${\;}^{5}S_{2}^{{}^{\circ}}$
2530.974	50	39498.61	5*d*′ ${\;}^{3}P_{0}^{{}^{\circ}}—$ 4*f*′ ^3^P_1_
2533.372	5	39461.22	5*d*′ ${\;}^{3}F_{2}^{{}^{\circ}}—$ 2_2,3_
2533.605	100	39457.59	5*d* ${\;}^{5}D_{0}^{{}^{\circ}}—$ 4*f* ^5^F_1_
2534.272	200	39447.21	5*d* ${\;}^{5}D_{4}^{{}^{\circ}}—$ 7*p* ^5^P_3_
2534.505	40	39443.58	5*d*′ ${\;}^{3}D_{2}^{{}^{\circ}}—$ 4*f*′ ^3^F_2_
2535.708	2	39424.87	
2535.846	2	39422.73	5*d*′ ${\;}^{3}F_{2}^{{}^{\circ}}—$ 8*f* ^5^F_3_
2536.994	4	39404.89	5*d*′ ${\;}^{1}D_{2}^{{}^{\circ}}—$ 3_2,3_
2537.583	2	39395.74	
2540.126	40	39356.31	5*d* ${\;}^{5}D_{3}^{{}^{\circ}}—$ 7*p* ^5^P_2_
2542.140	60*d*	39325.13	5*d*′ ${\;}^{3}D_{2}^{{}^{\circ}}—$ 8*p* ^3^P_1_
2542.557	3	39318.68	5*d* ${\;}^{5}D_{2}^{{}^{\circ}}—$ 7*p* ^5^P_3_
2542.976	1	39312.20	5*d* ${\;}^{3}D_{1}^{{}^{\circ}}—$ 6*p*″ ^1^D_2_
2545.416	20	39274.52	5*d* ${\;}^{3}D_{2}^{{}^{\circ}}—$ 6*p*″ ^3^P_2_
2548.342	15*c*	39229.43	5*d′* ${\;}^{3}G_{4}^{{}^{\circ}}—$ 4*f*′ ^1^F_3_
2548.556	25*c*	39226.13	$\left\{ \begin{array}{l} 5d\prime {\;}^{3}G_{4}^{{}^{\circ}}—\;7p\prime {\;}^{3}F_{3}\\ 6p{\;}^{3}P_{1}—\;8d{\;}^{5}D_{2}^{{}^{\circ}} \end{array}\right.$
2550.426	4	39197.37	5*d′* ${\;}^{3}D_{3}^{{}^{\circ}}—$ 7*p*′ ^3^P_2_
2551.258	6	39184.59	5*d′* ${\;}^{3}D_{3}^{{}^{\circ}}—$ 7*p*′ ^3^D_3_
2552.448	1	39166.32	
2557.699	50	39085.92	5*d′* ${\;}^{3}D_{2}^{{}^{\circ}}—$ 4*f*′ ^3^D_3_
2559.258	25	39062.11	5*d* ${\;}^{5}D_{2}^{{}^{\circ}}—$ 4*f* ^3^F_3_
2559.571	10	39057.34	5*d* ${\;}^{5}D_{2}^{{}^{\circ}}—$ 6*p*″ ^3^D_2_
2559.708	20	39055.25	5*d* ${\;}^{5}D_{3}^{{}^{\circ}}—$ 4*f* ^5^F_2_
2561.132	5*c*	39033.53	6*s*′ ${\;}^{3}D_{3}^{{}^{\circ}}—$ 5*f* ^3^F_4_
2561.977	30	39020.66	6*p* ^3^P_2_— 8*d* ${\;}^{3}D_{3}^{{}^{\circ}}$
2562.451	300*c*	39013.44	5*d*′ ${\;}^{3}D_{2}^{{}^{\circ}}—$ 4*f*′ ^3^F_3_
2562.701	2	39009.64	
2562.945	2	39005.92	5*d*′ ${\;}^{3}G_{4}^{{}^{\circ}}—$ 7*p*′ ^1^F_3_
2563.350	10*c*	38999.76	6*s*′ ${\;}^{1}D_{2}^{{}^{\circ}}—$ 8*p* ^3^P_2_
2563.518	15	38997.20	5*d* ${\;}^{5}D_{2}^{{}^{\circ}}—$ 6*p*″ ^3^P_1_
2564.386	200	38984.00	5*d* ${\;}^{5}D_{3}^{{}^{\circ}}—$ 4*f* ^5^F_3_
2566.242	1000	38955.81	5*d* ${\;}^{5}D_{3}^{{}^{\circ}}—$ 4*f* ^5^F_4_
2566.604	2	38950.32	5*d*′ ${\;}^{3}G_{4}^{{}^{\circ}}—$ 7*p*′ ^3^F_4_
2566.712	3	38948.68	5*d*′ ${\;}^{3}F_{2}^{{}^{\circ}}—$ 1_2,3_
25G7.529	3	38936.29	
2568.642	8	38919.42	5*d* ${\;}^{5}D_{2}^{{}^{\circ}}—$ 7*p* ^5^P_2_
2572.956	5	38854.16	
2573.253	5	38849.68	6*p* ^3^P_2_— 8*d* ${\;}^{3}D_{2}^{{}^{\circ}}$
2573.992	5	38838.53	5*d*′ ${\;}^{3}D_{3}^{{}^{\circ}}—$ 4*f*′ ^3^D_2_
2574.256	1	38834.54	5*d*′ ${\;}^{3}D_{3}^{{}^{\circ}}—$ 1_2,3_
2574.817	1	38826.08	5*s*′ ${\;}^{1}D_{2}^{{}^{\circ}}—$ 4*f*′ ^3^F_2_
2577.473	1	38786.08	5*d* ${\;}^{5}D_{2}^{{}^{\circ}}—$ 7*p* ^5^P_1_
2577.652	2	38783.38	
2580.145	5*c*	38745.91	6*s*″ ${\;}^{3}P_{2}^{{}^{\circ}}—$ 7_2,3_
2582.794	2000	38706.18	5*d* ${\;}^{5}D_{4}^{{}^{\circ}}—$ 4*f* ^5^F_5_
2583.710	2	38692.46	5*d* ${\;}^{3}D_{1}^{{}^{\circ}}—$ 6*p*″ ^1^P_1_
2584.746	10	38676.95	5*d* ${\;}^{5}D_{2}^{{}^{\circ}}—$ 4*f* ^5^F_1_
2584.837	8	38675.59	5*d* ${\;}^{5}D_{4}^{{}^{\circ}}—$ 4*f* ^5^F_3_
2585.202	5	38670.12	5*d* ${\;}^{5}D_{1}^{{}^{\circ}}—$ 7*p* ^3^P_2_
2585.407	2*c*	38667.06	
2586.721	100	38647.42	5*d* ${\;}^{5}D_{4}^{{}^{\circ}}—$ 4*f* ^5^F_4_
2587.052	4*c*	38642.48	5*d*′ ${\;}^{3}D_{2}^{{}^{\circ}}—$ 5*f* ^3^F_2_
2588.132	8*d*	38626.35	5*d*′ ${\;}^{3}D_{3}^{{}^{\circ}}—$ 4*f*′ ^3^H_4_
2588.670	150	38618.32	5*d* ${\;}^{5}D_{2}^{{}^{\circ}}—$ 4*f* ^5^F_2_
2592.010	2*h*	38568.56	6*p*′ ^3^F_2_ — $41_{1,2,3}^{{}^{\circ}}$
2592.490	3*c*	38561.42	5*d*′ ${\;}^{3}G_{4}^{{}^{\circ}}—$ 4*f*′ ^1^G_4_
2593.458	300	38547.03	5*d* ${\;}^{5}D_{2}^{{}^{\circ}}—$ 4*f* ^5^F_3_
2594.376	4	38533.39	5*d*′ ${\;}^{3}G_{4}^{{}^{\circ}}—$ 4*f*′ ^3^G_3_
2594.954	20*c*	38524.81	5*d*′ ${\;}^{3}D_{3}^{{}^{\circ}}—$ 8*p* ^3^P_2_
2595.518	8	38516.44	6*p* ^3^P_0_—8*d* ${\;}^{3}D_{1}^{{}^{\circ}}$
2596.063	4*h*	38508.35	5*s*′ ${\;}^{1}D_{2}^{{}^{\circ}}—$ 7*p*′ ^3^D_1_
2596.500	6	38501.87	5*d* ${\;}^{5}D_{1}^{{}^{\circ}}—$ 7*p* ^3^P_1_
2597.878	6	38481.45	6*p* ^3^P_1_—9*s* ${\;}^{3}S_{1}^{{}^{\circ}}$
2598.397	4	38473.77	
2598.765	4	38468.32	6*s*′ ${\;}^{1}D_{2}^{{}^{\circ}}—$ 4*f*′ ^3^D_3_
2600.757	1	38438.86	5*d* ${\;}^{3}D_{3}^{{}^{\circ}}—$ 6*p*″ ^1^D_2_
2600.989	2	38435.43	
2602.890	10*c*	38407.36	5*p*^5^ ${\;}^{1}P_{1}^{{}^{\circ}}—$ 6*p*″ ^1^S_0_
2603.658	8*h*	38396.03	6*s*′ ${\;}^{1}D_{2}^{{}^{\circ}}—$ 4*f*′ ^3^F_3_
2605.340	3	38371.24	6*s*″ ${\;}^{3}P_{2}^{{}^{\circ}}—$ 5_2,3_
2606.703	2	38351.18	5*d*′ ${\;}^{3}D_{3}^{{}^{\circ}}—$ 4*f*′ ^3^F_2_
2607.347	30	38341.71	5*d*′ ${\;}^{3}G_{4}^{{}^{\circ}}—$ 4*f*′ ^3^F_4_
2608.442	1	38325.62	
2609.175	1	38314.85	5*d*′ ${\;}^{3}P_{0}^{{}^{\circ}}—$ 7*p*′ ^3^D_1_
2610.740	150	38291.88	5*d*′ ${\;}^{3}G_{4}^{{}^{\circ}}—$ 4*f*′ ^3^G_5_
2612.524	1	38265.74	6*p* ^3^P_2_—8*d* ${\;}^{5}D_{3}^{{}^{\circ}}$
2612.963	20	38259.31	5*d*′ ${\;}^{3}D_{2}^{{}^{\circ}}—$ 5*f* ^3^F_3_
2619.890	30	38158.16	5*d*^5^ ${\;}^{3}P_{0}^{{}^{\circ}}—$ 6*p*″ ^3^S_1_
2621.362	4*c*	38136.73	5*d* ${\;}^{5}D_{0}^{{}^{\circ}}—$ 6*p*″ ^3^D_1_
2621.858	3	38129.52	
2626.309	2	38064.90	5*d* ${\;}^{3}D_{2}^{{}^{\circ}}—$ 6*p*″ ^3^S_1_
2626.974	8	38055.26	5*d*′ ${\;}^{3}D_{2}^{{}^{\circ}}—$ 5*f* ^5^F_3_
2627.334	8	38050.05	6*s* ${\;}^{5}S_{2}^{{}^{\circ}}—$ 6*p*′ ^1^D_2_
2631.983	15	3792.84	5*d*′ ${\;}^{3}F_{4}^{{}^{\circ}}—$ 7*p*′ ^3^D_3_
2632.756	1	37971.69	5*d*′ ${\;}^{1}G_{4}^{{}^{\circ}}—$ 6*f* ^5^F_4_
2635.298	1	37935.07	
2636.270	20*w*	37921.08	5*d*′ ${\;}^{3}D_{3}^{{}^{\circ}}—$ 4*f*′ ^3^F_3_
2636.729	150*w*	37914.48	5*d*′ ${\;}^{3}D_{3}^{{}^{\circ}}—$ 4*f*′ ^3^G_4_
2638.644	1	37886.96	6*p* ^3^P_2_— 6*d*′ $15_{1}^{{}^{\circ}}$
2641.302	20	37848.84	
2645.375	2	37790.57	5*d* ${\;}^{5}D_{1}^{{}^{\circ}}—$ 4*f* ^3^F_2_
2651.301	1	37706.11	
2651.874	1*h*	37697.96	5*d*′ ${\;}^{3}F_{3}^{{}^{\circ}}—$ 4*f*′ ^3^P_2_
2652.636	1*h*	37687.13	6*d*′ ^3^D_2_— $41_{1,2,3}^{{}^{\circ}}$
2655.829	10*c*	37641.82	6*s*′ ${\;}^{1}D_{2}^{{}^{\circ}}—$ 5*f* ^3^F_3_
2660.396	1	37577.21	
2665.014	5	37512.10	6*p* ^3^P_2_— 9*s* ${\;}^{3}S_{1}^{{}^{\circ}}$
2665,284	20	37508.30	5*d*′ ${\;}^{3}D_{3}^{{}^{\circ}}—$ 5*f* ^3^F_4_
2666.141	1	37496.24	6*p* ^3^P_0_— 6*d*′ $15_{1}^{{}^{\circ}}$
2667,049	3	37483.48	6*s*″ ${\;}^{3}P_{2}^{{}^{\circ}}—$ 3_2,3_
2667.624	1	37475.40	
2670.304	4*d*	37437.79	6*s*′ ${\;}^{1}D_{2}^{{}^{\circ}}—$ 5*f* ^5^F_3_
2670.854	4	37430.08	5*d*′ ${\;}^{3}F_{3}^{{}^{\circ}}—$ 7*p*′ ^3^P_2_
2671.008	5	37427.92	5*d* ${\;}^{3}D_{1}^{{}^{\circ}}—$ 6*p*″ ^3^P_1_
2671.239	6	37424.69	5*d*′ ${\;}^{3}F_{4}^{{}^{\circ}}—$ 4*f*′ ^3^H_4_
2671.770	1	37417.25	5*d*′ ${\;}^{3}F_{3}^{{}^{\circ}}—$ 7*p*′ ^3^D_3_
2674.795	150	37374.94	5*d*′ ${\;}^{3}F_{4}^{{}^{\circ}}—$ 4*f*′ ^3^H_5_
2676.150	3*c*	37356.01	5*d* ${\;}^{5}D_{2}^{{}^{\circ}}—$ 6*p*″ ^3^D_1_
2676.562	10	37350.26	5*d* ${\;}^{5}D_{1}^{{}^{\circ}}—$ 7*p* ^5^P_2_
2676.983	1	37344.39	6*p* ^3^P_2_— 9*s* ${\;}^{5}S_{2}^{{}^{\circ}}$
2681.203	10	37285.62	5*d*′ ${\;}^{1}G_{4}^{{}^{\circ}}—$ 4*f*′ ^1^F_3_
2681.597	10	37280.14	5*d*″ ${\;}^{1}D_{2}^{{}^{\circ}}—$ 13_2,3_
2682.160	15	37272.31	5*d* ${\;}^{5}D_{1}^{{}^{\circ}}—$ 6*p*″ ^3^P_0_
2683.392	3	37255.20	5*d*′ ${\;}^{1}F_{3}^{{}^{\circ}}—$ 13_2,3_
2685.160	2*c*	37230.67	
2686.154	20	37216.90	5*d* ${\;}^{5}D_{1}^{{}^{\circ}}—$ 7*p* ^5^P_1_
2687.164	1*c*	37202.91	
2688.536	5	37183.92	
2688.981	200*c*	37177.77	
2689.192	30*c*	37174.85	5*d*^5^ ${\;}^{3}P_{2}^{{}^{\circ}}—$ 6*p*′ ^1^D_2_
2689.722	1*c*	37167.53	
2691.652	1	37140.88	
2693.040	1*h*	37121.74	6*p* ^3^P_0_— 9*s* ${\;}^{3}S_{1}^{{}^{\circ}}$
2694.056	25	37107.74	5*d* ${\;}^{5}D_{1}^{{}^{\circ}}—$ 4*f* ^5^F_1_
2694.593	1*c*	37100.35	
2696.702	6	37071.33	5*d*′ ${\;}^{3}F_{3}^{{}^{\circ}}—$ 4*f*′ ^3^D_2_
2697.375	2	37062.08	5*d*′ ${\;}^{1}G_{4}^{{}^{\circ}}—$ 7*p*′ ^1^F_3_
2698.319	50	37049.12	5*d* ${\;}^{5}D_{1}^{{}^{\circ}}—$ 4*f* ^5^F_2_
2700.419	3	37020.31	6*p* ^3^P_1_— 6*d*′ $14_{3}^{{}^{\circ}}$
2701.431	50	37006.44	5*d*′ ${\;}^{1}G_{4}^{{}^{\circ}}—$ 7*p*′ ^3^F_4_
2704.257	1	36967.77	
2704.424	2*c*	36965.49	
2704.522	20*c*	36964.15	
2706.320	3*c*	36939.59	
2707.400	3	36924.86	5*d*′ ${\;}^{3}D_{1}^{{}^{\circ}}—$ 6*f* ^3^F_2_
2708.159	50	36914.51	
2709.708	1	36893.41	
2710.206	1	36886.63	5*d*′ ${\;}^{1}F_{3}^{{}^{\circ}}—$ 12_1,2_?
2710.857	1	36877.77	
2712.236	150	36859.02	5*d*′ ${\;}^{3}F_{3}^{{}^{\circ}}—$ 4*f*′ ^3^H_4_
2713.108	1	36847.18	6*p*′ ^3^F_3_— $39_{4}^{{}^{\circ}}$
2715.784	4	36810.87	
2719.726	20	36757.52	5*d*′ ${\;}^{3}F_{3}^{{}^{\circ}}—$ 8*p* ^3^P_2_
2722.542	2*c*	36719.50	6*p*′ ^3^D_2_— $38_{1}^{{}^{\circ}}$
2723.041	40*c*	36712.77	5*d*′ ${\;}^{3}F_{4}^{{}^{\circ}}—$ 4*f*′ ^3^G_4_
2726.649	2	36664.20	
2728.749	6	36635.98	
2730.124	500	36617.53	5*d*′ ${\;}^{1}G_{4}^{{}^{\circ}}—$ 4*f*′ ^1^G_4_
2731.788	20	36595.23	$\left\{ \begin{array}{l} 6s\prime \prime {\;}^{3}P_{2}^{{}^{\circ}}—\;8f{\;}^{3}F_{3}\\ 5d\prime \prime {\;}^{3}D_{3}^{{}^{\circ}}—\;11_{3,4} \end{array}\right.$
2732.200	5	36589.71	5*d*′ ${\;}^{1}G_{4}^{{}^{\circ}}—$ 4*f*′ ^3^G_3_
2732.735	10	36582.55	6*p*′ ^3^D_1_— 11*s* ${\;}^{3}S_{1}^{{}^{\circ}}$
2734.137	3	36563.79	6*p*′ ^3^F_4_— $50_{3,4}^{{}^{\circ}}$
2734.877	1	36553.90	6*p*′ ^1^P_1_— $46_{2}^{{}^{\circ}}$
2736.410	1	36533.42	6*p*′ ^1^P_1_— $45_{1,2}^{{}^{\circ}}$
2736.938	1	36526,37	
2744.533	20	36425.30	5*d* ${\;}^{3}D_{3}^{{}^{\circ}}—$ 6*p*″ ^3^D_3_
2746,590	8	36398.02	5*d*′ ${\;}^{1}G_{4}^{{}^{\circ}}—$ 4*f*′ ^3^F_4_
2747.502	4	36385.94	
2749.133	1*c*	36364.35	6*p* ^3^P_1_— 6*d*′ $12_{1}^{{}^{\circ}}$
2749.884	2*c*	36354.42	
2750.362	2*c*	36348.10	5*d*′ ${\;}^{1}G_{4}^{{}^{\circ}}—$ 4*f*′ ^3^G_5_
2753.502	10*d*	36306.66	5*d*′ ${\;}^{3}F_{4}^{{}^{\circ}}—$ 5*f* ^3^F_4_
2754.745	1	36290.28	
2755.128	5	36285.23	6*p*′ ^3^D_1_— $21_{1}^{{}^{\circ}}$
2757.213	7	36257.79	6*p* ^5^P_2_— 7*d* ${\;}^{3}D_{3}^{{}^{\circ}}$
2758.702	1	36238.22	
2759.624	8	36226.12	5*d*′ ${\;}^{3}F_{3}^{{}^{\circ}}—$ 4*f*′ ^3^D_3_
2761.431	15	36202.41	5*d*′ ${\;}^{3}G_{4}^{{}^{\circ}}—$ 7*p*′ ^3^D_3_
2761.930	1*c*	36195.87	
2764.142	4*c*	36166.91	6*p*′ ^1^F_3_— $44_{2,3}^{{}^{\circ}}$
2764.250	2	36165.50	5*d*′ ${\;}^{3}P_{2}^{{}^{\circ}}—$ 13_2,3_
2765.149	20	36153.74	5*d*′ ${\;}^{3}F_{3}^{{}^{\circ}}—$ 4*f*′ ^3^F_3_
2765.660	200	36147.06	5*d*′ ${\;}^{3}F_{3}^{{}^{\circ}}—$ 4*f*′ ^3^G_4_
2769.734	2*c*	36093.89	
2770.695	20	36081.37	6*s*′ ${\;}^{3}D_{2}^{{}^{\circ}}—$ 6*p*″ ^3^P_2_
2775.306	1*h*	36021.43	
2775.613	1	36017.45	
2775.856	4	36014.29	5*d*′ ${\;}^{3}D_{1}^{{}^{\circ}}—$ 4*f*′ ^1^P_1_
2776.241	2	36009.30	6*p*′ ^5^F_2_— 10*d* ${\;}^{3}D_{3}^{{}^{\circ}}$
2776.990	4*c*	35999.59	6*s*′ ${\;}^{3}D_{1}^{{}^{\circ}}—$ 6*p*″ ^1^P_1_
2781.129	10*d*	35946.01	5*d*′ ${\;}^{3}D_{1}^{{}^{\circ}}—$ 4*f*′ ^1^D_2_
2782.793	1*h*	35924.52	
2783.290	8*c*	35918.11	
2784.243	10	35905.81	5*d* ${\;}^{3}D_{2}^{{}^{\circ}}—$ 7*p* ^3^P_2_
2788.236	1*c*	35854.40	
2788.696	1*h*	35848.48	
2788.911	5*c*	35845.72	6*p*′ ^1^F_3_— $42_{2}^{{}^{\circ}}$
2789.056	10	35843.85	6*p*′ ^3^F_2_— 10*d* ${\;}^{3}D_{2}^{{}^{\circ}}$
2789.601	30*c*	35836.85	5*d*″ ${\;}^{1}D_{2}^{{}^{\circ}}—$ 7_2,3_
2790.060	10	35830.96	6*p*′ ^3^D_1_— $20_{1,2}^{{}^{\circ}}$
2790.573	1*c*	35824.37	
2790.994	1*h*	35818.97	
2791.524	2*c*	35812.17	
2792.724	6	35796.78	5*d*′ ${\;}^{3}P_{2}^{{}^{\circ}}—$ 12_1,2_
2793.933	6*c*	35781.29	
2797.089	10*c*	35740.92	5*d*′ ${\;}^{3}F_{3}^{{}^{\circ}}—$ 5*f* ^3^F_4_
2797.367	20*c*	35737.37	
2799.632	6*c*	35708.46	
2804.766	8	35643.10	5*d*′ ${\;}^{3}D_{1}^{{}^{\circ}}—$ 7*p*′ ^1^D_2_
2805.564	4	35632.96	6*p* ^5^P_2_— 7*d* ${\;}^{3}D_{2}^{{}^{\circ}}$
2808.594	200	35594.52	5*d*′ ${\;}^{3}G_{4}^{{}^{\circ}}—$ 4*f*′ ^3^H_5_
2809.789	10	35579.38	5*d*′ ${\;}^{1}F_{3}^{{}^{\circ}}—$ 6_2,3,4_
2811.927	1*c*	35552.33	
2812.210	4*c*	35548.75	5*d*″ ${\;}^{3}D_{3}^{{}^{\circ}}—$ 7_2,3_
2812.692	2	35542.66	
2815.118	3	35512.03	
2816.821	15	35490.56	5*d*′ ${\;}^{3}D_{1}^{{}^{\circ}}—$ 4*f*′ ^3^D_1_
2817.090	1*c*	35487.18	6*p*′ ^1^F_3_ — 12*d* ${\;}^{3}D_{3}^{{}^{\circ}}$
2817.909	1	35476.86	
2819.086	25	35462.05	5*d*″ ${\;}^{1}D_{2}^{{}^{\circ}}—$ 5_2,3_
2823.629	1*c*	35405.00	6*p*′ ^1^F_3_ — $33_{3}^{{}^{\circ}}$
2824.060	10	35399.60	5*d*′ ${\;}^{3}F_{3}^{{}^{\circ}}—$ 5*f* ^3^F_3_
2825.461	4	35382.04	5*d*′ ${\;}^{1}P_{1}^{{}^{\circ}}—$ 12_1,3_
2826.004	3	35375.24	
2826.814	30	35365.11	5*d*′ ${\;}^{3}D_{1}^{{}^{\circ}}—$ 7*p*′ ^3^F_2_
2829.338	1	35333.56	
2829.837	8*c*	35327.33	6*p* ^5^P_1_ — 7*d* ${\;}^{5}D_{1}^{{}^{\circ}}$
2830.110	30	35323.92	6*p*′ ^1^F_3_ — $40_{3}^{{}^{\circ}}$
2830.735	100	35316.12	5*d*″ ${\;}^{3}D_{3}^{{}^{\circ}}—$ 6_2,3,4_
2832.494	1	35294.19	
2832.736	3	35291.18	6*p*′ ^3^F_2_ — 11*s* ${\;}^{3}S_{1}^{{}^{\circ}}$
2833.495	20	35281.73	6*p* ^5^P_1_ — 7*d* ${\;}^{5}D_{0}^{{}^{\circ}}$
2836.768	10	35241.02	
2838.482	1*h*	35219.74	6*p* ^5^P_2_ — 7*d* ${\;}^{5}D_{1}^{{}^{\circ}}$
2840.236	6	35197.99	5*d*′ ${\;}^{3}F_{3}^{{}^{\circ}}—$ 5*f* ^5^F_4_
2840.438	6	35195.49	5*d*′ ${\;}^{3}F_{3}^{{}^{\circ}}—$ 5*f* ^5^F_3_
2840.970	25	35188.90	
2841.444	20	35183.03	6*p*′ ^3^F_2_ — $24_{1,2}^{{}^{\circ}}$
2841.920	1	35177.14	
2842.175	7	35173.98	5*d*″ ${\;}^{3}D_{3}^{{}^{\circ}}—$ 5_2,3_
2844.121	25	35149.92	6*p* ^5^P_1_ — 7*d* ${\;}^{5}D_{2}^{{}^{\circ}}$
2846.986	50	35114.54	6*p* ^5^P_2_ — 7*d* ${\;}^{5}D_{3}^{{}^{\circ}}$
2847.442	20	35108.92	6*p*′ ^1^F_3_ — $39_{4}^{{}^{\circ}}$
2848.272	1	35098.69	6*p*′ ^3^D_3_ — $46_{2}^{{}^{\circ}}$
2849.671	10	35081.46	
2851.170	1*c*	35063.02	6*p*′ ^3^D_2_ — $30_{2}^{{}^{\circ}}$
2851.723	7	35056.22	6*p*′ ^3^D_3_ — $44_{2.3}^{{}^{\circ}}$
2852.060	20*c*	35052.08	6*s* ${\;}^{5}S_{2}^{{}^{\circ}}—$ 6*p*′ ^3^P_1_
2852.290	8	35049.25	5*d* ${\;}^{3}D_{1}^{{}^{\circ}}—$ 7*p* ^3^P_0_
2852.852	25	35042.35	6*p* ^5^P_2_ — 7*d* ${\;}^{5}D_{2}^{{}^{\circ}}$
2854.168	80	35026.19	5*d* ${\;}^{3}D_{2}^{{}^{\circ}}—$ 4*f* ^3^F_2_
2854.964	4	35016.42	6*p* ^5^P_1_ — 6*d*′ $4_{0}^{{}^{\circ}}$
2856.798	8	34993.95	6*p*′ ^3^F_2_ — $21_{1}^{{}^{\circ}}$
2857.132	15*c*	34989.86	5*d*″ ${\;}^{3}D_{3}^{{}^{\circ}}—$ 4_3,4_
2857.515	35	34985.17	5*d* ${\;}^{3}D_{2}^{{}^{\circ}}—$ 7*p*; ^5^P_3_
2859.376	20	34962.40	6*p*′ ^3^D_2_ *—* 10*d*; ${\;}^{3}D_{2}^{{}^{\circ}}$
2860.602	35	34947.41	6*s*′ ${\;}^{3}D_{1}^{{}^{\circ}}—$6*p*″; ^3^P_2_
2861.290	3	34939.01	5*d*′ ${\;}^{3}G_{4}^{{}^{\circ}}—$ 4*f*′ ^3^F_3_
2861.838	40	34932.32	5*d*′ ${\;}^{3}G_{4}^{{}^{\circ}}—$ 4*f*′ ^3^G_4_
2865.184	6	34891.53	6*p* ^3^P_2_ — 6*d*′ $9_{3}^{{}^{\circ}}$
2865.458	4	34888.19	6*p*′ ^3^F_2_ — 10*d* ${\;}^{3}D_{3}^{{}^{\circ}}$
2865.766	5*d*	34884.44	5*d*′ ${\;}^{3}P_{2}^{{}^{\circ}}—$ 8_1,2_
2867.021	20*c*	34869.17	5*p*^5^ ${\;}^{1}P_{1}^{{}^{\circ}}—$ 6*p*″ ^1^P_1_
2867.704	1*c*	34860.87	
2867.970	2*c*	34857.64	
2869.767	7	34835.81	6*s*″ ${\;}^{3}P_{0}^{{}^{\circ}}—$ 4*f*′ ^3^D_1_
2870.635	2	34825.28	6*p*′ ^3^D_1_—9*d* ${\;}^{3}D_{2}^{{}^{\circ}}$
2871.416	1	34815.80	
2872.788	7	34799.18	6*p* ^3^P_1_—6*d*′ $6_{1,2}^{{}^{\circ}}$
2873.208	5*c*	34794.09	6*s*′ ${\;}^{3}D_{3}^{{}^{\circ}}—$ 6*p*″ ^1^D_2_
2873.667	1	34788.53	
2874.281	1	34781.10	
2874.550	1	34777.85	6*p*′ ^3^P_2_— $47_{1,2}^{{}^{\circ}}$
2875.738	2	34763.48	
2876.262	5	34757.15	5*p*^5^ ${\;}^{3}P_{0}^{{}^{\circ}}—$ 6*p*″ ^3^P_1_
2877.212	2*c*	34745.67	
2878.632	1500	34728.54	5*d* ${\;}^{3}D_{2}^{{}^{\circ}}—$ 4*f* ^3^F_3_
2879.021	30	34723.84	5*d* ${\;}^{3}D_{2}^{{}^{\circ}}—$ 6*p*″ ^3^D_2_
2879.172	8*h*	34722.02	
2883.019	60	34675.69	6*s* ${\;}^{5}S_{2}^{{}^{\circ}}—$ 6*p*′ ^3^P_2_
2883.440	10	34670.63	$\left\{ \begin{array}{l} 6p\prime {\;}^{3}D_{1}—\;8s\prime \;3D_{2}^{{}^{\circ}}\\ 6p\prime {\;}^{3}D_{2}—\;26_{3}^{{}^{\circ}} \end{array}\right.$
2883.996	1	34663.94	
2885.949	1	34640.49	
2886.446	10	34634.52	6*p*′ ^3^D_1_ — 8*s*′ ${\;}^{3}D_{1}^{{}^{\circ}}$
2886.961	3	34628.35	6*p* ^5^P_2_ — 6*d*′ $2_{3}^{{}^{\circ}}$
2888.024	1	34615.60	6*p*′ ^3^P_2_ — $44_{2,3}^{{}^{\circ}}$
2888.874	2	34605.42	6*p*′ ^3^D_2_ — $25_{1,2}^{{}^{\circ}}$
2890.137	1	34590.29	
2890.498	10	34585.97	5*d* ${\;}^{3}D_{2}^{{}^{\circ}}—$ 7*p* ^5^P_2_
2891.462	7	34574.44	5*d*″ ${\;}^{1}D_{2}^{{}^{\circ}}—$ 3_2,3_
2892.878	3	34557.52	6*p* ^5^P_3_ — 7*d* ${\;}^{3}D_{2}^{{}^{\circ}}$
2893.566	1*c*	34549.30	
2893.847	7	34545.95	5*p*^5^ ${\;}^{3}P_{0}^{{}^{\circ}}—$ 7*p* ^5^P_1_
2894.114	10	34542.76	6*p* ^3^P_1_ — 7*d* ${\;}^{3}D_{1}^{{}^{\circ}}$
2894.377	8	34539.62	6*p*′ ^3^F_2_ — $20_{1,2}^{{}^{\circ}}$
2895.354	3	34527.97	5*d*′ ${\;}^{3}F_{2}^{{}^{\circ}}—$ 7*p*′ ^3^D_2_
2895.498	15	34526.25	5*d*′ ${\;}^{3}G_{4}^{{}^{\circ}}—$ 5*f* ^3^F_4_
2896.813	10	34510.58	6*p*′ ^3^P_2_ — $43_{1,2}^{{}^{\circ}}$
2897.909	10	34497.53	
2900.253	15*c*	34469.65	5*d*′ ${\;}^{1}P_{1}^{{}^{\circ}}—$ 8_1,2_
2903.015	10	34436.86	5*p*^5^ ${\;}^{3}P_{0}^{{}^{\circ}}—$ 4*f* ^5^F_1_
2903.488	1	34431.25	6*p*′ ^3^F_3_ — $26_{3}^{{}^{\circ}}$
2906.004	2	34401.44	6*p*′ ^3^P_1_ — $47_{1,2}^{{}^{\circ}}$
2909.390	5*c*	34361.40	5*d*′ ${\;}^{3}D_{2}^{{}^{\circ}}—$ 6*p*″ ^1^D_2_
2910.567	8	34347.51	5*d*′ ${\;}^{3}P_{2}^{{}^{\circ}}—$ 5_2,3_
2910.906	1	34343.51	5*d* ${\;}^{3}D_{2}^{{}^{\circ}}—$ 4*f* ^5^F_1_
2914.458	20	34301.65	6*p*′ ^3^D_2_ — $24_{1,2}^{{}^{\circ}}$
2915.061	15*c*	34294.56	6*p*′ ^3^P_2_ — $42_{2}^{{}^{\circ}}$
2915.762	3*c*	34286.31	5*d*″ ${\;}^{3}D_{3}^{{}^{\circ}}—$ 3_2,3_
2915.881	10	34284.91	5*d*″ ${\;}^{3}D_{2}^{{}^{\circ}}—$ 4*f* ^5^F_2_
2916.146	4	34281.80	6*p*′ ^3^P_1_ — $46_{2}^{{}^{\circ}}$
2917.740	8	34263.07	6*p*′ ^3^P_0_ — $38_{1}^{{}^{\circ}}$
2917.911	2	34261.06	6*p*′ ^3^P_1_ — $45_{1,2}^{{}^{\circ}}$
2919.715	6*c*	34239.90	6*s* ${\;}^{3}S_{1}^{{}^{\circ}}—$ 6*p*′ ^1^D_2_
2920.122	10	34235.12	6*s* ${\;}^{5}S_{2}^{{}^{\circ}}—$ 6*p*′ ^3^D_3_
2921.134	6*c*	34223.26	6*p* ^5^P_2_ — 7*s*′ ${\;}^{3}D_{3}^{{}^{\circ}}$
2921.686	8*c*	34216.80	
2921.982	50	34213.33	6*p*′ ^3^D_3_ — $40_{3}^{{}^{\circ}}$
2925.087	80*c*	34177.02	5*p*^5^ ${\;}^{3}P_{2}^{{}^{\circ}}—$ 6*p*′ ^3^P_1_
2926.769	3*c*	34157.38	
2927.388	2*c*	34150.15	6*p*′ ^1^F_3_ — $36_{2}^{{}^{\circ}}$
2927.664	1*c*	34146.93	
2927.850	30	34144.76	
2928.758	4*c*	34134.18	6*p*′ ^3^P_1_ — $43_{1,2}^{{}^{\circ}}$
2929.112	60	34130.05	
2930.627	2	34112.41	6*p*′ ^3^D_2_ — $21_{1}^{{}^{\circ}}$
2931.720	200	34099.69	6*p* ^5^P_3_ — 7*d* ${\;}^{5}D_{4}^{{}^{\circ}}$
2932.911	10*c*	34085.85	6*s*″ ${\;}^{3}P_{1}^{{}^{\circ}}—$ 7*p*′ ^1^D_2_
2933.394	1	34080.24	6*p* ^5^P_1_ — 6*d*′ $1_{2}^{{}^{\circ}}$
2933.638	5	34077.40	6*p* ^5^P_1_ — 8*s* ${\;}^{3}S_{1}^{{}^{\circ}}$
2934.963	25	34062.02	6*p*′ ^3^F_3_ — $24_{1,2}^{{}^{\circ}}$
2936.954	40	34038.93	6*p* ^5^P_3_ — 7*d* ${\;}^{5}D_{3}^{{}^{\circ}}$
2938.155	6	34025.01	5*d*′ ${\;}^{3}G_{4}^{{}^{\circ}}—$ 5*f* ^5^F_5_
2940.471	40	33998.22	6*p*′ ^3^D_3_ — $39_{4}^{{}^{\circ}}$
2941.778	4	33983.11	5*d*′ ${\;}^{3}G_{4}^{{}^{\circ}}—$ 5*f* ^5^F_4_
2942.429	60	33975.59	
2942.923	10	33969.89	6*p* ^5^P_2_ — 8*s* ${\;}^{3}S_{1}^{{}^{\circ}}$
2943.192	15	33966.79	6*p* ^5^P_3_ — 7*d* ${\;}^{5}D_{2}^{{}^{\circ}}$
2943.267	1	33965.92	
2943.842	2	33959.29	5*d*′ ${\;}^{3}F_{2}^{{}^{\circ}}—$ 6*f* ^3^F_2_
2945.857	10*c*	33936.06	6*p*′ ^3^P_2_ — 12*d* ${\;}^{3}D_{3}^{{}^{\circ}}$
2946.081	8*c*	33933.48	6*s*″ ${\;}^{3}P_{1}^{{}^{\circ}}—$ 4*f*′ ^3^D_1_
2947.055	10	33922.26	6*p*′ ^1^F_3_ — $35_{2,3}^{{}^{\circ}}$
2947.927	40	33912.23	6*p*′ ^3^F_4_ — $39_{4}^{{}^{\circ}}$
2948.642	25	33904.01	5*d*′ ${\;}^{3}G_{3}^{{}^{\circ}}—$ 6*f* ^3^F_4_
2950.152	6	33886.66	5*d*′ ${\;}^{3}F_{2}^{{}^{\circ}}—$ 6*f* ^3^F_3_
2953.773	5	33845.12	5*d*′ ${\;}^{3}G_{3}^{{}^{\circ}}—$ 6*f* ^3^F_2_
2955.086	20*c*	33830.08	
2955.795	1*h*	33821.96	
2956.236	100*c*	33816.92	5*p*^5^ ${\;}^{1}P_{1}^{{}^{\circ}}—$ 6*p*″ ^3^P_2_
2957.008	5*c*	33808.09	6*s*″ ${\;}^{3}P_{1}^{{}^{\circ}}—$ 7*p*′ ^3^F_2_
2957.658	200*c*	33800.66	5*p*^5^ ${\;}^{3}P_{2}^{{}^{\circ}}—$ 6*p*′ ^3^P_2_
2959.796	15	33776.25	
2960.113	1	33772.63	6*p*′ ^3^P_2_ — $40_{3}^{{}^{\circ}}$
2962.632	10*c*	33743.92	6*s*′ ${\;}^{1}D_{2}^{{}^{\circ}}—$ 6*p*″ ^1^D_2_
2962.819	1	33741.78	5*d*′ ${\;}^{3}D_{2}^{{}^{\circ}}—$ 6*p*″ ^1^P_1_
2967.350	5	33690.27	5*d*′ ${\;}^{3}D_{1}^{{}^{\circ}}—$ 4*f*′ ^3^P_0_
2969.413	25	33666.86	6*p*′ ^1^F_3_ — $33_{3}^{{}^{\circ}}$
2970.694	7	33652.34	
2970.819	30*c*	33650.93	5*d*′ ${\;}^{1}G_{4}^{{}^{\circ}}—$ 4*f*′ ^3^H_5_
2971.895	2	33638.74	
2973.766	40	33617.58	6*p* ^5^P_1_ — 8*s* ${\;}^{5}S_{2}^{{}^{\circ}}$
2974.880	20*c*	33604.99	5*d*″ ${\;}^{1}D_{2}^{{}^{\circ}}—$ 2_2,3_
2977.650	4	33573.66	6*p* ^3^P_2_ — 7*d* ${\;}^{3}D_{1}^{{}^{\circ}}$
2978.287	4*c*	33566.55	5*d*″ ${\;}^{1}D_{2}^{{}^{\circ}}—$ 8*f* ^5^F_3_
2979.044	25*c*	33558.02	
2979.487	2	33553.03	6*p* ^5^P_3_ — 6*d*′ $2_{3}^{{}^{\circ}}$
2980.322	7*c*	33543.63	6*p*′ ^1^P_1_ — $31_{0}^{{}^{\circ}}$
2981.162	2*c*	33534.18	
2983.312	50	33510.02	6*p* ^5^P_2_ — 8*s* ${\;}^{5}S_{2}^{{}^{\circ}}$
2986.011	4	33479.73	
2987.789	6	33459.80	5*d*′ ${\;}^{3}P_{2}^{{}^{\circ}}—$ 3_2,3_
2990.484	20*c*	33429.65	6*p*′ ^1^P_1_— $30_{2}^{{}^{\circ}}$
2992.391	2*c*	33408.35	
2993.866	1000	33391.89	5*d* ${\;}^{3}D_{1}^{{}^{\circ}}—$ 4*f* ^3^F_2_
2994.991	20	33379.35	6*p*′ ^3^F_2_— 8*s*′ ${\;}^{3}D_{2}^{{}^{\circ}}$
2996.734	100*c*	33359.94	5*p*^5^ ${\;}^{3}P_{2}^{{}^{\circ}}—$ 6*p*′ ^3^D_3_
2997.189	25*c*	33354.87	5*d* ${\;}^{5}D_{3}^{{}^{\circ}}—$ 6*p*′ ^1^D_2_
2998.229	10*d*	33343.30	6*p*′ ^3^F_2_— 8*s*′ ${\;}^{3}D_{1}^{{}^{\circ}}$
2999.498	20*c*	33329.20	6*p*′ ^1^P_1_— 10*d* ${\;}^{3}D_{2}^{{}^{\circ}}$
3000.694	40	33315.91	6*p* ^3^P_1_— 7*d* ${\;}^{3}D_{2}^{{}^{\circ}}$
3004.858	8	33269.75	6*p* ^3^P_1_— 6*d*′ $5_{1}^{{}^{\circ}}$
3007.318	.15*c*	33242.53	
3010.480	7*c*	33207.62	6*p*′ ^1^P_1_— $29_{1}^{{}^{\circ}}$
3012.712	20	33183.02	6*p* ^3^P_0_— 7*d* ${\;}^{3}D_{1}^{{}^{\circ}}$
3018.042	30*c*	33124.42	5*s* ${\;}^{5}S_{2}^{{}^{\circ}}—$ 6*p*′ ^1^F_3_
3018.249	20*c*	33122.14	6*s*′ ${\;}^{3}D_{3}^{{}^{\circ}}—$ 6*p*″ ^3^P_2_
3018.826	15*c*	33115.81	6*s*′ ${\;}^{3}P_{3}^{{}^{\circ}}—$ 6*p*″ ^3^D_1_
3020.751	35*c*	33094.71	5*d*′ ${\;}^{3}D_{1}^{{}^{\circ}}—$ 4*f*′ ^3^P_2_
3020.950	7*d*	33092.53	5*d*″ ${\;}^{1}D_{2}^{{}^{\circ}}—$ 1_2,3_
3021.214	100	33089.64	5*d* ${\;}^{3}D_{1}^{{}^{\circ}}—$ 6*p*″ ^3^D_2_
3023.211	1*h*	33067.78	5*d*′ ${\;}^{1}G_{4}^{{}^{\circ}}—$ 4*f*′ ^3^D_3_
3024.948	15*c*	33048.80	5*d*′ ${\;}^{3}F_{2}^{{}^{\circ}}—$ 4*f*′ ^1^P_1_
3031.204	30*c*	32980.59	5*d*′ ${\;}^{3}F_{2}^{{}^{\circ}}—$ 4*f*′ ^1^D_2_
3032.039	80*c*	3297.1.51	6*p* ^3^P_2_— 7*d* ${\;}^{3}D_{3}^{{}^{\circ}}$
3033.856	35	32951.76	5*d* ${\;}^{3}D_{1}^{{}^{\circ}}—$ 7*p* ^5^P_2_
3035.807	40	32930.59	5*d*′ ${\;}^{3}P_{0}^{{}^{\circ}}—$ 6*p*″ ^1^P_1_
3036.973	4*c*	32917.94	5*d* ${\;}^{5}D_{2}^{{}^{\circ}}—$ 6*p*′ ^1^D_2_
3040.888	15*c*	32875.56	5*d*′ ${\;}^{3}F_{2}^{{}^{\circ}}—$ 4*f*′ ^1^F_3_
3041.207	1*c*	32872.12	5*d*′ ${\;}^{3}F_{2}^{{}^{\circ}}—$ 7*p*′ ^3^F_3_
3041.735	15*c*	32866.41	5*d*′ ${\;}^{3}G_{3}^{{}^{\circ}}—$ 4*f*′ ^1^D_2_
3042.243	3*d*	32860.92	5*d*′ ${\;}^{1}D_{2}^{{}^{\circ}}—$ 6*f* ^3^F_3_
3043.530	8*d*	32847.03	
3045.391	30	32826.96	5*d*′ ${\;}^{3}D_{1}^{{}^{\circ}}—$ 7*p*′ ^3^P_2_
3049.691	80*c*	32780.67	5*s*′ ${\;}^{3}D_{3}^{{}^{\circ}}—$ 6*p*″ ^3^D_3_
3051.188	2	32764.59	6*p*′ ^1^P_1_— 11*s* ${\;}^{5}S_{2}^{{}^{\circ}}$
3051.792	40*c*	32758.11	5*d*′ ${\;}^{3}G_{3}^{{}^{\circ}}—$ 7*p*′ ^3^F_3_
3053.072	3	32744.37	5*d*′ ${\;}^{3}S_{1}^{{}^{\circ}}—$ 7*p*′ ^3^D_2_
3053.177	25	32743.25	5*d*′ ${\;}^{3}F_{2}^{{}^{\circ}}—$ 7*p*′ ^1^P_1_
3055.365	50	32719.80	6*p*′ ^3^D_3_— $34_{2}^{{}^{\circ}}$
3056.005	90*c*	32712.95	6*s*′ ${\;}^{3}D_{2}^{{}^{\circ}}—$ 7*p* ^3^P_2_
3059.322	50	32677.48	5*d*′ ${\;}^{3}F_{2}^{{}^{\circ}}—$ 7*p*′ ^1^D_2_
3061.716	20	32651.93	5*d*′ ${\;}^{3}F_{2}^{{}^{\circ}}—$ 7*p*′ ^1^F_3_
3064.603	2	32621.17	
3065.902	60*c*	32607.35	5*p*^5^ ${\;}^{1}P_{1}^{{}^{\circ}}—$ 6*p*″ ^3^S_1_
3067.346	1	32592.00	
3070.042	35	32563.38	5*d*′ ${\;}^{3}G_{3}^{{}^{\circ}}—$ 7*p*′ ^1^D_2_
3070.705	30*c*	32556.35	6*p*′ ^3^D_3_— $33_{3}^{{}^{\circ}}$
3071.046	2	32552.74	
3072.456	80	32537.80	5*d*′ ${\;}^{3}G_{3}^{{}^{\circ}}—$ 7*p*′ ^1^F_3_
3073.658	7*c*	32525.07	5*d*′ ${\;}^{3}F_{2}^{{}^{\circ}}—$ 4*f*′ ^3^D_1_
3074.132	3	32520.06	
3074.255	8	32518.76	5*d* ${\;}^{3}D_{3}^{{}^{\circ}}—$ 4*f* ^3^F_2_
3075.678	4	32503.71	
3076.210	20	32498.09	6*p*′ ^3^D_2_— 8*s*′ ${\;}^{3}D_{2}^{{}^{\circ}}$
3077.708	40*c*	32482.28	5*d*′ ${\;}^{3}G_{3}^{{}^{\circ}}—$ 7*p*′ ^3^F_4_
3078.754	5000	32471.24	5*d* ${\;}^{3}D_{3}^{{}^{\circ}}—$ 4*f* ^3^F_4_
3079.641	20	32461.89	6*p*′ ^3^D_2_— 8*s*′ ${\;}^{3}D_{1}^{{}^{\circ}}$
3082.229	60	32434.63	6*p* ^5^P_3_— 8*s* ${\;}^{5}S_{2}^{{}^{\circ}}$
3083.315	4	32423.21	
3084.278	6*c*	32413.09	6*p*′ ^3^F_3_— 9*d* ${\;}^{3}D_{2}^{{}^{\circ}}$
3085.509	60*c*	32400.16	
3086.446	3	32390.32	
3086.989	4	32384.62	6*p′* ^3^P_0_— $29_{1}^{{}^{\circ}}$
3089.652	20*d*	32356.71	6*s′* ${\;}^{3}D_{1}^{{}^{\circ}}—$ 7*p* ^3^P_0_
3090.476	10*c*	32348.08	5*d′* ${\;}^{3}D_{2}^{{}^{\circ}}—$ 6*p″* ^3^D_3_
3090.609	25	32346.69	6*p* ^3^P_2_— 7*d* ${\;}^{3}D_{2}^{{}^{\circ}}$
3092.698	15*c*	32324.84	
3094.035	1	32310.88	
3096.384	15*c*	32286.36	
3096.459	10	32285.58	5*d′* ${\;}^{3}G_{3}^{{}^{\circ}}—$ 7*p′* ^3^F_2_
3097.075	1*h*	32279.16	6*p′* ^3^P_2_— $34_{2}^{{}^{\circ}}$
3099.064	35	32258.45	6*p′* ^3^F_3_— 8*s′* ${\;}^{3}D_{2}^{{}^{\circ}}$
3099.925	60*c*	32249.49	5*p*^5^ ${\;}^{3}P_{2}^{{}^{\circ}}—$ 6*p′* ^1^F_3_
3101.067	3*c*	32237.61	
3102.663	60	32221.03	5*d* ${\;}^{3}D_{3}^{{}^{\circ}}—$ 4*f* ^3^F_3_
3104.171	10	32205.38	5*d′* ${\;}^{3}P_{1}^{{}^{\circ}}—$ 12_1,2_
3106.665	40	32179.52	5*d′* ${\;}^{3}F_{2}^{{}^{\circ}}—$ 4*f′* ^3^G_3_
3112.838	30*c*	32115.71	6*p′* ^3^P_2_— $33_{3}^{{}^{\circ}}$
3113.026	10	32113.77	5*d′* ${\;}^{3}D_{1}^{{}^{\circ}}—$ 7*p′* ^3^P_1_
3116.441	8	32078.58	5*d* ${\;}^{3}D_{3}^{{}^{\circ}}—$ 7*p* ^5^P_2_
3117.071	30*c*	32072.10	6*s′* ${\;}^{1}D_{2}^{{}^{\circ}}—$ 6*p″* ^3^P_2_
3117.718	40*c*	32065.44	5*d′* ${\;}^{3}G_{3}^{{}^{\circ}}—$ 4*f′* ^3^G_3_
3119.878	5	32043.24	
3121.648	15*c*	32025.08	6*p′* ^1^P_1_— $20_{1,2}^{{}^{\circ}}$
3125.976	50	31980.74	5*d′* ${\;}^{3}D_{1}^{{}^{\circ}}—$ 4*f′* ^3^F_2_
3128.507	35*c*	31954.87	5*d′* ${\;}^{1}D_{2}^{{}^{\circ}}—$ 4*f′* ^1^D_2_
3128.638	10	31953.53	6*p′* ^3^P_0_—11*s* ${\;}^{3}S_{1}^{{}^{\circ}}$
3132.910	25*c*	31909.96	6*p* ^3^P_0_— 6*d′* $5_{1}^{{}^{\circ}}$
3133.397	50*c*	31905.00	5*p*^5^ ${\;}^{3}P_{2}^{{}^{\circ}}—$ 6*p′* ^1^P_1_
3133.618	10*c*	31902.75	6*p′* ^3^P_1_— $34_{2}^{{}^{\circ}}$
3136.473	100*d*	31873.71	5*d′* ${\;}^{3}G_{3}^{{}^{\circ}}—$ 4*f′* ^3^F_4_
3137.604	60	31862.22	5*d′* ${\;}^{3}D_{1}^{{}^{\circ}}—$ 8*p* ^3^P_1_
3138.830	90*c*	31849.78	5*d′* ${\;}^{1}D_{2}^{{}^{\circ}}—$ 4*f′* ^1^F_3_
3140.946	8	31828.32	6*p* ^3^P_2_— 7*d* ${\;}^{5}D_{3}^{{}^{\circ}}$
3144.212	1	31795.26	
3144.997	1	31787.33	
3145.346	2	31783.80	
3148.089	8	31756.10	6*p* ^3^P_2_— 7*d* ${\;}^{5}D_{2}^{{}^{\circ}}$
3149.636	8	31740.51	
3151.915	2	31717.56	5*d* ${\;}^{1}D_{2}^{{}^{\circ}}—$ 7*p′* ^1^P_1_
3153.078	5*c*	31705.86	5*d* ${\;}^{3}D_{3}^{{}^{\circ}}—$ 4*f* ^5^F_3_
3155.573	1	31680.79	6*p′* ^1^F_3_— $20_{1,2}^{{}^{\circ}}$
3155.864	10	31677.87	5*d* ${\;}^{3}D_{3}^{{}^{\circ}}—$ 4*f* ^5^F_4_
3157.337	3	31663.09	5*d′* ${\;}^{3}D_{1}^{{}^{\circ}}—$ 7*p′* ^3^D_1_
3158.080	20	31655.64	6*p* ^3^P_1_— 6*d′* $1_{2}^{{}^{\circ}}$
3158.357	20	31652.87	6*p* ^3^P_1_— 8*s* ${\;}^{3}S_{1}^{{}^{\circ}}$
3158.466	40	31651.78	5*d′* ${\;}^{1}D_{2}^{{}^{\circ}}—$ 7*p′* ^1^D_2_
3159.175	1	31644.67	
3161.027	200	31626.13	5*d′* ${\;}^{1}D_{2}^{{}^{\circ}}—$ 7*p′* ^1^F_3_
3163.935	40*c*	31597.07	5*d′* ${\;}^{3}D_{3}^{{}^{\circ}}—$ 6*p″* ^3^P_2_
3169.882	15*c*	31537.79	6*s″* ${\;}^{3}P_{1}^{{}^{\circ}}—$ 4*f′* ^3^P_2_
3173.509	3*c*	31501.75	
3173.752	5*c*	31499.34	5*d′* ${\;}^{1}D_{2}^{{}^{\circ}}—$ 4*f′* ^3^D_1_
3175.066	1000	31486.30	5*p′* ${\;}^{3}P_{1}^{{}^{\circ}}—$ 6*p′* ^3^P_2_
3176.630	15*c*	31470.80	6*s′* ${\;}^{3}D_{2}^{{}^{\circ}}—$ 6*p″* ^3^P_1_
3177.828	10	31458.94	6*s″* ${\;}^{3}P_{0}^{{}^{\circ}}—$ 7*p′* ^3^P_1_
3178.064	1	31456.60	6*p′* ^3^D_1_— 8*d* ${\;}^{3}D_{2}^{{}^{\circ}}$
3180.575	10*c*	31431.76	5*d″* ${\;}^{3}P_{2}^{{}^{\circ}}—$_ _ 8_1,2_
3182.602	2	31411.75	
3182.709	10*d*	31410.69	6*s′* ${\;}^{3}D_{1}^{{}^{\circ}}—$ 7*p* ^3^P_1_
3184.489	4*c*	31393.13	6*s′* ${\;}^{3}D_{2}^{{}^{\circ}}—$ 7*p* ^5^P_2_
3186.440	20	31873.91	5*d′* ${\;}^{1}D_{2}^{{}^{\circ}}—$ 7*p′* ^3^F_2_
3189.680	20	31342.05	6*p* ^3^P_2_— 6*d′* $2_{3}^{{}^{\circ}}$
3194.682	35	31292.98	5*d′* ${\;}^{3}P_{1}^{{}^{\circ}}—$_ _ 8_1,2_
3195.610	6*c*	31283.89	6*p′* ^1^D_2_— $46_{2}^{{}^{\circ}}$
3197.074	40*d*	31269.56	5*d″* ${\;}^{3}P_{2}^{{}^{\circ}}—$_ _ 7_2,3_
3197.514	2*c*	31265.26	5*d′* ${\;}^{3}S_{1}^{{}^{\circ}}—$ 4*f′* ^1^P_1_
3197.711	2*c*	31268.33	6*p′* ^1^D_2_— $45_{1,}^{{}^{\circ}}{\;}_{2}$
3198.069	2*c*	31259.83	
3199.891	60	31242.04	6*s* ${\;}^{3}S_{1}^{{}^{\circ}}—$ 6*p′* ^3^P_1_
3201.547	3*c*	31225.88	5*p*^5^ ${\;}^{1}P_{1}^{{}^{\circ}}—$ 7*p* ^3^P_0_
3204.517	5*c*	31196.94	5*d′* ${\;}^{3}S_{1}^{{}^{\circ}}—$ 4*f′* ^1^D_2_
3204.894	2	31193.27	6*p* ${\;}^{3}P_{1}^{{}^{\circ}}—$ 8*s* ${\;}^{5}S_{2}^{{}^{\circ}}$
3208.946	25	31153.88	5*d′* ${\;}^{1}D_{2}^{{}^{\circ}}—$ 4*f*′ ^3^G_3_
3209.664	30*c*	31146.91	6*s* ${\;}^{5}S_{2}^{{}^{\circ}}—$ 6*p′* ^3^D_2_
3221.493	8	31032.55	5*d′* ${\;}^{3}S_{1}^{{}^{\circ}}—$7*p′* ^3^P_0_
3229.081	4	80959.63	5*d′* ${\;}^{3}S_{1}^{{}^{\circ}}—$ 7*p′* ^1^P_1_
3231.433	2*c*	309:37.09	6*p* ^3^P_2_— 7*s′* ${\;}^{3}D_{3}^{{}^{\circ}}$
8235.951	5	30893.90	5*d′* ${\;}^{3}S_{1}^{{}^{\circ}}—$ 7*p′* ^1^D_2_
3238.909	25*c*	30865.69	6*s* ${\;}^{3}S_{1}^{{}^{\circ}}—$ 6*p′* ^3^P_2_
3239.245	20*c*	30862.48	6*s′* ${\;}^{1}D_{2}^{{}^{\circ}}—$ 6*p*″ ^3^S_1_
3252.008	3	30741.36	5*d′* ${\;}^{3}S_{1}^{{}^{\circ}}—$ 4*f*′ ^3^D_1_
3256.460	100*c*	30699.84	6*s*′ ${\;}^{3}D_{1}^{{}^{\circ}}—$ 4*f* ^3^F_2_
3257.814	2	30686.58	
3258.125	20	30683.65	6*p* ^3^P_2_— 8*s* ${\;}^{3}S_{1}^{{}^{\circ}}$
3265.309	1	30616.15	5*d′* ${\;}^{3}S_{1}^{{}^{\circ}}—$ 7*p′* ^3^F_2_
3267.320	5*c*	30597.30	6*s″* ${\;}^{3}P_{1}^{{}^{\circ}}—$ 8*p* ^3^P_2_
3270.440	4	30568.12	
3271.534	1	30557.89	
3276.536	30*c*	30511.24	5*p*^5^ ${\;}^{3}P_{2}^{{}^{\circ}}—$ 6*p′* ^3^F_3_
3278.418	1*d*	30493.73	6*p′* ^3^D_1_— 6*d′* $15_{1}^{{}^{\circ}}$
3279.256	2*c*	30485.94	
3288.860	100*c*	30396.92	6*s*′ ${\;}^{3}D_{1}^{{}^{\circ}}—$ 6*p″* ^3^D_2_
3296.368	3*c*	30327.68	5*d′* ${\;}^{3}P_{2}^{{}^{\circ}}—$ 7*f* ^3^F_3_
3300.133	10	30293.09	6*p* ^3^P_0_— 8*s* ${\;}^{3}S_{1}^{{}^{\circ}}$
3301.538	20*c*	30280.20	5*p*^5^ ${\;}^{1}P_{1}^{{}^{\circ}}—$ 7*p* ^3^P_1_
3302.457	150*c*	30271.77	5*p*^5^ ${\;}^{3}P_{2}^{{}^{\circ}}—$ 6*p′* ^3^D_2_
3303.841	15	30259.09	6*s′* ${\;}^{3}D_{1}^{{}^{\circ}}—$ 7*p* ^5^P_2_
3307.70l	10	30223.78	6*p* ^3^P_2_— 8*s* ${\;}^{5}S_{2}^{{}^{\circ}}$
3309.502	1	30207.33	
3312.374	10*d*	30181.14	6*s*′ ${\;}^{3}D_{1}^{{}^{\circ}}—$ 6*p″* ^3^P_0_
3313.825	1	30167.93	
3314.478	3	30161.98	
3315.326	2*c*	30154.27	
3318.088	40	30129.17	$\left\{ \begin{array}{l} 5d^{\prime }{\;}^{3}F_{2}^{{}^{\circ}}—\;4f{}^{\circ}{\;}^{3}P_{2}\\ 6p^{\prime }{\;}^{3}P_{2}—\;20_{1,2}^{{}^{\circ}} \end{array}\right.$
3319.210	2	30118.98	6*p′* ^3^D_1_— 9*s* ${\;}^{3}S_{1}^{{}^{\circ}}$
3321.099	1	30101.85	
3321.641	1	30096.94	
3323.700	2	30078.30	6*s*″ ${\;}^{1}P_{1}^{{}^{\circ}}—$ 7*p′* ^3^D_2_
3326.405	50*c*	30053.84	5*d′* ${\;}^{3}F_{4}^{{}^{\circ}}—$ 6*p″* ^3^D_3_
3327.256	1*c*	30046.15	
3330.708	2*c*	30015.01	5*d′* ${\;}^{3}G_{3}^{{}^{\circ}}—$ 4*f*′ ^3^P_2_
3331.585	6	30007.11	5*d″* ${\;}^{3}P_{2}^{{}^{\circ}}—$_ _ 3_2,3_
3331.754	8	30005.59	6*p′* ^3^P_0_*—* 8*s′* ${\;}^{3}D_{1}^{{}^{\circ}}$
3334.536	2*c*	29980.56	5*d* ${\;}^{5}D_{3}^{{}^{\circ}}—$ 6*p′* ^3^P_2_
3340.351	3*c*	29928.37	6*s″* ${\;}^{3}P_{2}^{{}^{\circ}}—$ 4*f*′ ^1^F_3_
3341.289	100	29919.97	5*d* ${\;}^{5}D_{2}^{{}^{\circ}}—$ 6*p′* ^3^P_1_
3345.342	25*c*	29883.72	5*d″* ${\;}^{3}D_{2}^{{}^{\circ}}—$_ _ 7_2,3_
3345.607	3*c*	29881.35	5*d′* ${\;}^{3}F_{2}^{{}^{\circ}}—$ 4*f*′ ^3^P_1_
3346.219	2*c*	29875.89	
3347.857	40*c*	29861.27	5*d′* ${\;}^{3}F_{2}^{{}^{\circ}}—$ 7*p′* ^3^P_2_
3349.286	20	2984.53	5*d*′ ${\;}^{3}F_{2}^{{}^{\circ}}—$ 7*p*′ ^3^D_3_
3351.394	30	29829.76	5*d*′ ${\;}^{3}F_{3}^{{}^{\circ}}—$ 6*p*″ ^3^P_2_
3352.823	10*c*	29817.04	5*d*′ ${\;}^{3}D_{1}^{{}^{\circ}}—$ 6*p*″ ^1^S_0_
3352.928	2	29816.11	6*p*′ ${\;}^{1}D_{2}^{{}^{\circ}}—$ $38_{1}^{{}^{\circ}}$
3355.531	300	29792.98	6*s* ${\;}^{3}S_{1}^{{}^{\circ}}—$ 6*p*′ ^3^P_0_
3359.961	150*c*	29753.70	6*s*′ ${\;}^{3}D_{3}^{{}^{\circ}}—$ 7*p* ^3^P_2_
3360.699	8*c*	29747.17	5*d*′ ${\;}^{3}G_{3}^{{}^{\circ}}—$ 7*p*′ ^3^P_2_
3362.137	7	29734.44	5*d*′ ${\;}^{3}G_{3}^{{}^{\circ}}—$ 7*p*′ ^3^D_3_
3362.598	20*c*	29730.37	6*s*″ ${\;}^{3}P_{2}^{{}^{\circ}}—$ 7*p*′ ^1^D_2_
3363.367	2	29723.57	
3365.500	20*c*	29704.73	6*s*″ ${\;}^{3}P_{2}^{{}^{\circ}}—$ 7*p*′ ^1^F_3_
3366.575	7*d*	29695.25	
3374.457	100*c*	29625.89	5*d* ${\;}^{5}D_{3}^{{}^{\circ}}—$ 6*p*′ ^3^F_4_
3378.475	6*c*	29590.66	5*p*^5^ ${\;}^{3}P_{1}^{{}^{\circ}}—$ 6*p*′ ^1^P_1_
3380.983	30*c*	29568.71	5*p*^5^ ${\;}^{1}P_{1}^{{}^{\circ}}—$ 4*f* ^3^F_2_
3382.711	2	29553.60	
3383.858	80*c*	29543.58	5*d* ${\;}^{5}D_{2}^{{}^{\circ}}—$ 6*p*′ ^3^P_2_
3386.377	4*c*	29521.61	5*d*′ ${\;}^{1}S_{0}^{{}^{\circ}}—$ 4*f*′ ^1^P_1_
3390.214	15*c*	29488.20	5*d*′ ${\;}^{3}F_{3}^{{}^{\circ}}—$ 6*p*″ ^3^D_3_
3391.360	7*c*	29478.23	7*s* ${\;}^{3}S_{1}^{{}^{\circ}}—$ 8_1,2_
3394.192	7*c*	29453.64	
3399.537	8	29407.33	
3400.316	5	29400.59	
3401.500	50*c*	29390.36	5*p*^5^ ${\;}^{3}P_{2}^{{}^{\circ}}—$ 6*p*′ ^3^F_2_
3402.587	4*c*	29380.97	
3409.138	2*c*	29324.52	
3409.692	7*c*	29319.75	
3409.938	30*c*	29317.64	5*d* ${\;}^{5}D_{4}^{{}^{\circ}}—$ 6*p*′ ^3^F_4_
3411.173	1	29307.02	
3415.911	30*c*	29266.37	5*p*^5^ ${\;}^{1}P_{1}^{{}^{\circ}}—$ 6*p*″ ^3^D_2_
3416.759	5	29259.11	
3417.606	5	29251.86	
3421.805	7	29215.96	5*d*′ ${\;}^{1}S_{0}^{{}^{\circ}}—$ 7*p*′ ^1^P_1_
3421.987	3	29214.41	
3422.948	20*c*	29206.21	5*p*^5^ ${\;}^{1}P_{1}^{{}^{\circ}}—$ 6*p*″ ^3^P_1_
3424.993	250*c*	29188.77	5*d*′ ${\;}^{3}F_{2}^{{}^{\circ}}—$ 8*p* ^3^P_2_
3426.461	20	29176.26	5*d*′ ${\;}^{3}G_{3}^{{}^{\circ}}—$4*f*′ ^3^H_4_
3429.784	7*d*	29148.00	5*d*′ ${\;}^{3}F_{2}^{{}^{\circ}}—$ 7*p*′ ^3^P_1_
3432.080	25*c*	29128.50	5*p*^5^ ${\;}^{1}P_{1}^{{}^{\circ}}—$ 7*p* ^5^P_2_
3433.277	4*c*	29118.34	6*s*″ ${\;}^{3}P_{1}^{{}^{\circ}}—$ 5*f* ^5^F_2_
3435.093	80*c*	29102.95	5*d* ${\;}^{5}D_{2}^{{}^{\circ}}—$ 6*p*′ ^3^D_3_
3436.712	3	29089.24	
3438.434	20*w*	29074.67	5*d*′ ${\;}^{3}G_{3}^{{}^{\circ}}—$ 8*p* ^3^P_2_
3438.792	3	29071.65	
3440.510	2	29057.13	
3441.290	25*c*	29050.54	5*p*^5^ ${\;}^{1}P_{1}^{{}^{\circ}}—$ 6*p*″ ^3^P_0_
3442.819	7	29037.64	5*d*″ ${\;}^{3}P_{2}^{{}^{\circ}}—$ 2_2,3_
3444.433	1	29024.04	5*d*″ ${\;}^{3}P_{3}^{{}^{\circ}}—$ 10_2,3_
3446.365	1	29007.77	
3447.683	35*c*	28996.68	6*p*′ ^1^D_2_— $35_{2,}^{{}^{\circ}}{\;}_{3}$
3447.872	10*c*	28995.09	5*p*^5^ ${\;}^{1}P_{1}^{{}^{\circ}}—$ 7*p* ^5^P_1_
3450.764	8*c*	28970.79	
3454.191	4	28942.05	
3455.886	1	28927.85	
3458.654	20	28904.70	6*p*′ ^1^D_2_— $34_{2}^{{}^{\circ}}$
3459.108	10	28900.91	5*d*′ ${\;}^{3}G_{3}^{{}^{\circ}}—$ 4*f*′ ^3^F_2_
3459.626	3	28896.58	5*d*′ ${\;}^{3}F_{2}^{{}^{\circ}}—$ 8*p* ^3^P_1_
3461.812	1	28878.34	
3462.097	1	28875.96	
3462.332	2*c*	28874.00	6*s*′ ${\;}^{3}D_{3}^{{}^{\circ}}—$ 4*f* ^3^F_2_
3464.540	·4*c*	255.60	5*d*′ ${\;}^{1}D_{2}^{{}^{\circ}}—$ 4*f*′ ^3^P_1_
3466.953	10	28835.51	5*d*′ ${\;}^{1}D_{2}^{{}^{\circ}}—$ 7*p*′ ^3^P_2_
3467.261	5*c*	28832.95	6*s*′ ${\;}^{3}D_{3}^{{}^{\circ}}—$ 7*p* ^5^P_3_
3468.046	30*c*	28826.43	6*s*′ ${\;}^{3}D_{3}^{{}^{\circ}}—$ 4*f* ^3^F_4_
3468.486	20	28822.77	5*d*′ ${\;}^{1}D_{2}^{{}^{\circ}}—$ 7*p*′ ^3^D_3_
3469.342	1	28815.66	
3470.131	6	28809.11	
3478.279	3*c*	28741.62	
3481.569	1	28714.46	
3482.917	1	28703.35	6*s*′ ${\;}^{1}D_{2}^{{}^{\circ}}—$ 7*p* ^3^P_2_
3483.706	5	28696.85	
3483894	60*c*	28695.30	6*s*′ ${\;}^{3}D_{1}^{{}^{\circ}}—$ 6*p*″ ^3^D_1_
3488.509	15	28657.34	5*d*′ ${\;}^{3}F_{2}^{{}^{\circ}}—$ 4*f*′ ^3^D_3_
3489.100	2	28652.49	
3489.561	4	28648.70	
3490.488	1	28641.09	
3491.273	1	28634.65	
3492.916	4	28621.18	5*d*″ ${\;}^{3}D_{2}^{{}^{\circ}}—$ 3_2,3_
3495.624	1*c*	28599.01	6*s*″ ${\;}^{1}P_{1}^{{}^{\circ}}—$4*f*′ ^1^P_1_
3496.408	5	28592.60	6*p*′ ${\;}^{3}P_{1}^{{}^{\circ}}—$ 8*s*′ ${\;}^{3}D_{2}^{{}^{\circ}}$
3497.406	300*c*	28584.44	5*d* ${\;}^{3}P_{2}^{{}^{\circ}}—$ 6*p*′ ^1^D_2_
3498.372	2	28576.55	
3498.500	1	28575.50	
3498.985	25*c*	28571.54	6*s*′ ${\;}^{3}D_{3}^{{}^{\circ}}—$ 6*p*″ ^3^D_2_
3500.824	2	28556.53	6*p*′ ${\;}^{3}P_{1}^{{}^{\circ}}—$ 8*s*′ ${\;}^{3}D_{1}^{{}^{\circ}}$
3502.457	2	28543.22	5*d*′ ${\;}^{3}G_{3}^{{}^{\circ}}—$ 4*f*′ ^3^D_3_
3503.459	35*c*	28535.06	6*s*′ ${\;}^{1}D_{2}^{{}^{\circ}}—$ 7*p* ^3^P_1_
3503.996	30*c*	28530.68	6*s*″ ${\;}^{1}P_{1}^{{}^{\circ}}—$ 4*f*′ ^1^D_2_
3504.729	1*c*	28524.72	
3510.630	10	28476.77	5*d*′ ${\;}^{1}D_{2}^{{}^{\circ}}—$ 4*f*′ ^3^D_2_
3511.357	20	28470.88	5*d*′ ${\;}^{3}G_{3}^{{}^{\circ}}—$ 4*f*′ ^3^F_3_
3512.177	100	28464.23	5*d*′ ${\;}^{3}G_{3}^{{}^{\circ}}—$ 4*f*′ ^3^G_4_
3513.304	2	28455.10	
3513.971	2	28449.70	
3514.319	1	28446.88	7*s* ${\;}^{5}S_{2}^{{}^{\circ}}—$8*f* ^3^F_3_
3514.757	2	28443.34	
3515.021	15	28441.20	5*d*′ ${\;}^{3}D_{2}^{{}^{\circ}}—$ 4*f* ^3^F_2_
3515.661	3	28436.02	
3516.504	50*c*	28429.20	5*d* ${\;}^{5}D_{3}^{{}^{\circ}}—$ 6*p*′ ^1^F_3_
3517.822	3*h*	28418.55	5*d*′ ${\;}^{3}G_{3}^{{}^{\circ}}—$ 8*p* ^5^P_2_?
3518.688	1	28411.56	
3519.105	3	28408.19	
3520.089	5*c*	28400.25	5*d*′ ${\;}^{3}D_{2}^{{}^{\circ}}—$ 7*p* ^5^P_3_
3522.007	2	28384.79	
3523.646	2	28371.58	
3523.886	1	28369.65	
3524.095	2	28367.97	
3524.292	15	28366.38	6*s*″ ${\;}^{1}P_{1}^{{}^{\circ}}—$ 7*p*′ ^3^P_0_
3525.403	2	28357.44	6*p*′ ${\;}^{3}D_{1}^{{}^{\circ}}—$ 6*d*′ $11_{0}^{{}^{\circ}}$
3525.798	1	28354.27	
3526.223	60*d*	28350.85	5*d* ${\;}^{5}D_{1}^{{}^{\circ}}—$6*p*′ ^3^P_1_
3526.904	500	28345.38	5*d*′ ${\;}^{3}S_{1}^{{}^{\circ}}—$ 4*f*′ ^3^P_2_
3527.385	4	28341.51	5*d*′ ${\;}^{3}P_{0}^{{}^{\circ}}—$ 7*p* ^3^P_1_
3528.062	2	28336.07	6*p*′ ^3^F_2_—6*d*′ $14_{3}^{{}^{\circ}}$
3528.574.	2	28331.96	
3528.810	1	28330.07	
3529.316	2*h*	28326.00	
3529.540	1	28324.21	
3531.331	5*h*	28309.84	6*p*′ ^3^D_1_— 6*d*′ $10_{2}^{{}^{\circ}}$
3533.379	60	28293.43	6*s*″ ${\;}^{1}P_{1}^{{}^{\circ}}—$ 7*p*′ ^1^P_1_
3533.648	10	28291.28	5*d*″ ${\;}^{3}D_{1}^{{}^{\circ}}—$ 8_1,2_
3535.876	70	28273.45	5*d*′ ${\;}^{3}G_{4}^{{}^{\circ}}—$6*p*″ ^3^D_3_
3537.561	10*c*	28259.99	6*s*″ ${\;}^{3}P_{1}^{{}^{\circ}}—$ 6*p*″ ^1^S_0_
3538.334	1*h*	28253.81	6*p*′ ^3^F_2_— 6*d*′ $13_{1,2}^{{}^{\circ}}$
3539.628	1	28243.48	
3541.605	100	28227.72	6*s*″ ${\;}^{1}P_{1}^{{}^{\circ}}—$ 7*p*′ ^1^D_2_
3543.319	1	28214.06	5*d*′ ${\;}^{3}F_{2}^{{}^{\circ}}—$ 5*f* ^3^F_2_
3547.170	1	28183.44	
3549.734	2	28163.08	5*d*′ ${\;}^{1}D_{2}^{{}^{\circ}}—$ 8*p* ^3^P_2_
3552.196	100*c*	28143.56	5*d*′ ${\;}^{3}D_{2}^{{}^{\circ}}—$ 4*f* ^3^F_3_
3552.782	20	28138.92	5*d*′ ${\;}^{3}D_{2}^{{}^{\circ}}—$ 6*p*″ ^3^D_2_
3553.034	8*c*	28136.92	5*d*′ ${\;}^{1}F_{3}^{{}^{\circ}}—$ 6*f* ^3^F_4_
3553.843	1	28130.52	
3554.873	5	28122.37	5*d*′ ${\;}^{1}D_{2}^{{}^{\circ}}—$ 7*p*′ ^3^P_1_
3555.039	2*c*	28121.05	5*d* ${\;}^{5}D_{4}^{{}^{\circ}}—$ 6*p*′ ^1^F_3_
3555.576	2	28116.81	
3558.008	50	28097.59	5*d*′ ${\;}^{3}S_{1}^{{}^{\circ}}—$ 4*f*′ ^3^P_1_
3560.542	200	28077.59	5*d*′ ${\;}^{3}S_{1}^{{}^{\circ}}—$ 7*p*′ ^3^P_2_
3563.001	5	28058.22	5*d*′ ${\;}^{3}G_{3}^{{}^{\circ}}—$ 5*f* ^3^F_4_
3566.158	2	28033.38	6*s*′ ${\;}^{3}D_{3}^{{}^{\circ}}—$ 4*f* ^5^F_4_
3569.696	1	28005.50	
3569.888	6	28004.09	6*p* ^3^P_2_— 7*s*′ ${\;}^{3}D_{2}^{{}^{\circ}}$
3570.278	10*c*	28001.03	5*d*′ ${\;}^{3}D_{2}^{{}^{\circ}}—$ 7*p* ^5^P_2_
3571.386	10*c*	27992.34	5*d* ${\;}^{5}D_{2}^{{}^{\circ}}—$ 6*p*′ ^1^F_3_
3571.769	10*d*	27989.34	5*d*′ ${\;}^{1}D_{2}^{{}^{\circ}}—$ 4*f*′ ^3^F_2_
3573.680	200*c*	27974.37	5*d* ${\;}^{5}D_{1}^{{}^{\circ}}—$ 6*p*′ ^3^p_2_
3575.861	70	27957.31	5*p*^5^ ${\;}^{3}P_{1}^{{}^{\circ}}—$ 6*p*′ ^3^D_2_
3576.826	1	27949.77	6*s*″ ${\;}^{1}P_{1}^{{}^{\circ}}—$ 7*p*′ ^3^F_2_
3579.477	1	27929.07	
3586.568	3*c*	27873.85	5*d*″ ${\;}^{3}D_{3}^{{}^{\circ}}—$ 6*f* ^3^F_4_
3586.946	10	27870.92	5*d*′ ${\;}^{1}D_{2}^{{}^{\circ}}—$ 8*p* ^3^P_1_
3587.105	1	27869.68	5*d*″ ${\;}^{3}F_{2}^{{}^{\circ}}—$ 13_2,3_
3587.363	3*c*	27867.68	5*d*′ ${\;}^{3}D_{2}^{{}^{\circ}}—$ 7*p* ^5^P_1_
3588.505	4	27858.81	
3592.738	15	27830.85	5*d*′ ${\;}^{3}F_{2}^{{}^{\circ}}—$ 5*f* ^3^F_3_
3592.738	4	27825.98	
3593.030	1*h*	27823.72	6*s*′ ${\;}^{1}D_{2}^{{}^{\circ}}—$ 4*f* ^3^F_2_
3594.691	2	27810.87	
3596.204	1	27799.17	
3597.133	4	27791.99	
3598.012	3	27785.20	
3606.902	1	27716.72	5*d*′ ${\;}^{3}G_{3}^{{}^{\circ}}—$ 5*f* ^3^F_3_
3611.680	8*h*	27680.05	5*d*′ ${\;}^{1}F_{3}^{{}^{\circ}}—$ 6*f* ^3^F_4_
3612.769	2	27671.7l	5*d*′ ${\;}^{1}D_{2}^{{}^{\circ}}—$7*p*′ ^3^D_1_
3615.372	3	27651.78	5*d*″ ${\;}^{3}D_{2}^{{}^{\circ}}—$ 2_2,3_
3615.868	20*d*	27647.99	5*d* ${\;}^{5}D_{2}^{{}^{\circ}}—$ 6*p*′ ^1^P_1_
3617.136	1	27638.30	
3618.014	6	27631.59	5*d*′ ${\;}^{1}D_{2}^{{}^{\circ}}—$ 4*f*′ ^3^D_3_
3618.652	6	27626.72	5*d*′ ${\;}^{3}F_{2}^{{}^{\circ}}—$ 5*f* ^5^F_3_
3620.408	1	27613.32	5*d*″ ${\;}^{3}D_{2}^{{}^{\circ}}—$ 8*f* ^5^F_3_
3620.691	1	27611.16	6*p* ${\;}^{3}P_{0}^{{}^{\circ}}—$ 7*s*′ ${\;}^{3}D_{1}^{{}^{\circ}}$
3626.755	20*c*	27565.00	5*p*^5^ ${\;}^{1}P_{1}^{{}^{\circ}}—$ 6*p*″ ^3^D_1_
3627.507	15	27559.28	5*d*′ ${\;}^{1}D_{2}^{{}^{\circ}}—$ 4*f*′ ^3^F_3_
3627.785	3	27557.17	5*d*′ ${\;}^{3}P_{2}^{{}^{\circ}}—$ 7*p*′ ^3^D_2_
3631.880	6*c*	27526.10	6*s*′ ${\;}^{1}D_{2}^{{}^{\circ}}—$ 4*f* ^3^F_3_
3632.500	10	27521.40	6*s*′ ${\;}^{1}D_{2}^{{}^{\circ}}—$ 6*p*″ ^3^D_2_
3633.665	1	27512.58	5*d*′ ${\;}^{3}G_{3}^{{}^{\circ}}—$ 5*f* ^5^F_3_
3640.432	1*c*	27461.44	
3641.130	1	27456.18	
3648.812	2*h*	27398.37	6*p*′ ^1^D_2_— $24_{1,}^{{}^{\circ}}{\;}_{2}$
3649.144	3*h*	27305.88	
3650.800	20*c*	27383.46	6*s*′ ${\;}^{1}D_{2}^{{}^{\circ}}—$ 7*p* ^5^P_2_
3652.299	4	27372.22	6*p*′ ${\;}^{3}D_{2}^{{}^{\circ}}—$ 6*d*′ $13_{1,}^{{}^{\circ}}{\;}_{2}$
3653.355	15	27364.30	5*d*′ ${\;}^{3}S_{1}^{{}^{\circ}}—$ 7*p*′ ^3^P_1_
3655.423	5*h*	27348.82	5*d*′ ${\;}^{3}D_{3}^{{}^{\circ}}—$ 4*f* ^3^F_2_
3657.060	100	27336.58	6*s* ${\;}^{3}S_{1}^{{}^{\circ}}—$ 6*p*′ ^3^D_2_
3661.792	50*c*	27301.26	5*d*′ ${\;}^{3}D_{3}^{{}^{\circ}}—$ 4*f* ^3^F_4_
3662.816	2	27293.62	
3671.889	1	27226.19	
3677.874	3*c*	27181.88	6*s*″ ${\;}^{3}P_{2}^{{}^{\circ}}—$ 4*f*′ ^3^P_2_
3685.694	3*c*	27124.21	5*d*″ ${\;}^{1}D_{2}^{{}^{\circ}}—$ 4*f*′ ^1^D_2_
3692.266	5*c*	27075.93	5*p*^5^ ${\;}^{3}P_{1}^{{}^{\circ}}—$ 6*p*′ ^3^F_2_
3695.633	1*c*	27051.26	5*d*′ ${\;}^{3}D_{3}^{{}^{\circ}}—$ 4*f* ^3^F_3_
3696.292	15*c*	27046.44	5*d*′ ${\;}^{3}D_{3}^{{}^{\circ}}—$ 6*p*″ ^3^D_2_
3700.027	1	27019.14	5*d*″ ${\;}^{1}D_{2}^{{}^{\circ}}—$ 4*f*′ ^1^F_3_
3700.703	1	27014.21	
3703.426	5*c*	26994.34	5*d*′ ${\;}^{1}F_{3}^{{}^{\circ}}—$ 4*f*′ ^1^F_3_
3709.498	40*c*	26950.16	5*d* ${\;}^{3}D_{1}^{{}^{\circ}}—$ 6*p*′ ^1^D_2_
3714.466	1*c*	26014.11	6*s*″ ${\;}^{3}P_{2}^{{}^{\circ}}—$ 7*p*′ ^3^P_2_
3715.233	5*c*	26908.56	5*d*′ ${\;}^{3}D_{3}^{{}^{\circ}}—$ 7*p* ^5^P_2_
3716.170	50	26901.77	5*d* ${\;}^{5}D_{1}^{{}^{\circ}}—$ 6*p*′ ^3^P_0_
3725.274	1	26836.03	5*d*″ ${\;}^{3}D_{3}^{{}^{\circ}}—$ 4*f*′ ^1^D_2_
3727.200	2	26822.16	5*d*′ ${\;}^{3}P_{1}^{{}^{\circ}}—$7*f* ^3^F_2_
3727.330	10	26821.23	5*d*″ ${\;}^{1}D_{2}^{{}^{\circ}}—$ 7*p*′ ^1^D_2_
3729.580	1	26805.05	5*d*′ ${\;}^{1}D_{2}^{{}^{\circ}}—$ 5*f* ^3^F_3_
3710.890	25	26795.64	5*d*″ ${\;}^{1}D_{2}^{{}^{\circ}}—$ 7*p*′ ^1^F_3_
3734.366	60*c*	26770.70	5*d*′ ${\;}^{1}F_{3}^{{}^{\circ}}—$ 7*p*′ ^1^F_3_
3739.327	2	26735.18	6*d* ${\;}^{5}D_{3}^{{}^{\circ}}—$ 9*f* ^5^F_4_?
3740.358	4*c*	26727.81	5*d*″ ${\;}^{3}D_{3}^{{}^{\circ}}—$ 7*p*′ ^3^F_3_
3740.900	2	2672:3.94	
3742.138	200	26715.10	5*d*′ ${\;}^{1}F_{3}^{{}^{\circ}}—$ 7*p*′ ^3^F_4_
3744.138	1	26700.83	6*d* ${\;}^{5}D_{2}^{{}^{\circ}}—$ 9*f* ^5^F_3_?
3745.553	6*c*	26690.74	$\left\{ \begin{array}{l} 4f{\;}^{3}F_{3}—\;50_{3,4}^{{}^{\circ}}\\ 6d{\;}^{5}D_{4}^{{}^{\circ}}—\;9f{\;}^{5}F_{5}? \end{array}\right.\;$
3746.008	3*c*	26687.50	$\left\{ \begin{array}{l} 6p^{\prime }{\;}^{1}P_{1}—\;6d^{\prime }\;15_{1}^{{}^{\circ}}\\ 6d{\;}^{5}D_{4}^{{}^{\circ}}—\;9f{\;}^{5}F_{4}? \end{array}\right.\;$
3748.299	4*c*	26671.19	5*d*″ ${\;}^{1}F_{3}^{{}^{\circ}}—$ 11_3,4_
3764.694	2	26555.04	6*p*′ ${\;}^{3}D_{1}^{{}^{\circ}}—$ 6*d*′ $7_{1,}^{{}^{\circ}}{\;}_{2}$
3766.364	3	2654:3.26	5*d*″ ${\;}^{1}D_{2}^{{}^{\circ}}—$ 7*p*′ ^3^F_2_
3767.804	4*c*	26533.12	5*d*″ ${\;}^{3}D_{3}^{{}^{\circ}}—$ 7*p*′ ^1^D_2_
3769.287	1	26522.68	
3769.891	5	26518.43	5*d*′ ${\;}^{1}F_{3}^{{}^{\circ}}—$ 7*p*′ ^3^F_2_
3771.453	10	26507.45	5*d*″ ${\;}^{3}D_{3}^{{}^{\circ}}—$ 7*p*′ ^1^F_3_
3772.506	1	26500.05	4*f* ${\;}^{5}F_{5}^{{}^{\circ}}—$ 12*g* ${\;}^{5}G_{6}^{{}^{\circ}}$
3778.068	2	26461.04	5*d*′ ${\;}^{3}F_{3}^{{}^{\circ}}—$ 7*p* ^3^P_2_
3778.913	10*c*	26455.12	6*s* ${\;}^{3}S_{1}^{{}^{\circ}}—$ 6*p*′ ^3^F_2_
3779.374	25	26451.90	5*d*″ ${\;}^{3}D_{3}^{{}^{\circ}}—$ 7*p*′ ^3^F_4_
3781.442	20*c*	26437.43	5*d*′ ${\;}^{3}D_{2}^{{}^{\circ}}—$ 6*p*″ ^3^D_1_
3787.772	1	26393.25	4*f* ^3^F_2_— $50_{3,}^{{}^{\circ}}{\;}_{4}$
3789.289	5	26382.68	5*d* ${\;}^{5}D_{4}^{{}^{\circ}}—$ 6*p*′ ^3^F_3_
3792.147	1	26362.80	
3793.419	10	26353.96	5*d*′ ${\;}^{1}S_{0}^{{}^{\circ}}—$ 4*f*′ ^3^P_1_
3800.176	2	26307.10	
3801.073	2	26300.89	
3801.453	5*d*?	26298.26	5*d*′ ${\;}^{1}F_{3}^{{}^{\circ}}—$ 4*f*′ ^3^G_3_
3804.274	1	26278.76	5*d*′ ${\;}^{3}D_{1}^{{}^{\circ}}—$ 6*p*″ ^1^P_1_
3804.720	1	26275.68	
3804.843	6	26274.83	6*s*″ ${\;}^{1}P_{1}^{{}^{\circ}}—$ 4*f*′ ^3^P_0_
3807.840	10	26254.16	5*d* ${\;}^{5}D_{2}^{{}^{\circ}}—$ 6*p*′ ^3^F_3_
3809.651	5e	26241.68	6*s*″ ${\;}^{3}P_{2}^{{}^{\circ}}—$ 8*p* ^3^P_2_
3815.519	1*c*	26200.82	6*s*″ ${\;}^{3}P_{2}^{{}^{\circ}}—$ 7*p*′ ^3^P_1_
3818.597	2	26180.20	6*p*′ ^3^D_1_—7*d* ${\;}^{3}D_{1}^{{}^{\circ}}$
3826.022	1	26129.39	
3829.355	1	26106.65	5*d*′ ${\;}^{1}F_{3}^{{}^{\circ}}—$ 4*f*′ ^3^F_4_
3833.736	35*c*	26076.82	5*d* ${\;}^{3}D_{3}^{{}^{\circ}}—$ 6*p*′ ^1^D_2_
3842.914	25	26014.54	5*d* ${\;}^{5}D_{2}^{{}^{\circ}}—$ 6*p*′ ^3^D_2_
3848.808	1	25974.70	
3850.818	8	25961.15	6*p* ^5^P_2_— 6*d* ${\;}^{3}D_{3}^{{}^{\circ}}$
3851.243	1	25958.28	
3857.334	1	25917.29	4*f* ^5^F_5_— 11*g* ${\;}^{5}G_{6}^{{}^{\circ}}$
3870.276	1	25830.63	
3871.878	3	25819.94	6*s*′ ${\;}^{1}D_{2}^{{}^{\circ}}—$ 6*p*″ ^3^D_1_
3877.198	25	25784.51	5*p*^5^ ${\;}^{3}P_{1}^{{}^{\circ}}—$ 6*p*′ ^3^D_1_
3879.028	5	25772.35	5*d*′ ${\;}^{3}P_{2}^{{}^{\circ}}—$7*p*′ ^1^P_1_
3884.064	4	25738.94	6*p*′^1^P_1_— 6*d*′ $13_{1,2}^{{}^{\circ}}$
3893.000	70*c*	25679.85	5*p*^5^ ${\;}^{3}P_{0}^{{}^{\circ}}—$ 6*p*′ ^3^P_1_
3895.547	10*c*	25663.06	5*d*′ ${\;}^{1}P_{1}^{{}^{\circ}}—$4*f*′ ^1^P_1_
3901.118	20*c*	25626.42	5*d*′ ${\;}^{3}P_{0}^{{}^{\circ}}—$ 6*p*″ ^3^D_1_
3904.896	1	25601.62	
3905.917	3*c*	25594.93	5*d*′ ${\;}^{1}P_{0}^{{}^{\circ}}—$ 4*f*′ ^1^D_2_
3907.201	100	25586.52	5*d* ${\;}^{3}D_{2}^{{}^{\circ}}—$ 6*p*′ ^3^P_1_
3908.940	1	25575.14	5*d*″ ${\;}^{3}D_{2}^{{}^{\circ}}—$ 7*f*′ ^3^F_2_
3909.708	1	25570.12	5*d* ${\;}^{5}D_{3}^{{}^{\circ}}—$ 6*p*′ ^3^F_2_
3910.678	2	25563.77	
3915.234	30	25534.03	5*d*′ ${\;}^{3}F_{3}^{{}^{\circ}}—$ 4*f* ^3^F_4_
3924.018	2*c*	25476.87	6*p*′ ^1^F_3_— 6*d*′ $14_{3}^{{}^{\circ}}$
3931.169	10*c*	25430.53	5*d*′ ${\;}^{1}P_{1}^{{}^{\circ}}—$ 7*p*′ ^3^P_0_
3931.442	15	25428.76	5*d*′ ${\;}^{3}P_{2}^{{}^{\circ}}—$ 7*p*′ ^3^F_2_
3937.222	60*c*	25391.43	6*s*′ ${\;}^{3}D_{2}^{{}^{\circ}}—$ 6*p*′ ^1^D_2_
3942.476	20*d*	25357.59	5*d*′ ${\;}^{1}P_{1}^{{}^{\circ}}—$ 7*p*′ ^1^P_1_
3944.019	1	25347.67	
3945.000	2*c*	25341.37	6*s*″ ${\;}^{3}P_{1}^{{}^{\circ}}—$ 6*p*″ ^1^D_2_
3952.724	2*w*	25291.85	5*d*′ ${\;}^{1}P_{1}^{{}^{\circ}}—$ 7*p*′ ^1^D_2_
3953.978	2	25283.83	5*d*′ ${\;}^{3}F_{3}^{{}^{\circ}}—$ 4*f* ^3^F_3_
3960.504	1	25242.17	
3961.538	1	25235.58	6*d* ${\;}^{5}D_{2}^{{}^{\circ}}—$ 8*f* ^3^F_3_
3962.959	15*d*	25226.53	5*d*′ ${\;}^{3}D_{1}^{{}^{\circ}}—$ 6*p*″ ^3^P_2_
3965.544	50*c*	25210.09	5*d* ${\;}^{3}D_{2}^{{}^{\circ}}—$ 6*p*′ ^3^P_2_
3965.772	10	25208.64	5*d*′ ${\;}^{3}P_{2}^{{}^{\circ}}—$ 4*f*′ ^3^G_3_
3972.839	30	25163.80	6*s* ${\;}^{3}S_{1}^{{}^{\circ}}—$6*p*′ ^3^D_1_
3973.481	3	25159.73	6*d* ${\;}^{5}D_{3}^{{}^{\circ}}—$ 8*f* ^5^F_4_
3974.067	1	25156.02	6*d* ${\;}^{5}D_{3}^{{}^{\circ}}—$ 8*f* ^5^F_3_
3974.953	1	25150.42	4*f* ^5^F_5_—10*g* ${\;}^{5}G_{6}^{{}^{\circ}}$
3977.968	1	25131.35	6*d* ${\;}^{5}D_{2}^{{}^{\circ}}—$ 8*f* ^5^F_2_
3980.433	2	25115.79	6*d* ${\;}^{5}D_{2}^{{}^{\circ}}—$ 8*f* ^5^F_3_
3981.043	1	25111.94	6*d* ${\;}^{5}D_{4}^{{}^{\circ}}—$ 8*f* ^5^F_4_
3983.574	4	25095.99	6*d* ${\;}^{5}D_{4}^{{}^{\circ}}—$ 8*f* ^5^F_5_
3988.076	15	25067.66	5*d*′ ${\;}^{3}S_{1}^{{}^{\circ}}—$ 6*p*″ ^1^S_0_
3990.835	1	25050.33	
3996.642	5	25013.93	5*d*′ ${\;}^{1}P_{1}^{{}^{\circ}}—$ 7*p*′ ^3^F_2_
4006.349	6	24953.33	6*p*′ ^3^D_1_— 7*d* ${\;}^{3}D_{2}^{{}^{\circ}}$
4013.765	4	24907.22	6*p*′ ^3^D_1_— 6*d*′ $5_{1}^{{}^{\circ}}$
4017.235	2*c*	24885.71	6*p* ^5^P_3_– 6*d* ${\;}^{3}D_{3}^{{}^{\circ}}$
4019.310	1	24872.86	
4036.092	50*c*	24769.44	5*d* ${\;}^{3}D_{2}^{{}^{\circ}}—$ 6*p*′ ^3^D_3_
4043.893	20*c*	24721.66	6*s*″ ${\;}^{3}P_{1}^{{}^{\circ}}—$ 6*p*″ ^1^P_1_
4060.184	2	24622.47	5*d* ${\;}^{5}D_{0}^{{}^{\circ}}—$ 6*p*′ ^3^D_1_
4077.366	1	24518.72	
4078.556	1	24511.56	
4079.877	1	24503.62	6*p*′ ^1^P_1_— 6*d*′ $10_{2}^{{}^{\circ}}$
4081.378	5	24494.61	6*p*′ ^3^D_1_— 7*d* ${\;}^{5}D_{0}^{{}^{\circ}}$
4086.888	1	24461.59	
4089.598	3	24445.38	5*d* ${\;}^{5}D_{1}^{{}^{\circ}}—$ 6*p*′ ^3^D_2_
4094.310	1	24417.25	6*p*′ ^3^P_2_— 9*s* ${\;}^{3}S_{1}^{{}^{\circ}}$
4094.598	5	24415.53	6*p*′ ^3^P_1_— 6*d*′ $15_{1}^{{}^{\circ}}$
4095232	1*c*	24411.75	
4100.186	2	24382.26	6*p*′ ^3^D_2_— 6*d*′ $7_{1,2}^{{}^{\circ}}$
4102.895	6	24366.16	6*p*′ ^3^D_3_— 6*d*′ $14_{3}^{{}^{\circ}}$
4103.463	1	24362.78	6*p*′ ^3^D_1_— 7*d* ${\;}^{5}D_{2}^{{}^{\circ}}$
4107.818	3	24336.96	
4109.658	2	24326.06	
4111.646	3	24314.30	6*p*′ ^3^F_3_— 6*d*′ $8_{4}^{{}^{\circ}}$
4115.615	3	24290.85	
4116.294	6	24286.84	6*p*′ ^3^F_2_— 7*d* ${\;}^{3}D_{3}^{{}^{\circ}}$
4116.438	1	24286.00	
4116.823	3	24283.72	6*p*′ ^3^D_3_— 6*d*′ $13_{1,2}^{{}^{\circ}}?$
4117.892	3	24277.42	
4118.813	1	24271.99	6*p*″ ^3^D_1_— $32_{1}^{{}^{\circ}}$
4119.599	1	24267.36	6*p*′ ^3^D_2_— 6*d*′ $6_{1,2}^{{}^{\circ}}$
4120.190	8	24263.88	
4121.281	3*c*	24257.46	6*s*′ ${\;}^{3}D_{1}^{{}^{\circ}}—$ 6*p*′ ^1^D_2_
4122.931	4	24247.75	6*s*″ ${\;}^{1}P_{1}^{{}^{\circ}}—$ 7*p*′ ^3^D_1_
4124.536	2	24238.31	
4126.093	10	24229.17	6*p*′ ^3^D_1_— 6*d*′ $4_{0}^{{}^{\circ}}$
4135.723	1	24172.75	4*f* ^5^F_4_— 9*g* ${\;}^{3}G_{5}^{{}^{\circ}}$
4136.482	2	24168.32	4*f* ^5^F_4_— 9*g* ${\;}^{5}G_{5}^{{}^{\circ}}$
4138.030	5*c*	24159.28	6*p*′ ^1^F_3_— 6*d*′ $10_{2}^{{}^{\circ}}$
4139.702	1	24149.52	
4142.014	2	24136.04	
4145.865	3	24113.62	4*f* ^5^F_5_— 9*g* ${\;}^{5}G_{6}^{{}^{\circ}}$
4153.136	2	24071.40	4*f* ^5^F_2_*—* 9*g* ${\;}^{5}G_{3}^{{}^{\circ}}$
4158.425	3	24040.79	6*p*′ ^3^P_1_— 9*s* ${\;}^{3}S_{1}^{{}^{\circ}}$
4160.646	4*c*	24027.96	6*d* ${\;}^{3}D_{1}^{{}^{\circ}}—$ 8_1, 2_
4162.565	1	24016.88	5*d*′ ${\;}^{3}D_{1}^{{}^{\circ}}—$ 6*p*″ ^3^S_1_
4162.689	1	24016.16	4*f* ^5^F_1_— 9*g* ${\;}^{5}G_{2}^{{}^{\circ}}$
4166.838	2	23992.25	5*d*″ ${\;}^{1}D_{2}^{{}^{\circ}}—$ 7*p*′ ^3^D_3_
4171.155	1*c*	23967.42	5*d*′ ${\;}^{1}F_{3}^{{}^{\circ}}—$ 7*p*′ ^3^D_3_
4173.810	40*c,Z*	23952.17	5*d* ${\;}^{3}D_{1}^{{}^{\circ}}—$ 6*p*′ ^3^P_1_
4177.179	4	23932.86	5*d*′ ${\;}^{3}F_{2}^{{}^{\circ}}—$ 6*p*″ ^1^D_2_
4178.440	1	23925.63	6*p*′ ^3^P_2_— 6*d*′ $14_{3}^{{}^{\circ}}$
4179.928	1	23917.12	
4183.035	1	23899.35	
4183.516	2	23896.60	
4192.862	1	23843.34	6*p*′ ^3^P_2_— 6*d*′ $13_{1,2}^{{}^{\circ}}$
4197.190	2	23818.75	5*d*′ ${\;}^{3}G_{3}^{{}^{\circ}}—$ 6*p*″ ^1^D_2_
4206.534	1	23765.84	
4217.476	3*h*	23704.19	5*d*″ ${\;}^{3}D_{3}^{{}^{\circ}}—$ 7*p*′ ^3^D_3_
4219.138	10	23694.85	6*p* ^3^P_1_— 6*d* ${\;}^{3}D_{1}^{{}^{\circ}}$
4224.993	10	23662.01	6*p*′ ^3^F_2_— 7*d* ${\;}^{3}D_{2}^{{}^{\circ}}$
4225.542	50*c, Z*	23658.94	5*d* ${\;}^{3}D_{2}^{{}^{\circ}}—$ 6*p*′ ^1^F_3_
4231.185	1	23627.39	4*f* ^3^F_3_— 9*g* ${\;}^{3}G_{4}^{{}^{\circ}}$
4233.249	2	23615.87	6*p*′ ^3^F_2_*—* 6*d*′ $5_{1}^{{}^{\circ}}$
4235.511	30*Z*	23603.26	6*p* ^3^P_1_— 6*d* ${\;}^{3}D_{2}^{{}^{\circ}}$
4240.441	15*c*	23575.82	5*d* ${\;}^{3}D_{1}^{{}^{\circ}}—$ 6*p*′ ^3^P_2_
4242.545	1	23564.12	5*d* ${\;}^{5}D_{1}^{{}^{\circ}}—$ 6*p*′ ^3^F_2_
4248.287	1	23532.27	
4249.711	1*w*	23524.39	
4254.193	1	23499.61	6*p*″ ^3^D_1_— $29_{1}^{{}^{\circ}}$
4254.282	1	23499.11	
4256.129	2	23488.92	
4257.606	1*w*	23480.77	
4260.128	3*c*	23466.87	6*p*′ ^3^P_1_— 6*d*′ $13_{1,2}^{{}^{\circ}}$
4265.801	2	23435.66	
4266.925	1	23429.49	4*f* ^3^F_3_— $37_{2}^{{}^{\circ}}$
4271.310	6	23405.43	6*p*′ ^3^D_2_— 7*d* ${\;}^{3}D_{3}^{{}^{\circ}}$
4276.015	1	23379.68	4*f* ^3^F_4_— 9*g* ${\;}^{3}G_{5}^{{}^{\circ}}$
4279.188	4*c*	23362.35	6*s*″ ${\;}^{3}P_{0}^{{}^{\circ}}—$ 6*p*″ ^3^S_1_
4281.834	5*c*	23347.91	6*p*′ ^1^F_3_— 6*d*′ $9_{3}^{{}^{\circ}}$
4282.912	4	23342.03	
4287.924	2	23314.75	5*d* ${\;}^{3}D_{2}^{{}^{\circ}}—$ 6*p*′ ^1^P_1_
4288.215	6	23313.17	5*d*′ ${\;}^{3}F_{2}^{{}^{\circ}}—$ 6*p*″ ^1^P_1_
4291.923	30	23293.03	6*p*′ ^3^D_1_— 6*d*′ $1_{2}^{{}^{\circ}}$
4296.286	10	23269.37	6*p*′ ^3^P_2_— 6*d*′ $12_{1}^{{}^{\circ}}$
4315.481	10	23165.87	6*p*′ ^3^F_3_— 7*d* ${\;}^{3}D_{3}^{{}^{\circ}}$
4316.897	5*c*	23158.28	5*d*′ ${\;}^{3}P_{2}^{{}^{\circ}}—$ 4*f*′ ^3^P_2_
4319.623	1*w*	23143.66	6*p*′ ^3^F_2_— 7*d* ${\;}^{5}D_{3}^{{}^{\circ}}$
4322.756	50	23126.89	5*p*^5^ ${\;}^{1}P_{1}^{{}^{\circ}}—$ 6*p*′ ^1^D_2_
4333.159	2*c*	23071.36	6*p*′ ^3^F_2_— 7*d* ${\;}^{5}D_{2}^{{}^{\circ}}$
4334.246	2	23065.58	
4337.427	2	23048.66	6*p*′ ^3^D_3_— 6*d*′ $10_{2}^{{}^{\circ}}$
4338.224	1*c*	23044.43	5*d*″ ${\;}^{3}D_{3}^{{}^{\circ}}—$ 8*p* ^3^P_2_
4342.078	20*Z*	23023 98	6*p* ^5^P_1_— 6*d* ${\;}^{5}D_{1}^{{}^{\circ}}$
4355.036	1	22955.47	
4362.456	10	22916.43	6*p* ^5^P_2_— 6*d* ${\;}^{5}D_{1}^{{}^{\circ}}$
4364.224	5	22907.14	5*d*′ ${\;}^{1}D_{2}^{{}^{\circ}}—$ 6*p*″ ^1^D_2_
4368.247	2	22886.05	
4369.834	2	22877.74	5*d*′ ${\;}^{3}P_{2}^{{}^{\circ}}—$ 7*p*′ ^3^D_3_
4371.722	1*c*	22867.86	
4373.686	3	22857.59	6*p*″ ^3^P_0_*—* $33.5_{1}^{{}^{\circ}}$
4374.760	3	22851.98	6*d* ${\;}^{5}D_{3}^{{}^{\circ}}—$ 7*f* ^5^F_3_
4376.139	100*Z*	22844.78	
4376.745	8	22841.61	6*d* ${\;}^{5}D_{3}^{{}^{\circ}}—$ 7*f* ^5^F_4_
4379.048	2	22829.60	
4382.489	5	22811.68	6*d* ${\;}^{5}D_{2}^{{}^{\circ}}—$ 7*f* ^5^F_3_
4383.998	15	22803.82	6*d* ${\;}^{5}D_{4}^{{}^{\circ}}—$ 7*f* ^5^F_5_
4385.904	2	22793.91	6*d* ${\;}^{5}D_{4}^{{}^{\circ}}—$ 7*f* ^5^F_4_
4388.458	10	22780.65	6*p*′ ^3^D_2_— 7*d* ${\;}^{3}D_{2}^{{}^{\circ}}$
4390.278	20*c*	22771.20	6*p*″ ^3^D_1_— $21_{1}^{{}^{\circ}}$
4394.555	6*c*	22749.04	6*p*′ ^1^P_1_— 6*d*′ $7_{1,2}^{{}^{\circ}}$
4398.585	10	22728.20	6*p*′ ^3^D_1_— 5*d*″ ${\;}^{3}P_{1}^{{}^{\circ}}$
4399.037	60*c*	22725.86	6*p*′ ^3^F_2_— 6*d*′ $3_{3}^{{}^{\circ}}$
4399.592	1	22722.99	4*f* ^5^F_4_— 8*g* ${\;}^{3}G_{5}^{{}^{\circ}}$
4400.741	4*c*	22717.06	4*f* ^5^F_4_— 8*g* ${\;}^{5}G_{5}^{{}^{\circ}}$
4403.566	60*c, Z*	22702.49	5*d* ${\;}^{3}D_{3}^{{}^{\circ}}—$ 6*p*′ ^3^P_2_
4404.580	7*d*	22697.26	5*d*′ ${\;}^{1}F_{3}^{{}^{\circ}}—$ 4*f*′ ^3^G_4_
4405.577	1	22692.12	4*f* ^5^F_3_— 8*g* ${\;}^{3}G_{4}^{{}^{\circ}}$
4406.097	3	22689.45	4*f* ^5^F_3_— 8*g* ${\;}^{5}G_{4}^{{}^{\circ}}$
4408.954	60*Z*	22674.74	6*p* ^3^P_2_*—* 6*d* ${\;}^{3}D_{3}^{{}^{\circ}}$
4411.198	5	22663.21	4*f* ^5^F_5_— 8*g* ${\;}^{5}G_{6}^{{}^{\circ}}$
4412.333	50*Z*	22657.38	6*p*′ ^3^F_2_— 6*d*′ $2_{3}^{{}^{\circ}}$
4416.569	10	22635.65	5*d*′ ${\;}^{3}D_{1}^{{}^{\circ}}—$ 7*p* ^3^P_0_
4416.890	10	22634.00	6*p* ^3^P_2_— 6*d* ${\;}^{3}D_{2}^{{}^{\circ}}$
4417.567	10*c*	22630.54	$\{\;\begin{array}{llll} 6p^{\prime } & {\;}^{1}P_{1}— & 6d^{\prime } & 6_{1,2}^{{}^{\circ}}\\ 6p\prime \prime & {\;}^{3}P_{1}— & & 32_{1}^{{}^{\circ}} \end{array}$
4419.496	2	22620.66	4*f* ^5^F_2_— 8*g* ${\;}^{5}G_{3}^{{}^{\circ}}$
4421. 965	30*c*	22608.03	6*p*′ ^3^P_2_— 6*d*′ $10_{2}^{{}^{\circ}}$
4423.725	100*c*, *Z*	22599.03	6*p* ^5^P_1_— 6*d* ${\;}^{5}D_{2}^{{}^{\circ}}$
4428.195	50*d, Z*	22576.22	6*p*′ ^1^F_3_— 6*d*′ $8_{4}^{{}^{\circ}}$
4430.130	2	22566.36	4*f* ^5^F_1_— 8*g* ${\;}^{5}G_{2}^{{}^{\circ}}$
4435.104	5	22541.05	$\{\;\begin{array}{llll} 6p^{\prime } & {\;}^{3}F_{3}— & 7d & {\;}^{3}D_{2}^{{}^{\circ}}\\ 5d\prime \prime & {\;}^{3}D_{3}^{{}^{\circ}}— & 8p & {\;}^{5}P_{3}? \end{array}$
4436.937	3	22531.74	5*d*′ ${\;}^{3}P_{2}^{{}^{\circ}}—$ 4*f*′ ^3^D_2_
4442.562	200*Z*	22503.21	5*d* ${\;}^{3}D_{1}^{{}^{\circ}}—$ 6*p*′ ^3^P_0_
4444.873	150*Z*	22491.51	6*p* ^5^P_2_— 6*d* ${\;}^{5}D_{2}^{{}^{\circ}}$
4445.872	3*c*	22486.46	5*d*′ ${\;}^{3}P_{1}^{{}^{\circ}}—$ 4*f*′ ^1^P_1_
4446.742	40*Z*	22482.06	6*p* ^5^P_1_— 6*d* ${\;}^{5}D_{0}^{{}^{\circ}}$
4451.149	25*c*	22459.80	6*s*″ ${\;}^{3}P_{1}^{{}^{\circ}}—$ 6*p*″ ^3^S_1_
4452.858	300*Z*	22451.18	6*p* ^5^P_2_— 6*d* ${\;}^{5}D_{3}^{{}^{\circ}}$
4456.250	10*c*	22434.10	5*d*″ ${\;}^{3}D_{3}^{{}^{\circ}}—$ 4*f*′ ^3^G_4_
4456.625	100*c, Z*	22432.20	6*s*′ ${\;}^{3}D_{3}^{{}^{\circ}}—$ 6*p*′ ^1^D_2_
4457.643	5	22427.08	
4458.474	20*c*	22422.90	6*p*′ ^1^F_3_— 5*g* ${\;}^{5}G_{4}^{{}^{\circ}}$
4460.185	30*Z*	22414.30	5*p*^4 3^P_1_— 5*p*^4 1^S_0_
4461.928	2	22405.54	7*p* ^5^P_1_— $31_{0}^{{}^{\circ}}$
4462.708	100	22401.63	6*s*″ ${\;}^{1}P_{1}^{{}^{\circ}}—$ 6*p*″ ^1^S_0_
4464.338	150*c, Z*	22393.45	6*s*′ ${\;}^{3}D_{2}^{{}^{\circ}}—$ 6*p*′ ^3^P_1_
4468.167	3*c*	22374.26	6*p*′ ^1^P_1_— 7*d* ${\;}^{3}D_{1}^{{}^{\circ}}$
4473.412	100*c*, *Z*	22348.03	5*d* ${\;}^{3}D_{3}^{{}^{\circ}}—$ 6*p*′ ^3^F_4_
4476.037	10*Z*	22334.92	6*p* ^3^P_0_— 6*d* ${\;}^{3}D_{1}^{{}^{\circ}}$
4479.656	30	22316.88	6*p*″ ^3^D_1_— $20_{1,2}^{{}^{\circ}}$
4483.730	1	22296.60	7*p* ^3^P_2_— 11*d* ${\;}^{3}D_{3}^{{}^{\circ}}$
4484.829	2*w*	22291.14	5*d*′ ${\;}^{1}F_{3}^{{}^{\circ}}—$ 5*f* ^3^F_4_
4487.234	7	22279.19	6*p*′ ^3^P_1_— 6*d*′ $11_{0}^{{}^{\circ}}$
4488.552	40*Z*	22272.65	5*d* ${\;}^{5}D_{1}^{{}^{\circ}}—$ 6*p*′ ^3^D_1_
4490.652	4	22262.23	6*p*′ ^3^D_2_— 7*d* ${\;}^{5}D_{3}^{{}^{\circ}}$
4490.912	3	22260.94	$5d_{3}^{\prime }$ ${\;}^{3}F_{2}^{{}^{\circ}}—$ 6*p*″ ^3^P_2_
4492.352	3	22253.81	5*d*′ ${\;}^{3}P_{1}^{{}^{\circ}}—$ 7*p*′ ^3^P_0_
4492.649	1*w*	22252.34	6*p*′ ^3^F_2_— 7*s*′ ${\;}^{3}D_{3}^{{}^{\circ}}$
4495.698	15*c*	22237.24	6*p*′ ^3^D_3_— 6*d*′ $9_{3}^{{}^{\circ}}$
4496.815	1	22231.72	6*p*′ ^3^P_1_— 6*d*′ $10_{2}^{{}^{\circ}}$
4497.535	7	22228.16	5*d*″ ${\;}^{3}P_{2}^{{}^{\circ}}—$ 7*p*′ ^1^F_3_
4497.708	10	22227.31	
4498.586	1	22222.97	7*p* ^5^P_2_— 10*d* ${\;}^{3}D_{3}^{{}^{\circ}}$
4499.604	20*c*	22217.94	5*d*′ ${\;}^{3}P_{2}^{{}^{\circ}}—$ 8*p* ^3^P_2_
4504.375	1	22194.41	6*p*″ ^3^P_1_— $31_{0}^{{}^{\circ}}$
4505.072	6	22190.97	7*p* ^5^P_1_— 10*d* ${\;}^{3}D_{2}^{{}^{\circ}}$
4505.265	5	22190.02	6*p*′ ^3^D_2_— 7*d* ${\;}^{5}D_{2}^{{}^{\circ}}$
4506.772	1	22182.60	4*f* ^3^F_3_— 8*g* ${\;}^{3}G_{3}^{{}^{\circ}}$
4507.129	2	22180.85	5*d*′ ${\;}^{3}P_{1}^{{}^{\circ}}$— 7*p*′ ^1^P_1_
4507.866	10	22177.22	$\{\;\begin{array}{llll} 5d^{\prime } & {\;}^{3}P_{2}^{{}^{\circ}}— & 7p^{\prime } & 3P_{1}\\ 4f & {\;}^{3}F_{3}— & 8g & {\;}^{3}G_{4}^{{}^{\circ}} \end{array}$
4508.441	1	22174.39	4*f* ^3^F_3_— 8*g* ${\;}^{5}G_{4}^{{}^{\circ}}$
4513.170	8*c*	22151.16	$\{\;\begin{array}{llll} 6p^{\prime } & {\;}^{3}F_{4}— & 6d^{\prime } & 9_{3}^{{}^{\circ}}\\ 7s & {\;}^{3}S_{1}^{{}^{\circ}}— & 7p^{\prime } & {\;}^{3}G_{2} \end{array}$
4513. 508	8*c*	22149.50	
4514.064	20*c*	22146.77	5*d*′ ${\;}^{3}G_{3}^{{}^{\circ}}$— 6*p*″ ^3^P_2_
4519.910	1	22118.13	7*p* ^3^P_1_— 12*s* ${\;}^{3}S_{1}^{{}^{\circ}}$?
4520.528	4	22115.10	5*d*′ ${\;}^{3}P_{1}^{{}^{\circ}}$— 7*p*′ ^1^D_2_
4526.672	2	22085.09	6*p*″ ^3^D_2_— 10*d* ${\;}^{3}D_{3}^{{}^{\circ}}$
4527.024	10	22083.37	6*p*′ ^3^F_3_— 7*d* ${\;}^{5}D_{4}^{{}^{\circ}}$
4529.898	1	22069.36	7*p* ^5^P_1_— $29_{1}^{{}^{\circ}}$
4532.296	1	22057.68	7*p* ^5^P_2_— 10*d* ${\;}^{3}D_{2}^{{}^{\circ}}$
4535.046	5	22044.31	5*d*′ ${\;}^{3}P_{2}^{{}^{\circ}}$— 4*f*′ ^3^F_2_
4537.599	5	22031.90	5*d*″ ${\;}^{3}F_{2}^{{}^{\circ}}$— 7*f* ^3^F_3_
4538.411	3*w*	22027.96	5*d*″ ${\;}^{3}D_{3}^{{}^{\circ}}$— 5*f* ^3^F_4_
4540.644	40*Z*	22017.13	6*s*′ ${\;}^{3}D_{2}^{{}^{\circ}}$— 6*p*′ ^3^P_2_
4541.306	8	22013.92	6*p*″ ^3^P_0_— $29_{1}^{{}^{\circ}}$
4543.833	25	22001.68	6*p*′ ^3^F_2_— 6*d*′ $1_{2}^{{}^{\circ}}$
4544.272	50	21999.55	5*d*′ ${\;}^{3}D_{2}^{{}^{\circ}}$— 6*p*′ ^1^D_2_
4544.339	20	21999.23	
4548.342	7	21979.87	6*p*″ ^3^P_1_— 10*d* ${\;}^{3}D_{2}^{{}^{\circ}}$
4549.071	4	21976.35	
4549.437	1	21974.58	5*d*″ ${\;}^{1}D_{2}^{{}^{\circ}}$— 5*f* ^3^F_3_
4556.012	1*w*	21942.87	
4558.758	4	21929.65	4*f* ^3^F_4_— 8*g* ${\;}^{3}G_{5}^{{}^{\circ}}$
4559.545	8*w*	21925.86	5*d*′ ${\;}^{3}P_{2}^{{}^{\circ}}$*—* 8*p* 3P_1_
4559.999	1	21923.68	4*f* ^3^F_4_— 8*g* ${\;}^{5}G_{5}^{{}^{\circ}}$
4560.612	15*Z*	21920.73	5*d* ${\;}^{3}D_{2}^{{}^{\circ}}$*—* 6*p*′ ^3^F_3_
4560.880	10	21919.45	5*d*′ ${\;}^{3}F_{2}^{{}^{\circ}}$*—* 6*p*″ ^3^D_3_
4568.06	3	21884.95	4*f* ^3^F_2_*—* 8*g* ${\;}^{3}G_{3}^{{}^{\circ}}$
4571.517	12	21868.44	6*p* ^5^P_2_— 5*d*″ ${\;}^{3}F_{3}^{{}^{\circ}}$
4573.543	2	21858.76	6*d* ${\;}^{3}D_{3}^{{}^{\circ}}$— 8*f* ^3^F_4_
4573.718	5	21857.92	5*d*′ ${\;}^{3}D_{1}^{{}^{\circ}}$— 7*p* ^3^P_2_
4574.090	20*Z*	21856.14	6*p* ^5^P_1_— 5*d*″ ${\;}^{3}D_{1}^{{}^{\circ}}$
4576.550	40*Z*	21844.39	6*p*′ ^3^D_2_— 6*d*′ $3_{3}^{{}^{\circ}}$
4578.017	1	21837.39	5*d*′ ${\;}^{3}P_{1}^{{}^{\circ}}$— 7*p*′ ^3^F_2_
4578.710	7	21834.09	7*p* ^5^P_1_— $25_{1,2}^{{}^{\circ}}$
4584.457	1	21806.72	6*d* ${\;}^{3}D_{2}^{{}^{\circ}}$— 8*f* ^3^F_3_
4584.752	2	21805.32	5*d*′ ${\;}^{3}G_{3}^{{}^{\circ}}$— 6*p*″ ^3^D_3_
4586.582	20	21796.62	6*p*′ ^3^P_2_— 6*d*′ $9_{3}^{{}^{\circ}}$
4589.495	1	21782.78	4*f* ^3^F_2_— 10*d* ${\;}^{3}D_{3}^{{}^{\circ}}$
4590.764	25	21776.76	
4590.930	20*Z*	21775.97	6*p*′ ^3^D_2_— 6*d*′ $2_{3}^{{}^{\circ}}$
4593.066	7	21765.85	6*d* ${\;}^{3}D_{3}^{{}^{\circ}}$— 8*f* ^3^F_3_
4596.720	6	21748.54	6*p* ^5^P_2_— 5d″ ${\;}^{3}D_{1}^{{}^{\circ}}$
4599.769	200*Z*	21734.13	
4600.365	1*w*	21731.31	
4604.309	1*c*	21712.70	
4605.924	1	21705.09	
4609.191	1	21689.71	
4611.224	40*c, Z*	21680.14	5*d* ${\;}^{3}D_{1}^{{}^{\circ}}$— 6*p*′ ^1^P_1_
4612.265	1	21675.25	7*p* ^3^P_2_— $35_{2,3}^{{}^{\circ}}$
4614.102	1	21666.62	
4621.854	100*Z*	21630.28	
4622.172	7	21628.79	
4622.347	20	21627.97	6*p*″ ^3^D_2_— $26_{3}^{{}^{\circ}}$
4623.417	8	21622.96	6*p*″ ^3^P_1_— $25_{1,2}^{{}^{\circ}}$
4624.603	1	21617.42	4*f* ^3^F_2_— 10*d* ${\;}^{3}D_{2}^{{}^{\circ}}$
4625.288	5	21614 22	5*d*′ ${\;}^{3}P_{2}^{{}^{\circ}}$— 4*f*′ ^3^F_3_
4627.292	5	21604.86	6*p*′ ^3^F_3_— 6*d*′ $3_{3}^{{}^{\circ}}$
4632.446	300*c, Z*	21580.82	6*s* ${\;}^{5}S_{2}^{{}^{\circ}}$— 6*p* ^3^P_2_
4633.365	50	21576.54	6*s*′ ${\;}^{3}D_{2}^{{}^{\circ}}$— 6*p*′ ^3^D_3_
4638.810	8	21551.21	6*p*′ ^3^P_0_— 7*d* ${\;}^{3}D_{1}^{{}^{\circ}}$
4640.831	150	21541.83	
4641.142	7	21540.38	
4641.984	30	21536.48	6*p*′ ^3^F_3_— 6*d*′ $2_{3}^{{}^{\circ}}$
4643.843	1	21527.86	
4647.472	1	21511.05	5*d*′ ${\;}^{1}P_{1}^{{}^{\circ}}$— 8*p* ^3^P_1_
4649.114	1	21503.45	
4651.324	1	21493.23	7*p* ^5^P_2_— 11*s* ${\;}^{5}S_{2}^{{}^{\circ}}$
4654.492	3	21478.60	
4657.302	30	21465.65	6*p*′ ^3^D_3_— 6*d*′ $8_{4}^{{}^{\circ}}$
4663.560	1*c*	21436.84	6*p*′ ^3^F_2_— 5*d*″ ${\;}^{3}P_{1}^{{}^{\circ}}$
4665.537	10*c*	21427.76	6*p*′ ^1^F_3_— 7*d* ${\;}^{3}D_{3}^{{}^{\circ}}$
4666.484	500*d*?	21423.41	6*p* ^5^P_3_*—* 6*d* ${\;}^{5}D_{4}^{{}^{\circ}}$
4668.116	20	21415.92	6*p* ^5^P_3_— 6*d* ${\;}^{5}D_{2}^{{}^{\circ}}$
4672.280	2	21396.83	7*p* ^5^P_2_— $24_{1,2}^{{}^{\circ}}$
4675.530	1000*Z*	21381.96	6*s*′ ${\;}^{1}D_{2}^{{}^{\circ}}$— 6*p*′ ^1^D_2_
4676.080	20	21379.45	6*p*′ ^3^F_4_— 6*d*′ $8_{4}^{{}^{\circ}}$
4676.902	75	21375.69	6*p* ^5^P_3_— 6*d* ${\;}^{5}D_{3}^{{}^{\circ}}$
4677.909	20*c*	21371.09	6*p*′ ^3^D_2_— 7*s*′ ${\;}^{3}D_{3}^{{}^{\circ}}$
4684.507	1*w*	21340.99	7*p* ^5^P_1_— $21_{1}^{{}^{\circ}}$
4684.928	2*w*	21339.07	5*d*″ ${\;}^{1}F_{3}^{{}^{\circ}}$— 7*f* ^3^F_4_
4685.349	1*w*	21337.15	
4687.468	5*c*	21327.51	6*p*′ ^3^D_3_— 5*g* ${\;}^{3}G_{4}^{{}^{\circ}}$
4687.890	20	21325.59	4*f* ^3^F_2_— $26_{3}^{{}^{\circ}}$
4702.468	25	21259.48	6*s*′ ${\;}^{3}D_{1}^{{}^{\circ}}$— 6*p*′ ^3^P_1_
4702.594	7	21258.91	6*p*″ ^3^D_2_— $24_{1,2}^{{}^{\circ}}$
4706.453	1*w*	21241.48	6*p*′ ^3^F_4_— 5*g* ${\;}^{3}G_{4}^{{}^{\circ}}$
4707.842	40	21235.21	5*d*′ ${\;}^{1}D_{2}^{{}^{\circ}}$— 6*p*″ ^3^P_2_
4709.844	20*c*	21226.18	6*p*′ ^3^F_4_— 5*g* ${\;}^{5}G_{4}^{{}^{\circ}}$
4711.430	25*c*	21219.04	6*p*′ ^3^F_4_— 5*g* ${\;}^{5}G_{5}^{{}^{\circ}}$
4712.498	1	21214.23	
4713.988	1*w*	21207.52	7*p* ^5^P_2_— $21_{1}^{{}^{\circ}}$
4722.102	12*c*	21171.08	5*d*″ ${\;}^{3}D_{2}^{{}^{\circ}}$— 4*f*′ ^1^D_2_
4726.535	20*c*	21151.23	5*d* ${\;}^{3}D_{3}^{{}^{\circ}}$— 6*p*′ ^1^F_3_
4730.366	25	21134.10	6*p* ^3^P_1_— 5*d*″ ${\;}^{3}F_{2}^{{}^{\circ}}$
4730.958	20*c*	21131.45	6*p*′ ^3^F_3_— 7*s*′ ${\;}^{3}D_{3}^{{}^{\circ}}$
4731.316	8	21129.85	6*p*″ ^3^P_1_— $21_{1}^{{}^{\circ}}$
4733.414	6	21120.49	6*p*′ ^3^D_2_— 6*d*′ $1_{2}^{{}^{\circ}}$
4737.544	2	21102.08	
4737.691	2	21101.42	
4737.828	4	21100.81	
4739.388	1	21093.87	7*p* ^5^P_3_— 11*s* ${\;}^{5}S_{2}^{{}^{\circ}}$
4742.810	50*c*	21078.65	6*s*″ ${\;}^{3}P_{1}^{{}^{\circ}}$— 7*p* ^3^P_0_
4744.797	3	21069.82	6*p*″ ^3^D_2_— $21_{1}^{{}^{\circ}}$
4748.978	20	21051.27	5*d*′ ${\;}^{3}F_{2}^{{}^{\circ}}$— 6*p*″ ^3^S_1_
4750. 846	1*c*	21042.99	6*p*′ ^1^D_2_— 9*s* ${\;}^{3}S_{1}^{{}^{\circ}}$
4751.798	4	21038.78	
4752.676	60	21034.89	6*s*″ ${\;}^{3}P_{0}^{{}^{\circ}}$— 7*p* ^3^P_1_
4753.825	1	21029.81	
4763.820	60	20985.68	6*s*″ ${\;}^{3}P_{2}^{{}^{\circ}}$— 6*p*″ ^1^D_2_
4765.519	30	20978.20	5*d*′ ${\;}^{3}D_{1}^{{}^{\circ}}$— 4*f* ^3^F_2_
4768.193	15	20966.44	6*p* ^3^P_2_— 5*d*″ ${\;}^{1}F_{3}^{{}^{\circ}}$
4770.456	5	20956.49	4*f* ^3^F_2_— $24_{1,2}^{{}^{\circ}}$
4770.857	1	20954.73	
4772. 841	1	20946.02	
4775.674	10	20933.60	
4782.000	6	20905.90	7*p* ^3^P_1_— 10*d* ${\;}^{3}D_{2}^{{}^{\circ}}$
4782.658	5	20903.03	7*p* ^3^P_2_— 10*d* ${\;}^{3}D_{3}^{{}^{\circ}}$
4784.798	50	20893.68	5*d*′ ${\;}^{1}D_{2}^{{}^{\circ}}$— 6*p*″ ^3^D_3_
4786.389	2*c*	20886.73	7*p* ^5^P_1_— $20_{1,2}^{{}^{\circ}}$
4787.228	50*c*	20883.07	6*s*′ ${\;}^{3}D_{1}^{{}^{\circ}}$— 6*p*′ ^3^P_2_
4787.776	3	20880.68	6*p*′ ^3^F_3_— 6*d*′ $1_{2}^{{}^{\circ}}$
4790.690	30	20867.98	5*d*″ ${\;}^{3}D_{2}^{{}^{\circ}}$— 7*p*′ ^1^D_2_
4792.522	1*w*	20860.01	5*d*′ ${\;}^{3}P_{2}^{{}^{\circ}}$— 5*f* ^3^F_3_
4796.576	25	20842.38	5*d*″ ${\;}^{3}D_{2}^{{}^{\circ}}$— 7*p*′ ^1^F_3_
4805.662	4*c*	20802.97	6*p*′ ^1^F_3_— 7*d* ${\;}^{3}D_{2}^{{}^{\circ}}$
4806.402	75*Z*	20799.77	5*d* ${\;}^{3}D_{2}^{{}^{\circ}}$— 6*p*′ ^3^F_2_
4807.320	3	20795.79	
4807.998	20	20792.86	6*p* ^5^P_3_— 5*d*″ ${\;}^{3}F_{3}^{{}^{\circ}}$
4812.106	1*w*	20775.11	
4813.902	2	20767.36	4*f* ^3^F_2_— $21_{1}^{{}^{\circ}}$
4825.452	1	20717.65	
4828.252	70*c, Z*	20705.64	5*p*^5^ ${\;}^{3}P_{2}^{{}^{\circ}}$— 6*p* ^3^P_2_
4831. 00	1	20691.29	
4832.238	5*c*	20688.56	6*p*′ ^1^P_1_— 7*d* ${\;}^{5}D_{0}^{{}^{\circ}}$
4833.747	3	20682.10	7*p* ^3^P_0_— $33.5_{1}^{{}^{\circ}}$
4835.178	40	20675.98	5*d*′ ${\;}^{3}D_{1}^{{}^{\circ}}$— 6*p*″ ^3^D_2_
4849.308	4*w*	20615.74	5*d*′ ${\;}^{3}D_{1}^{{}^{\circ}}$— 6*p*″ ^3^P_1_
4850.303^a^	60*c*	20611.51	6*s* ${\;}^{5}S_{2}^{{}^{\circ}}$— 6*p* ^3^P_1_
4851.017	20	20608.47	6*p*′ ^3^D_1_— 7*s*′ ${\;}^{3}D_{1}^{{}^{\circ}}$
4851.292	3	20607.31	4*f* ^5^F_4_— 7*g* ${\;}^{3}G_{5}^{{}^{\circ}}$
4853.174	15*d*	20599.32	$\{\;\begin{array}{llll} 6p & {\;}^{3}P_{1}— & 6d & {\;}^{5}D_{1}^{{}^{\circ}}\\ 4f & {\;}^{5}F_{4}— & 7g & {\;}^{5}G_{4}^{{}^{\circ}} \end{array}$
4853.254	15	20598.98	4*f* ^5^F_4_— 7*g* ${\;}^{5}G_{5}^{{}^{\circ}}$
4859.150	1	20573.98	4*f* ^5^F_3_— 7*g* ${\;}^{5}G_{3}^{{}^{\circ}}$
4859.341	1	20573.18	4*f* ^5^F_3_— 7*g* ${\;}^{3}G_{4}^{{}^{\circ}}$
4859.786	15	20571.29	4*f* ^5^F_3_— 7*g* ${\;}^{5}G_{4}^{{}^{\circ}}$
4864.513	60	20551.30	$\{\;\begin{array}{llll} 6p^{\prime } & {\;}^{1}D_{2}— & 6d^{\prime } & 14_{3}^{{}^{\circ}}\\ 6p\prime \prime & {\;}^{3}P_{2}— & & 44_{2,3}^{{}^{\circ}} \end{array}$
4865.029	2	20549.12	7*p* ^3^P_1_— $25_{1,2}^{{}^{\circ}}$
4865.500	20	20547.13	4*f* ^5^F_5_— 7*g* ${\;}^{5}G_{6}^{{}^{\circ}}$
4867.649	20	20538.06	5*d*′ ${\;}^{3}D_{1}^{{}^{\circ}}$— 7*p* ^5^P_2_
4874.200	2	20510.45	4*f* ^5^F_2_— 7*g* ${\;}^{3}G_{3}^{{}^{\circ}}$
4874.473	2*d*	20509.31	4*f* ^5^F_2_— 7*g* ${\;}^{5}G_{2}^{{}^{\circ}}$
4876.051	10*c*	20502.67	4*f* ^5^F_2_— 7*g* ${\;}^{5}G_{3}^{{}^{\circ}}$
4881.185	4*h*	20481.10	
4882.127	40*c*	20477.15	$\{\;\begin{array}{llll} 5d^{\prime } & {\;}^{3}S_{1}^{{}^{\circ}}— & 6p\prime \prime & {\;}^{3}P_{2}\\ 6p^{\prime } & {\;}^{3}P_{1}— & 6d^{\prime } & 7_{1,2}^{{}^{\circ}} \end{array}$
4884.083	40*c*	20468.95	6*p*′ ^1^D_2_— 6*d*′ $13_{1,2}^{{}^{\circ}}$
4884.828	80*c, Z*	20465.83	6*s*′ ${\;}^{3}D_{2}^{{}^{\circ}}$— 6*p*′ ^1^F_3_
4886.182	5	20460.16	5*d*′ ${\;}^{3}D_{1}^{{}^{\circ}}$— 6*p*″ ^3^P_0_
4888.423	4	20450.78	4*f* ^5^F_1_— 7*g* ${\;}^{5}G_{2}^{{}^{\circ}}$
4891.224	2	20439.07	7*s* ${\;}^{3}S_{1}^{{}^{\circ}}$— 7*p*′ ^3^P_0_
4895.037	10*c*	20423.15	6*p*′ ^1^P_1_— 6*d*′ $4_{0}^{{}^{\circ}}$
4899.465	3	20404.69	5*d*′ ${\;}^{3}D_{1}^{{}^{\circ}}$— 7*p* ^5^P_1_
4908.755	25*c*	20366.07	$\{\;\begin{array}{llll} 7s & {\;}^{3}S_{1}^{{}^{\circ}}— & 7p^{\prime } & {\;}^{1}P_{1}\\ 6s\prime \prime & {\;}^{3}P_{2}^{{}^{\circ}}— & 6p\prime \prime & {\;}^{1}P_{1} \end{array}$
4910.579	10	20358.51	6*p*′ ^3^P_1_— 6*d*′ $6_{1,2}^{{}^{\circ}}$
4911.454	1	20354.88	
4920.587	6*c*	20317.10	6*p*′ ^3^D_3_— 7*d* ${\;}^{3}D_{3}^{{}^{\circ}}$
4924.546	40*c*	20300.77	6*s*″ ${\;}^{3}P_{1}^{{}^{\circ}}$— 7*p* ^3^P_2_
4930.049	6	20278.11	6*p*′ ^3^P_0_— 6*d*′ $5_{1}^{{}^{\circ}}$
4941.533	3*c*	20230.98	6*p*′ ^3^F_4_— 7*d* ${\;}^{3}D_{3}^{{}^{\circ}}$
4943.163	30*c*	20224.31	6*p*′ ^1^P_1_— 7*s*′ ${\;}^{1}D_{2}^{{}^{\circ}}$
4957.756	10	20164.78	6*p* ^3^P_2_— 5*d*″ ${\;}^{3}F_{2}^{{}^{\circ}}$
4959.992	1	20155.69	
4965.687	50*c*	20132.58	6*s*″ ${\;}^{3}P_{1}^{{}^{\circ}}$— 7*p* ^3^P_1_
4968.431	80	20121.46	6*s*′ ${\;}^{3}D_{2}^{{}^{\circ}}$— 6*p*′ ^1^P_1_
4980.773	1*c*	20071.60	6*p*″ ^3^D_2_— 7*g* ${\;}^{3}G_{3}^{{}^{\circ}}$
4981.960	1	20066.82	4*f* ^3^F_3_— 7*g* ${\;}^{3}G_{3}^{{}^{\circ}}$
4982.796	3	20063.45	
4984.083	15*c*	20058.27	4*f* ^3^F_3_— 7*g* ${\;}^{3}G_{4}^{{}^{\circ}}$
4984.265	5*c*	20057.54	6*p* ^3^P_1_— 6*d* ${\;}^{5}D_{0}^{{}^{\circ}}$
4984.562	3*c*	20056.34	4*f* ^3^F_3_— 7*g* ${\;}^{5}G_{4}^{{}^{\circ}}$
4985.351	2	20053.17	
4986.922	1000*Z*	20046.85	5*d* ${\;}^{3}D_{1}^{{}^{\circ}}$— 6*p*′ ^3^D_2_
4990.949	4	20030.68	7*s*′ ${\;}^{3}D_{2}^{{}^{\circ}}$— 13_2,3_
4992.223	50	20025.57	5*d*′ ${\;}^{1}D_{2}^{{}^{\circ}}$— 6*p*″ ^3^S_1_
4994.976	6*c*	20014.53	
5000.12	5	19993.94	6*p* ^5^P_2_— 5*d*″ ${\;}^{3}D_{2}^{{}^{\circ}}$
5002.02	5*c*	19986.35	
5008.36	100	19961.05	6*s*″ ${\;}^{3}P_{0}^{{}^{\circ}}$— 6*p*″ ^3^P_1_
5028.82	30*c*	19879.84	6*p*′ ^1^F_3_— 7*s*′ ${\;}^{1}D_{2}^{{}^{\circ}}$
5029.39	4	19877.58	5*d*″ ${\;}^{3}F_{3}^{{}^{\circ}}$— 6*f* ^5^F_4_
5029.67	15*c*	19876.48	6*p*′ ^3^P_2_— 7*d* ${\;}^{3}D_{3}^{{}^{\circ}}$
5032.15	6*c*	19866.68	6*p*′ ^1^F_3_— 6*d*′ $3_{3}^{{}^{\circ}}$
5040.23	15*w*	19834.83	6*p*″ ^3^D_3_— $39_{4}^{{}^{\circ}}$
5045.55	25	19813.92	4*f* ^3^F_4_— 7*g* ${\;}^{3}G_{5}^{{}^{\circ}}$
5046.43	150*Z*	19810.47	6*s*′ ${\;}^{3}D_{1}^{{}^{\circ}}$— 6*p*′ ^3^P_0_
5048.16	10*c*	19803.68	
5049.52	5*c*	19798.34	6*p*′ ^1^F_3_— 6*d*′ $2_{3}^{{}^{\circ}}$
5052.70	6	19785.88	5*d*′ ${\;}^{1}S_{0}^{{}^{\circ}}$— 6*p*″ ^1^P_1_
5057.00	3	19769.06	4*f* ^3^F_2_— 7*g* ${\;}^{3}G_{3}^{{}^{\circ}}$
5061.91	150	19749.88	6*s*″ ${\;}^{3}P_{0}^{{}^{\circ}}$— 7*p* ^5^P_1_
5063.37	15	19744.19	6*p*″ ^3^S_1_— $36_{2}^{{}^{\circ}}$
5065.37	400*c, Z*	19736.39	5*p*^5^ ${\;}^{3}P_{2}^{{}^{\circ}}$— 6*p* ^3^P_1_
5068.08	20*c*	19725.84	6*p*″ ^3^D_1_— 7*s*″ ${\;}^{3}P_{1}^{{}^{\circ}}$
5069.93	2	19718.64	
5092.60	30	19630.86	6*d* ${\;}^{3}D_{3}^{{}^{\circ}}$— 7*f* ^3^F_4_
5100.16	8	19601.77	5*p*^5^ ${\;}^{3}P_{0}^{{}^{\circ}}$— 6*p*′ ^3^D_1_
5110.36	8	19562.64	6*d* ${\;}^{3}D_{2}^{{}^{\circ}}$— 7*f* ^3^F_3_
5111.79	6	19557.17	6*d* ${\;}^{3}D_{1}^{{}^{\circ}}$— 7*f* ^3^F_2_
5114.56	20*c*	19546.58	6*p*″ ^3^D_1_— 7*s*″ ${\;}^{3}P_{0}^{{}^{\circ}}$
5115.89	2	19541.50	6*p*″ ^1^P_1_— $46_{2}^{{}^{\circ}}$
5124.57	40	19508.40	5*d* ${\;}^{3}D_{2}^{{}^{\circ}}$— 6*p*′ ^3^D_1_
5125.87	5	19503.45	7*p* ^5^P_2_— 9*d* ${\;}^{5}D_{3}^{{}^{\circ}}$
5126.58	2	19500.75	
5130.20	10*c*	19486.99	6*p*′ ^1^P_1_— 6*d*′ $1_{2}^{{}^{\circ}}$
5131.24	50	19483.04	6*s*″ ${\;}^{1}P_{1}^{{}^{\circ}}$— 6*p*″ ^1^D_2_
5135.79	100*d*	19465.78	5*d*′ ${\;}^{1}P_{1}^{{}^{\circ}}$— 6*p*″ ^1^S_0_
5143.01	10	19438.45	
5147.55	100*c*	19421.31	6*s*″ ${\;}^{3}P_{1}^{{}^{\circ}}$— 4*f* ^3^F_2_
5149.73	200	19413.09	5*d* ${\;}^{3}D_{3}^{{}^{\circ}}$— 6*p*′ ^3^F_3_
5154.97	50	19393.35	6*p*′ ^1^F_3_— 7*s*′ ${\;}^{3}D_{3}^{{}^{\circ}}$
5156.41	100*Z*	19387.94	6*p* ^5^P_1_— 7*s* ${\;}^{5}S_{2}^{{}^{\circ}}$
5161.20	3000*c, Z*	19360.94	6*s* ${\;}^{5}S_{2}^{{}^{\circ}}$— 6*p* ^5^P_3_
5174.70	50	19319.41	6*p*′ ^3^F_2_— 7*s*′ ${\;}^{3}D_{2}^{{}^{\circ}}$
5175.32	40*d*	19317.10	6*p*′ ^3^F_2_— 7*s*′ ${\;}^{3}D_{1}^{{}^{\circ}}$
5176.19	300	19313.85	6*s*″ ${\;}^{3}P_{2}^{{}^{\circ}}$— 6*p*″ ^3^P_2_
5179.22	12	19302.55	6*d* ${\;}^{5}D_{3}^{{}^{\circ}}$— 6*f* ^5^F_3_
5181.29	30	19294.84	6*d* ${\;}^{5}D_{3}^{{}^{\circ}}$— 6*f* ^5^F_4_
5183.21	10	19287.69	
5185.17	200	19280.40	6*p* ^5^P_2_— 7*s* ${\;}^{5}S_{2}^{{}^{\circ}}$
5188.62	20	19267.58	5*d*′ ${\;}^{3}S_{1}^{{}^{\circ}}$— 6*p*″ ^3^S_1_
5189.90	50	19262.83	6*d* ${\;}^{5}D_{4}^{{}^{\circ}}$— 6*f* ^5^F_5_
5190.06	20	19262.24	6*d* ${\;}^{5}D_{2}^{{}^{\circ}}$— 6*f* ^5^F_3_
5192.89	4*c*	19251.74	6*p*′ ^3^P_2_— 7*d* ${\;}^{3}D_{2}^{{}^{\circ}}$
5194.15	8	19247.07	6*d* ${\;}^{5}D_{4}^{{}^{\circ}}$— 6*f* ^5^F_4_
5198.89	6	19229.52	6*p*″ ^3^S_1_— $32_{1}^{{}^{\circ}}$
5205.48	20	19205.18	6*p* ^3^P_2_— 6*d* ${\;}^{5}D_{2}^{{}^{\circ}}$
5209.26	2*c*	19191.24	
5210.06	2*c*	19188.29	5*d*″ ${\;}^{3}F_{3}^{{}^{\circ}}$— 7*p*′ ^3^F_3_
5214.08	200*Z*	19173.50	5*d* ${\;}^{3}D_{3}^{{}^{\circ}}$— 6*p*′ ^3^D_2_
5216.27	600*Z*	19165.45	5*d* ${\;}^{3}D_{1}^{{}^{\circ}}$— 6*p*′ ^3^F_2_
5221.76	4*d*	19145.30	
5222.51	3*c*	19142.55	6*p*′ ^1^F_3_— 6*d*′ $1_{2}^{{}^{\circ}}$
5223.28	1*c*	19139.73	5*d*′ ${\;}^{3}F_{3}^{{}^{\circ}}$— 6*p*′ ^1^D_2_
5228.97	500*d*	19118.90	6*s*″ ${\;}^{3}P_{1}^{{}^{\circ}}$— 6*p*″ ^3^D_2_
5230.47	1*c*	19113.42	5*d*″ ${\;}^{3}D_{1}^{{}^{\circ}}$— 7*p*′ ^1^D_2_
5232.00	5	19107.83	7*p* ^5^P_3_— 9*d* ${\;}^{5}D_{4}^{{}^{\circ}}$?
5232.98	3	19104.25	7*p* ^5^P_3_— 9*d* ${\;}^{5}D_{3}^{{}^{\circ}}$
5241.64	2	19072.69	7*p* ^5^P_1_— 10*s* ${\;}^{5}S_{2}^{{}^{\circ}}$
5242.01	3	19071.34	
5243.50	1*c*	19065.92	
5245.71	3000*c, Z*	19057.89	6*s*′ ${\;}^{3}D_{3}^{{}^{\circ}}$— 6*p*′ ^3^P_2_
5256.19	1*c*	19019.89	
5259.34	2	19008.50	
5261.27	15*c*	19001.53	5*d*′ ${\;}^{3}D_{2}^{{}^{\circ}}$— 6*p*′ ^3^P_1_
5263.47	2	18993.59	5*d*″ ${\;}^{3}F_{3}^{{}^{\circ}}$— 7*p*′ ^1^D_2_
5265.16	150*c, Z*	18987.49	6*s*′ ${\;}^{3}D_{1}^{{}^{\circ}}$— 6*p*′ ^1^P_1_
5266.96	100*c*	18981.00	6*s*″ ${\;}^{3}P_{1}^{{}^{\circ}}$— 7*p* ^5^P_2_
5269.36	500	18972.36	6*s*″ ${\;}^{3}P_{2}^{{}^{\circ}}$— 6*p*″ ^3^D_3_
5270.58	2	18967.97	5*d*″ ${\;}^{3}F_{3}^{{}^{\circ}}$— 7*p*′ ^1^F_3_
5272.52	5	18960.99	5*d*″ ${\;}^{3}D_{1}^{{}^{\circ}}$— 4*f*′ ^3^D_1_
5278.57	4	18939.25	7*p* ^5^P_2_— 10*s* ${\;}^{5}S_{2}^{{}^{\circ}}$
5286.09	8*c*	18912.31	5*d*″ ${\;}^{3}F_{3}^{{}^{\circ}}$— 7*p*′ ^3^F_4_
5288.68	200*c*	18903.05	6*s*″ ${\;}^{3}P_{1}^{{}^{\circ}}$— 6*p*″ ^3^P_0_
5291.68	8	18892.33	5*d*′ ${\;}^{3}F_{2}^{{}^{\circ}}$— 7*p* ^3^P_2_
5293.65	4	18885.30	
5294.11	10	18883.66	6*p*″ ^3^P_2_— $37_{2}^{{}^{\circ}}$
5296.24	50	18876.07	6*p*″ ^3^D_3_— $36_{2}^{{}^{\circ}}$
5296.45	20	18875.32	6*p*′ ^3^P_1_— 7*d* ${\;}^{3}D_{2}^{{}^{\circ}}$
5299.78	400	18863.46	6*s*″ ${\;}^{1}P_{1}^{{}^{\circ}}$— 6*p*″ ^1^P_1_
5303.42	4	18850.51	6*d* ${\;}^{5}D_{1}^{{}^{\circ}}$— 6*f* ^5^F_2_?
5304.25	60*c*	18847.56	6*s*″ ${\;}^{3}P_{1}^{{}^{\circ}}$— 7*p* ^5^P_1_
5305.87	5*c*	18841.81	
5306.56	2	18839.36	
5307.63	10*c*	18835.56	5*d*″ ${\;}^{3}D_{1}^{{}^{\circ}}$— 7*p*′ ^3^F_2_
5309.44	20	18829.14	6*p*′ ^3^P_1_— 6*d*′ $5_{1}^{{}^{\circ}}$
5322.80	400*Z*	18781.88	5*p*^5^ ${\;}^{3}P_{1}^{{}^{\circ}}$— 6*p* ^3^P_0_
5323.83	80	18778.25	5*d*′ ${\;}^{3}G_{3}^{{}^{\circ}}$— 7*p* ^3^P_2_
5326.39	100*c*	18769.22	6*p*′ ^3^D_3_— 7*s*′ ${\;}^{1}D_{2}^{{}^{\circ}}$
5327.54	15	18765.17	5*d*″ ${\;}^{3}P_{2}^{{}^{\circ}}$— 8*p* ^3^P_2_
5330.13	8*c*	18756.05	6*p*′ ^3^D_3_— 6*d*′ $3_{3}^{{}^{\circ}}$
5337.13	15	18731.45	
5338.22	10000*Z*	18727.63	6*s*′ ${\;}^{3}D_{2}^{{}^{\circ}}$— 6*p*′ ^3^F_3_
5345.15	5000*c, Z*	18703.35	6*s*′ ${\;}^{3}D_{3}^{{}^{\circ}}$— 6*p*′ ^3^F_4_
5349.61	50*c*	18687.75	6*p*′ ^3^D_3_— 6*d*′ $2_{3}^{{}^{\circ}}$
5351.85	75*c*	18679.93	$\{\;\begin{array}{llll} 5p^{5} & {\;}^{1}P_{1}^{{}^{\circ}}— & 6p^{\prime } & {\;}^{3}P_{0}\\ 6p\prime \prime & {\;}^{1}S_{1}— & & 30_{1}^{{}^{\circ}} \end{array}$
5354.71	8*c*	18669.96	
5367.60	150*c*	18625.12	5*d*′ ${\;}^{3}D_{2}^{{}^{\circ}}$— 6*p*′ ^3^P_2_
5369.10	15*c*	18619.92	5*d*″ ${\;}^{3}F_{2}^{{}^{\circ}}$— 6*f* ^3^F_3_
5369.86	1000*c*	18617.28	6*s*′ ${\;}^{3}D_{3}^{{}^{\circ}}$— 6*p*′ ^3^D_3_
5372.49	30	18608.17	6*p* ^5^P_2_— 5*d*″ ${\;}^{3}P_{2}^{{}^{\circ}}$
5373.27	6*c*	18605.47	6*d* ${\;}^{5}D_{3}^{{}^{\circ}}$— 7*p*′ ^3^F_3_
5374.38	30*c*	18601.63	6*p*′ ^3^F_4_— 6*d*′ $2_{3}^{{}^{\circ}}$
5376.04	4	18595.88	
5378.99	3	18585.68	5*d*′ ${\;}^{3}P_{1}^{{}^{\circ}}$— 7*p*′ ^3^P_1_
5380.04	60	18582.06	6*p* ^3^P_2_— 5*d*″ ${\;}^{3}F_{3}^{{}^{\circ}}$
5386.23	20	18560.70	7*p* ^3^P_2_— 9*d* ${\;}^{3}D_{3}^{{}^{\circ}}$
5387.56	4*c*	18556.12	6*p*″ ^3^D_3_— $34_{2}^{{}^{\circ}}$
5392.27	12	18539.91	7*p* ^5^P_3_— 10*s* ${\;}^{5}S_{2}^{{}^{\circ}}$
5393.39	10	18536.06	
5393.86	8	18534.45	6*p*″ ^3^P_2_— $36_{2}^{{}^{\circ}}$
5399.53	3	18514.98	
5405.42	800*c, Z*	18494.81	5*p*^5^ ${\;}^{3}P_{2}^{{}^{\circ}}$— 6*p* ^5^P_3_
5407.36	800*c*	18488.17	6*s*′ ${\;}^{3}D_{2}^{{}^{\circ}}$— 6*p*′ ^3^D_2_
5414.96	15	18462.23	$\{\;\begin{array}{llll} 6p & {\;}^{3}P_{2}— & 5d\prime \prime & {\;}^{3}D_{1}^{{}^{\circ}}\\ 6p^{\prime } & {\;}^{3}P_{1}— & 7d & {\;}^{5}D_{1}^{{}^{\circ}} \end{array}$
5422.05	100	18438.08	6*p*′ ^3^D_2_— 7*s*′ ${\;}^{3}D_{2}^{{}^{\circ}}$
5422.74	100	18435.74	6*p*′ ^3^D_2_— 7*s*′ ${\;}^{3}D_{1}^{{}^{\circ}}$
5425.06	4*d*	18427.85	7*p* ^3^P_2_— 9*d* ${\;}^{3}D_{2}^{{}^{\circ}}$
5435.83	3000	18391.34	5*p*^5^ ${\;}^{3}P_{1}^{{}^{\circ}}$— 6*p* ^3^P_2_
5438.00	1000*Z*	18384.00	6*s*′ ${\;}^{1}D_{2}^{{}^{\circ}}$— 6*p*′ ^3^P_1_
5448.80	10	18347.57	7*s* ${\;}^{3}S_{1}^{{}^{\circ}}$— 4*f*′ ^3^P_0_
5454.43	8*d*	18328.63	6*p*′ ^3^P_2_— 7*s*′ ${\;}^{1}D_{2}^{{}^{\circ}}$
5457.06	200*c*	18319.79	$\{\;\begin{array}{llll} 6s\prime \prime & {\;}^{3}P_{0}^{{}^{\circ}}— & 6p\prime \prime & {\;}^{3}D_{1}\\ 5d\prime \prime & {\;}^{3}D_{2}^{{}^{\circ}}— & 4f^{\prime } & {\;}^{3}P_{2} \end{array}$
5458.36	15*c*	18315.43	6*p*′ ^3^P_2_— 6*d*′ $3_{3}^{{}^{\circ}}$
5464.62	2000*c*	18294.45	6*s* ${\;}^{5}S_{2}^{{}^{\circ}}$— 6*p* ^5^P_2_
5467.52	15	18284.75	6*p*′ ^3^P_1_— 7*d* ${\;}^{5}D_{2}^{{}^{\circ}}$
5468.12	50	18282.74	6*p*′ ^3^D_3_— 7*s*′ ${\;}^{3}D_{3}^{{}^{\circ}}$
5478.80	2*w*	18247.10	6*p*′ ^3^P_2_— 6*d*′ $2_{3}^{{}^{\circ}}$
5479.56	60	18244.57	6*p* ^3^P_1_— 7*s* ${\;}^{3}S_{1}^{{}^{\circ}}$
5480.84	10*c*	18240.31	6*p*″ ^3^P_0_— 7*s*″ ${\;}^{3}P_{1}^{{}^{\circ}}$
5491.50	800*Z*	18204.90	6*p* ^5^P_3_— 7*s* ${\;}^{5}S_{2}^{{}^{\circ}}$
5492.31	5	18202.22	
5493.43	400	18198.51	6*p*′ ^3^F_3_— 7*s*′ ${\;}^{3}D_{2}^{{}^{\circ}}$
5494.00	400*c*	18196.62	6*p*′ ^3^F_4_— 7*s*′ ${\;}^{3}D_{3}^{{}^{\circ}}$
5496.94	1000*c*	18186.89	6*s* ${\;}^{5}S_{2}^{{}^{\circ}}$— 6*p* ^5^P_1_
5497.65	300	18184.54	5*d*′ ${\;}^{3}D_{2}^{{}^{\circ}}$— 6*p*′ ^3^D_3_
5504.72	1000*Z*	18161.18	6*s* ${\;}^{3}S_{1}^{{}^{\circ}}$— 6*p* ^3^P_0_
5510.50	2	18142.13	
5517.23	2	18120.00	
5518.38	8	18116.23	7*p* ^5^P_1_— 7*s*″ ${\;}^{3}P_{0}^{{}^{\circ}}$
5522.06	600*c*	18104.16	6*s*″ ${\;}^{3}P_{2}^{{}^{\circ}}$— 6*p*″ ^3^S_1_
5523.60	8	18099.11	6*p*′ ^3^P_0_— 5*d*″ ${\;}^{3}P_{1}^{{}^{\circ}}$
5525.44	12	18093.08	7*s* ${\;}^{5}S_{2}^{{}^{\circ}}$*—* 8*p* ^3^P_2_
5525.92	5*c*	18091.51	
5527.88	6	18085.09	
5528.09 5528.19	$\}\;\begin{array}{c} 6\\ 2 \end{array}$	18084.41 18084.08	6*p*″ ^3^P_1_— 7*s*″ ${\;}^{3}P_{1}^{{}^{\circ}}$
5531.96	2	18071.76	6*p* ^3^P_0_— 5*d*″ ${\;}^{3}D_{1}^{{}^{\circ}}$
5538.08	6*c*	18051.79	$\{\;\begin{array}{llll} 5d^{\prime } & {\;}^{3}F_{3}^{{}^{\circ}}— & 6p\prime \prime & {\;}^{1}D_{2}\\ 5d\prime \prime & {\;}^{3}D_{2}^{{}^{\circ}}— & 7p^{\prime } & {\;}^{3}P_{2} \end{array}$
5541.52	4	18040.58	
5541.96	8	18039.15	5*d*″ ${\;}^{3}D_{2}^{{}^{\circ}}$*—* 7*p*′ ^3^D_3_
5544.17	10*c*	18031.96	6*p*′ ^3^D_3_— 6*d*′ $1_{2}^{{}^{\circ}}$
5546.55	30*w*	18024.22	6*p*″ ^3^D_2_— 7*s*″ ${\;}^{3}P_{1}^{{}^{\circ}}$
5550.08	50	18012.76	5*d*′ ${\;}^{3}F_{2}^{{}^{\circ}}$*—* 4*f* ^3^F_2_
5550.51	2	18011.36	6*d* ${\;}^{5}D_{1}^{{}^{\circ}}$— 7*p*′ ^1^P_1_
5551.65	400	18007.66	6*s*′ ${\;}^{1}D_{2}^{{}^{\circ}}$— 6*p*′ ^3^P_2_
5568.80	20*c*	17952.21	6*p*′ ^3^P_1_— 7*s*′ ${\;}^{1}D_{2}^{{}^{\circ}}$
5569.56	6	17949.76	5*d*″ ${\;}^{1}F_{3}^{{}^{\circ}}$— 6*f* ^3^F_4_
5570.60	2	17946.40	7*p* ^3^P_1_— 10*s* ${\;}^{3}S_{1}^{{}^{\circ}}$
5575.78	3	17929.73	
5583.44	25	17905.13	6*p*″ ^3^P_1_— 7*s*″ ${\;}^{3}P_{0}^{{}^{\circ}}$
5584.04	20	17903.21	
5585.48	4	17898.59	5*d*′ ${\;}^{3}G_{3}^{{}^{\circ}}$— 4*f* ^3^F_2_
5587.88	4	17890.91	5*d*″ ${\;}^{1}F_{3}^{{}^{\circ}}$— 6*f* ^3^F_2_
5589.29	80	17886.39	5*d*′ ${\;}^{3}S_{1}^{{}^{\circ}}$— 7*p* ^3^P_0_
5590.20	8	17883.48	6*p*′ ^3^D_1_— 5*d*″ ${\;}^{1}P_{1}^{{}^{\circ}}$
5593.12	300*Z*	17874.15	5*d* ${\;}^{3}D_{1}^{{}^{\circ}}$— 6*p*′ ^3^D_1_
5595.47	4*w*	17866.64	5*d*′ ${\;}^{1}D_{2}^{{}^{\circ}}$— 7*p* ^3^P_2_
5598.52	600*c*	17856.91	5*p*^5^ ${\;}^{1}P_{1}^{{}^{\circ}}$— 6*p*′ ^1^P_1_
5600.32	1000	17851.17	5*d*′ ${\;}^{3}G_{3}^{{}^{\circ}}$— 4*f* ^3^F_4_
5601.94	4	17846.00	
5603.16	80	17842.12	6*p*′ ^3^P_2_— 7*s*′ ${\;}^{3}D_{3}^{{}^{\circ}}$
5606.52	15	17831.43	6*p*″ ^1^P_1_— $37_{2}^{{}^{\circ}}$
5608.41	4	17825.42	
5612.89	1500	17811.19	$\{\;\begin{array}{llll} 6s\prime \prime & {\;}^{1}P_{1}^{{}^{\circ}}— & 6p\prime \prime & {\;}^{3}P_{2}\\ 6p\prime \prime & {\;}^{3}D_{3}— & & 30_{2}^{{}^{\circ}} \end{array}$
5616.77	10	17798.89	
5618.28	6	17794.10	
5618.57	5	17793.18	6*d* ${\;}^{5}D_{1}^{{}^{\circ}}$— 4*f*′ ^3^D_1_
5620.02	10*c*	17788.59	5*d*″ ${\;}^{3}D_{3}^{{}^{\circ}}$— 6*p*″ ^1^D_2_
5623.30	12	17778.22	7*p* ^3^P_2_— 10*s* ${\;}^{3}S_{1}^{{}^{\circ}}$
5625.69	10000*Z*	17770.67	6*s* ${\;}^{3}S_{1}^{{}^{\circ}}$— 6*p* ^3^P_2_
5638.91	1	17729.00	6*p*″ ^3^S_1_— $21_{1}^{{}^{\circ}}$
5639.78	1	17726.27	
5641.10	20*c*	17722.12	4*f* ^3^F_2_— 7*s*″ ${\;}^{3}P_{1}^{{}^{\circ}}$
5643.32	100	17715.15	5*d*′ ${\;}^{3}F_{2}^{{}^{\circ}}$— 4*f* ^3^F_3_
5643.67	5*c*	17714.05	5*d*″ ${\;}^{3}F_{2}^{{}^{\circ}}$— 4*f*′ ^1^D_2_
5648.66	15*c*	17698.40	5*d*′ ${\;}^{1}D_{2}^{{}^{\circ}}$— 7*p* ^3^P_1_
5649.06	2	17697.15	
5655.10	10	17678.25	
5655.52	5*w*	17676.93	6*p* ^3^P_1_— 5*d*″ ${\;}^{3}D_{2}^{{}^{\circ}}$
5658.46	8*c*	17667.75	6*d* ${\;}^{5}D_{1}^{{}^{\circ}}$— 7*p*′ *^3^*F_2_
5663.63	25	17651.62	5*d*′ ${\;}^{3}P_{1}^{{}^{\circ}}$— 5*f* ^3^F_2_
5665.71	6	17645.14	
5676.70	7	17610.98	
5678.08	1000*Z*	17606.70	6*s*′ ${\;}^{3}D_{2}^{{}^{\circ}}$— 6*p*′ ^3^F_2_
5678.88	1*c*	17604.22	
5679.92	50	17601.00	5*d*′ ${\;}^{3}G_{3}^{{}^{\circ}}$— 4*f* ^3^F_3_
5682.64	15	17592.57	
5683.83	2	17588.89	6*p*′ ^3^P_2_— 8*s* ${\;}^{3}S_{1}^{{}^{\circ}}$
5688.21	30	17575.35	
5690.91	2000*c*, *Z*	17567.01	6*s*′ ${\;}^{1}D_{2}^{{}^{\circ}}$— 6*p*′ ^3^D_3_
5692.68	10	17561.55	7*s* ${\;}^{5}S_{2}^{{}^{\circ}}$— 4*f*′ ^3^D_3_
5698.97	2	17542.16	
5702.05	500*Z*	17532.69	5*d*′ ${\;}^{3}D_{3}^{{}^{\circ}}$— 6*p*′ ^3^P_2_
5704.87	20	17524.02	5*d*′ ${\;}^{1}S_{0}^{{}^{\circ}}$— 6*p*″ ^3^S_1_
5710.53	4000*c*, *Z*	17506.65	6*s*′ ${\;}^{3}D_{3}^{{}^{\circ}}$— 6*p*′ ^1^F_3_
5715.57	40	17491.22	
5716.34	3	17488.86	
5717.58	1*c*	17485.07	
5717.85	5	17484.24	7*s* ${\;}^{3}S_{1}^{{}^{\circ}}$— 7*p*′ ^3^P_2_
5726.76	15	17457.04	
5733.46	20	17436.64	7*s* ${\;}^{5}S_{2}^{{}^{\circ}}$— 8*p* ^5^P_2_?
5735.69	12	17429.86	
5735.87	15	17429.31	
5738.27	1000*Z*	17422.02	5*p*^5^ ${\;}^{3}P_{1}^{{}^{\circ}}$— 6*p* ^3^P_1_
5738.83	40	17420.32	} 5*p*^5^ ${\;}^{3}P_{2}^{{}^{\circ}}$— 6*p* ^5^P_2_
5738.89	70	17420.14	
5739.01	100	17419.78	
5739.18	150	17419.26	
5739.40	200	17418.59	
5739.59	150	17418.02	} 6*s*″ ${\;}^{3}P_{1}^{{}^{\circ}}$—6*p*″ ^3^D_1_
5739.77	200	17417.47	
5739.88	70	17417.14	
5739.95	40	17416.92	
5741.94	3	17410.89	5*d*″ ${\;}^{3}F_{2}^{{}^{\circ}}$— 7*p*′ ^1^D_2_
5743.06	30	17407.49	
5749.04	5	17389.39	
5750.41	50	17385.24	5*d*″ ${\;}^{3}F_{2}^{{}^{\circ}}$— 7*p*′ ^1^F_3_
5752.35	25*c*	17379.38	5*d*″ ${\;}^{3}D_{2}^{{}^{\circ}}$— 8*p* ^3^P_2_
5757.22	6*c*	17364.68	
5760.72	1000*Z*	17354.13	6*s*′ ${\;}^{3}D_{1}^{{}^{\circ}}$— 6*p*′ ^3^D_2_
5763.14	25	17346.84	4*f* ^5^F_4_— 6*g* ${\;}^{3}G_{5}^{{}^{\circ}}$
5765.43	3*c*	17339.95	
5765.68	8	17339.20	$\{\;\begin{array}{cccc} 4f & {\;}^{5}F_{4}- & 6g & {\;}^{3}G_{4}^{{}^{\circ}}\\ 4f & {\;}^{5}F_{4}- & 6g & {\;}^{5}G_{3}^{{}^{\circ}} \end{array}$
5767.19	100	17334.66	4*f* ^5^F_4_— 6*g* ${\;}^{5}G_{5}^{{}^{\circ}}$
5769.01	8	17329.19	
5770.46	3*h*	17324.84	7*s*′ ${\;}^{3}D_{2}^{{}^{\circ}}$— 3_2,3_
5771.02	10	17323.16	4*f* ^5^F_3_— 6*g* ${\;}^{3}G_{3}^{{}^{\circ}}$
5774.83	500*c*	17311.73	5*p*^5^ ${\;}^{3}P_{2}^{{}^{\circ}}$— 6*p* ^5^P_1_
5776.54	50	17306.60	4*f* ^5^F_3_— 6*g* ${\;}^{5}G_{4}^{{}^{\circ}}$
5783.34	100	17286.25	4*f* ^5^F_5_— 6*g* ${\;}^{5}G_{6}^{{}^{\circ}}$
5787.02	500	17275.26	6*p* ^3^P_2_— 7*s* ${\;}^{3}S_{1}^{{}^{\circ}}$
5790.26	10	17265.59	
5794.85	25	17251.92	4*f* ^5^F_2_— 6*g* ${\;}^{3}G_{3}^{{}^{\circ}}$
5795.30	10	17250.58	4*f* ^3^F_2_— 6*g* ${\;}^{5}G_{2}^{{}^{\circ}}$
5797.02	50	17245.46	
5798.95^b^	100	17239.72	4*f* ^5^F_2_— 6*g* ${\;}^{5}G_{3}^{{}^{\circ}}$
5807.29	15	17214.96	6*p*′ ^3^P_1_— 6*d*′ $1_{2}^{{}^{\circ}}$
5808.21	3	17212.24	6*p*′ ^3^P_1_— 8*s* ${\;}^{3}S_{1}^{{}^{\circ}}$
5808.38	5	17211.73	6*p*″ ^1^D_2_— $37_{2}^{{}^{\circ}}$
5812.31	4*c*	17200.09	5*d*′ ${\;}^{3}F_{2}^{{}^{\circ}}$— 4*f* ^5^F_3_
5815.06	40	17191.96	4*f* ^5^F_1_— 6*g* ${\;}^{5}G_{2}^{{}^{\circ}}$
5819.71	80*c*	17178.22	5*d*′ ${\;}^{3}D_{3}^{{}^{\circ}}$— 6*p*′ ^3^F_4_
5821.97	30*w*	17171.56	
5823.54	3*c*	17166.93	
5823.71	10	17166.43	
5824.19	20	17165.01	
5825.69	6	17160.59	5*d*″ ${\;}^{3}D_{1}^{{}^{\circ}}$— 4*f*′ ^3^P_0_
5827.74	4	17154.55	
5830.79	4*c*	17145.58	
5832.86	15	17139.50	
5834.78	8*c*	17133.86	
5835.06	10	17133.04	5*d*″ ${\;}^{3}F_{2}^{{}^{\circ}}$— 7*p*′ ^3^F_2_
5838.57	8	17122.74	
5839.88	2	17118.89	
5843.38	50	17108.64	5*d*′ ${\;}^{3}S_{1}^{{}^{\circ}}$— 7*p* ^3^P_2_
5845.04	6	17103.78	
5849.03	20	17092.11	5*d*′ ${\;}^{3}D_{3}^{{}^{\circ}}$— 6*p*′^3^D_3_
5851.76	20	17084.14	
5855.26	10*d*	17073.93	5*d*′ ${\;}^{3}D_{2}^{{}^{\circ}}$— 6*p*′ ^1^F_3_
5863.07	5	17051.19	
5868.64	5	17035.00	
5873.12	3	17022.01	
5893.06	20	16964.41	
5899.47	8*c*	16945.98	5*d*′ ${\;}^{1}D_{2}^{{}^{\circ}}$— 7*p* ^5^P_3_
5901.41	60	16940.41	5*d*′ ${\;}^{3}S_{1}^{{}^{\circ}}$— 7*p* ^3^P_1_
5906.04	10	16927.13	
5910.42	20	16914.58	
5911.18	20	16912.41	5*d*″ ${\;}^{1}F_{3}^{{}^{\circ}}$— 4*f*′ ^1^D_2_
5911.78	12	16910.69	
5911.96	60	16910.18	
5913.40	10	16906.06	
5913.67	50	16905.29	
5918.10	12	16892.63	
5920.59	200*Z*	16885.53	5*d* ${\;}^{5}D_{3}^{{}^{\circ}}$— 6*p* ^3^P_2_
5920.87	40	16884.73	6*p* ^3^P_0_— 7*s* ${\;}^{3}S_{1}^{{}^{\circ}}$
5928.63	20	16862.63	6*p*″ ^1^D_2_***—*** $36_{2}^{{}^{\circ}}$
5934.96	10	16844.65	
5938.35	10	16835.03	
5939.69^c^	100	16831.23	7*p* ^3^P_1_— 7*s*″ ${\;}^{3}P_{0}^{{}^{\circ}}$
5946.15	15*c*	16812.95	6*p*″ ^3^D_2_— 6*g* ${\;}^{3}G_{3}^{{}^{\circ}}$
5947.79	5	16808.31	4*f* ^3^F_3_— 6*g* ${\;}^{3}G_{3}^{{}^{\circ}}$
5950.25	5000*Z*	16801.36	6*s* ${\;}^{3}S_{1}^{{}^{\circ}}$— 6*p* ^3^P_1_
5952.09	50	16796.17	$\{\;\begin{array}{cccc} 4f & {\;}^{3}F_{3}- & 6g & {\;}^{3}G_{4}^{{}^{\circ}}\\ 4f & {\;}^{3}F_{3}- & 6g & {\;}^{5}G_{3}^{{}^{\circ}} \end{array}$
5953.66	20	16791.74	4*f* ^3^F_3_— 6*g* ${\;}^{5}G_{4}^{{}^{\circ}}$
5975.83	25*w*	16729.44	5*d*′ ${\;}^{3}D_{2}^{{}^{\circ}}$— 6*p*′ ^1^P_1_
5983.69	6*c*	16707.47	6*p* ^3^P_2_— 5*d*″ ${\;}^{3}D_{2}^{{}^{\circ}}$
5986.27	3	16700.27	
5988.23	12	16694.80	
5990.17	20*c*	16689.39	5*d*′ ${\;}^{1}D_{2}^{{}^{\circ}}$— 4*f* ^3^F_3_
5991.86	30	16684.69	5*d*′ ${\;}^{1}D_{2}^{{}^{\circ}}$— 6*p*″ ^3^D_2_
6008.19	15	16639.34	
6013.55	7*c*	16624.51	5*d*′ ${\;}^{1}D_{2}^{{}^{\circ}}$— 6*p*″ ^3^P_1_
6021.88	50	16601.51	6*s*″ ${\;}^{1}P_{1}^{{}^{\circ}}$— 6*p*″ ^3^S_1_
6028.43	10	16583.47	5*d*″ ${\;}^{1}F_{3}^{{}^{\circ}}$— 7*p*′ ^1^F_3_
6031.41	8*c*	16575.28	5*f* ^3^F_3_— $50_{3,4}^{{}^{\circ}}$
6032.80	20*w*	16571.46	
6033.44	10	16569.70	7*p* ^5^P_1_— 8*d* ${\;}^{3}D_{1}^{{}^{\circ}}$
6035.13	20	16565.06	5*d*″ ${\;}^{3}D_{1}^{{}^{\circ}}$— 4*f*′ ^3^P_2_
6036.15	3*c*	16562.27	
6039.32	100	16553.57	4*f* ^3^F_4_— 6*g* ${\;}^{3}G_{5}^{{}^{\circ}}$
6041.60	100	16547.33	5*d*′ ${\;}^{1}P_{1}^{{}^{\circ}}$*—* 6*p*″ ^1^D_2_
6043.79	15	16541.33	4*f* ^3^F_4_— 6*g* ${\;}^{5}G_{5}^{{}^{\circ}}$
6048.70	50	16527.90	5*d*″ ${\;}^{1}F_{3}^{{}^{\circ}}$— 7*p*′ ^3^F_4_
6051.82	10*c*	16519.38	6*p*″ ^3^P_2_— $21_{1}^{{}^{\circ}}$
6055.06^d^	50*d*	16510.54	4*f* ^3^F_2_— 6*g* ${\;}^{3}G_{3}^{{}^{\circ}}$
6059.53	8*c*	16498.36	4*f* ^3^F_2_— 6*g* ${\;}^{5}G_{3}^{{}^{\circ}}$
6068.93	500*Z*	16472.81	6*s*′ ${\;}^{3}D_{1}^{{}^{\circ}}$— 6*p*′ ^3^F_2_
6074.98	2000*c*, *Z*	16456.40	6*s*′ ${\;}^{1}D_{2}^{{}^{\circ}}$— 6*p*′ ^1^F_3_
6077.83	150	16448.69	5*d* ${\;}^{5}D_{2}^{{}^{\circ}}$— 6*p* ^3^P_2_
6084.78	30	16429.90	6*p* ^3^P_1_— 5*d*′ ${\;}^{3}P_{1}^{{}^{\circ}}$
6090.90	12*c*	16413.39	5*d*′ ${\;}^{1}D_{2}^{{}^{\circ}}$— 7*p* ^5^P_1_
6094.08	20*c*	16404.83	$\{\;\begin{array}{cccc} 5d^{\prime \prime } & {\;}^{1}D_{2}^{{}^{\circ}}- & 6p^{\prime \prime } & {\;}^{3}P_{2}\\ 5d^{\prime \prime } & {\;}^{3}D_{2}^{{}^{\circ}}- & 5f & {\;}^{3}F_{2} \end{array}$
6094.33	15	16404.15	
6103.37	150	16379.86	5*d*′ ${\;}^{1}F_{3}^{{}^{\circ}}$— 6*p*″ ^3^P_2_
6103.87	15	16378.52	
6111.35	6	16358.47	6*p*″ ^3^P_1_— 8*d* ${\;}^{3}D_{1}^{{}^{\circ}}$
6127.49	2000*c*, *Z*	16315.38	6*s*′ ${\;}^{3}D_{2}^{{}^{\circ}}$— 6*p*′ ^3^D_1_
6132.86	20	16301.09	6*p*″ ^3^P_1_— 8*d* ${\;}^{3}D_{2}^{{}^{\circ}}$
6137.39	20	16289.06	5*d*′ ${\;}^{3}P_{1}^{{}^{\circ}}$— 6*p*″ ^1^S_0_
6148.15	40*d*	16260.56	
6148.34	50*Z*	16260.05	5*d* ${\;}^{5}D_{0}^{{}^{\circ}}$— 6*p* ^3^P_1_
6155.36	200	16241.51	6*d* ${\;}^{3}D_{3}^{{}^{\circ}}$— 6*f* ^3^F_4_
6155.97	6	16239.90	
6156.77	12	16237.79	
6157.14	15	16236.81	
6158.18	20	16234.07	
6160.09	15*c*	16229.04	5*d*′ ${\;}^{3}S_{1}^{{}^{\circ}}$— 4*f* ^3^F_2_
6162.22	300*c*	16223.43	$\{\;\begin{array}{cccc} 5p^{5} & {\;}^{1}P_{1}^{{}^{\circ}}- & 6p^{\prime } & {\;}^{3}D_{2}\\ 6d & {\;}^{3}D_{2}^{{}^{\circ}}- & 6f & {\;}^{3}F_{2} \end{array}$
6164.88	8*c*	16216.43	
6166.44	40*c*	16212.33	
6172.84	4	16195.52	6*p*″ ^1^P_1_— $29_{1}^{{}^{\circ}}$
6182.28	60	16170.79	
6184.62	10	16164.67	5*d*″ ${\;}^{3}F_{3}^{{}^{\circ}}$— 7*p*′ ^3^D_3_
6186.93	3	16158.63	4*f* ^5^F_3_— 8*d* ${\;}^{5}D_{2}^{{}^{\circ}}$
6189.94	60	16150.78	$\{\;\begin{array}{cccc} 6d & {\;}^{3}D_{2}^{{}^{\circ}}- & 6f & {\;}^{3}F_{3}\\ 7p & {\;}^{5}P_{3}- & 8d & {\;}^{3}D_{3}^{{}^{\circ}} \end{array}$
6197.16	50*c*	16131.96	6*d* ${\;}^{3}D_{1}^{{}^{\circ}}$— 6*f* ^3^F_2_
6201.69	2	16120.18	
6203.04	20*c*	16116.67	5*d*″ ${\;}^{3}D_{3}^{{}^{\circ}}$— 6*p*″ ^3^P_2_
6204.86	1000*Z*	16111.94	6*s*′ ${\;}^{1}D_{2}^{{}^{\circ}}$— 6*p*′ ^1^P_1_
6205.61	5	16109.99	6*d* ${\;}^{3}D_{3}^{{}^{\circ}}$— 6*f* ^3^F_3_
6222.93	3	16065.16	6*p*″ ^3^P_2_— $20_{1,2}^{{}^{\circ}}$
6223.15	2	16064.59	7*p* ^2^P_0_— 7*s*″ ${\;}^{3}P_{1}^{{}^{\circ}}$
6223.66	3	16063.27	5*d*″ ${\;}^{1}D_{2}^{{}^{\circ}}$— 6*p*″ ^3^D_3_
6225.07	1	16059.63	5*f* ^5^F_5_*—* 12*g* ${\;}^{5}G_{6}^{{}^{\circ}}$
6238.58	5*c*	16024.86	
6239.89	2*d*	16021.49	5*d*″ ${\;}^{3}D_{2}^{{}^{\circ}}$— 5*f* ^3^F_3_
6250.59	40	15994.07	6*p* ^3^P_2_— 7*S* ${\;}^{5}S_{2}^{{}^{\circ}}$
6255.53	500*c*	15981.44	5*d*′ ${\;}^{3}D_{3}^{{}^{\circ}}$— 6*p*′ ^1^F_3_
6256.33	40	15979.39	6*p*′ ^3^P_0_— 7*s*′ ${\;}^{3}D_{1}^{{}^{\circ}}$
6257.49	900*c*	15976.43	5*d*′ ${\;}^{3}F_{4}^{{}^{\circ}}$— 6*p*′ ^3^F_4_
6269.70	1*c*	15945.32	6*s*″ ${\;}^{3}P_{2}^{{}^{\circ}}$— 7*p* ^3^P_2_
6272.37	3*c*	15938.53	5*d*″ ${\;}^{3}D_{1}^{{}^{\circ}}$— 4*f*′ ^3^D_2_
6273.16	15	15936.52	7*p* ^5^P_1_— 8*d* ${\;}^{5}D_{1}^{{}^{\circ}}$
6276.65	40*c*	15927.66	
6277.05	30*c*	15926.65	5*d*′ ${\;}^{3}S_{1}^{{}^{\circ}}$— 6*p*″ ^3^D_2_
6279.79	12	15919.70	7*p* ^5^P_1_— 8*d* ${\;}^{5}D_{2}^{{}^{\circ}}$
6280.36	80*c*	15918.25	5*d*′ ${\;}^{3}P_{0}^{{}^{\circ}}$— 6*p*′ ^1^P_1_
6282.61	20	15912.55	7*p* ^5^P_1_— 8*d* ${\;}^{5}D_{0}^{{}^{\circ}}$
6291.39	500	15890.34	5*d*′ ${\;}^{3}F_{4}^{{}^{\circ}}$— 6*p*′ ^3^D_3_
6300.47	3	15867.44	
6300.84	15	15866.51	5*d*′ ${\;}^{3}S_{1}^{{}^{\circ}}$— 6*p*″ ^3^P_1_
6320.41	3*h*	15817.38	5*d*″ ${\;}^{3}D_{2}^{{}^{\circ}}$— 5*f* ^5^F_3_
6326.10	4	15803.16	7*p* ^5^P_2_— 8*d* ${\;}^{5}D_{1}^{{}^{\circ}}$
6326.45	2*c*	15802.28	
6329.38	40	15794.97	7*p* ^5^P_2_— 8*d* ${\;}^{5}D_{3}^{{}^{\circ}}$
6331.87	30*w*	15788.76	5*d*′ ${\;}^{3}S_{1}^{{}^{\circ}}$— 7*p* ^5^P_2_
6332.84	25	15786.34	7*p* ^5^P_2_— 8*d* ${\;}^{5}D_{2}^{{}^{\circ}}$
6336.57	100*c*	15777.05	6*s*″ ${\;}^{3}P_{2}^{{}^{\circ}}$— 7*p* ^3^P_1_
6339.97	140*c*	15768.59	6*s*′ ${\;}^{3}D_{3}^{{}^{\circ}}$— 6*p*′ ^3^F_3_
6341.24	100*c*	15765.43	5*d*′ ${\;}^{3}F_{3}^{{}^{\circ}}$— 6*p*′ ^3^P_2_
6347.77	4	15749.21	
6357.37	6	15725.43	6*p*″ ^3^P_1_— 8*d* ${\;}^{5}D_{1}^{{}^{\circ}}$
6363.27	50	15710.85	$\{\;\begin{array}{cccc} 6p^{\prime } & {\;}^{3}D_{2}- & 5d^{\prime \prime } & {\;}^{1}P_{1}^{{}^{\circ}}\\ 5d^{\prime } & {\;}^{3}S_{1}^{{}^{\circ}}- & 6p^{\prime \prime } & {\;}^{3}P_{0} \end{array}$
6364.23	8	15708.48	6*p*″ ^3^P_1_— 8*d* ${\;}^{5}D_{2}^{{}^{\circ}}$
6385.14	5*c*	15657.04	6*p*″ ^3^D_2_— 8*d* ${\;}^{5}D_{3}^{{}^{\circ}}$
6385.46	8	15656.25	6*p*″ ^1^P_1_— $24_{1,2}^{{}^{\circ}}$
6385.80	30	15655.42	5*d*′ ${\;}^{3}S_{1}^{{}^{\circ}}$— 7*p* ^5^P_1_
6387.38	5	15651.55	
6388.66	5*c*	15648.41	6*p*″ ^3^D_2_— 8*d* ${\;}^{5}D_{2}^{{}^{\circ}}$
6389.60	7	15646.11	
6393.58	3*h*	15636.37	
6395.33	15	15632.09	
6420.96	10	15569.69	
6421.12	20	15569.30	
6430.42	12	15546.79	
6440.46	400*c*	15522.55	
6458.41	30	15479.41	5*d* ${\;}^{5}D_{2}^{{}^{\circ}}$— 6*p* ^3^P_1_
6459.46	2	15476.89	5*f* ^5^F_5_— 11*g* ${\;}^{5}G_{6}^{{}^{\circ}}$
6464.79	30*w*	15464.13	6*p*′ ^1^D_2_— 7*d* ${\;}^{5}D_{1}^{{}^{\circ}}$
6466.42	4*h*	15460.24	6*p*″ ^3^S_1_—10*s* ${\;}^{5}S_{2}^{{}^{\circ}}$
6470.25	8	15451.08	5*d*″ ${\;}^{3}D_{1}^{{}^{\circ}}$— 4*f*′ ^3^F_2_
6488.97	50	15406.51	7*p* ^5^P_3_— 8*d* ${\;}^{5}D_{4}^{{}^{\circ}}$
6492.86	6	15397.28	6*d* ${\;}^{5}D_{1}^{{}^{\circ}}$— 4*f*′ ^3^P_2_
6493.49	20	15395.79	7*p* ^5^P_3_— 8*d* ${\;}^{5}D_{3}^{{}^{\circ}}$
6497.12	4	15387.18	7*p* ^5^P_3_— 8*d* ${\;}^{5}D_{2}^{{}^{\circ}}$
6509.39	3*c*	15358.18	
6512.98	6*c*	15349.71	6*p*′ ^3^D_3_— 7*S*′ ${\;}^{3}D_{2}^{{}^{\circ}}$
6516.18	250*c*, *Z*	15342.18	5*p*^5^ ${\;}^{1}P_{1}^{{}^{\circ}}$— 6*p*′ ^3^F_2_
6518.91	12*c*	15335.75	
6520.26	5	15332.58	5*d*″ ${\;}^{3}D_{1}^{{}^{\circ}}$— 8*p* ^3^P_1_
6523.60	20*c*	15324.73	5*d*′ ${\;}^{3}F_{3}^{{}^{\circ}}$— 6*p*′ ^3^D_3_
6525.93	3	15319.25	7*d* ${\;}^{3}D_{2}^{{}^{\circ}}$— 12_1,2_
6526.76	10	15317.31	
6538.32	20	15290.23	5*d*′ ${\;}^{3}P_{2}^{{}^{\circ}}$— 6*p*″ ^3^P_2_
6539.96	2	15286.39	
6540.73	12	15284.59	5*p* ^3^P_1_— 8*d* ${\;}^{3}D_{1}^{{}^{\circ}}$
6544.01	10*c*	15276.93	
6545.60	10	15273.22	
6546.97	20*c*	15270.02	5*d* ${\;}^{5}D_{1}^{{}^{\circ}}$— 6*p* ^3^P_0_
6555.68	20	15249.74	
6564.14	40	15230.08	7*p* ^3^P_2_— 8*d* ${\;}^{3}D_{3}^{{}^{\circ}}$
6565.37	15	15227.23	7*p* ^3^P_1_— 8*d* ${\;}^{3}D_{2}^{{}^{\circ}}$
6567.76	2	15221.69	6*d* ${\;}^{3}D_{1}^{{}^{\circ}}$— 4*f*′ ^1^P_1_
6567.98	2	15221.18	
6576.04	3	15202.52	
6578.50	10	15196.84	5*d*′ ${\;}^{1}S_{0}^{{}^{\circ}}$— 7 *p* ^3^P_1_
6585.21	400	15181.35	6*s*′ ${\;}^{3}D_{1}^{{}^{\circ}}$— 6*p*′ ^3^D_1_
6613.82	4	15115.68	4*f*′ ^3^H_4_— $50_{3,4}^{{}^{\circ}}$
6618.51	4	15104.97	5*p^5^* ${\;}^{3}P_{1}^{{}^{\circ}}$— 6*p* ^5^P_2_
6621.71	12	15097.67	
6622.35	120*c*	15096.21	5*d*′ ${\;}^{3}D_{2}^{{}^{\circ}}$— 6*p*′ ^3^D_2_
6633.86	20	15070.02	6*p* ^3^P_0_— 5*d*′ ${\;}^{3}P_{1}^{{}^{\circ}}$
6638.72	15	15058.99	7*p* ^3^P_2_— 8*d* ${\;}^{3}D_{2}^{{}^{\circ}}$
6646.73	3	15040.84	
6659.49	20	15012.02	
6665.96	600*Z*	14997.45	5*p*^5^ ${\;}^{3}P_{1}^{{}^{\circ}}$— 6*p* ^5^P_1_
6672.27	100*c*	14983.27	5*d*′ ${\;}^{1}D_{2}^{{}^{\circ}}$— 6*p*″ ^3^D_1_
6679.77	6*c*	14966.44	
6685.20	60*c*	14954.29	6*p*′ ^1^D_2_— 7*s*′ ${\;}^{1}D_{2}^{{}^{\circ}}$
6687.71	40	14948.67	5*d*′ ${\;}^{3}P_{2}^{{}^{\circ}}$— 6p″ ^3^D_3_
6690.84	6	14941.68	6*d* ${\;}^{3}D_{2}^{{}^{\circ}}$— 7*p*′ ^1^D_2_
6705.45	10*c*	14909.13	6*p*′ ^3^P_2_— 7*s*′ ${\;}^{3}D_{2}^{{}^{\circ}}$
6708.73	5*c*	14901.84	
6709.12	3*c*	14900.97	6*d* ${\;}^{3}D_{3}^{{}^{\circ}}$— 7*p*′ ^1^D_2_
6711.79	1	14895.04	
6712.03	8	14894.51	5*d*″ ${\;}^{3}F_{3}^{{}^{\circ}}$— 4*f*′ ^3^G_4_
6717.05	80	14883.38	
6717.74	10	14881.85	6*d* ${\;}^{5}D_{2}^{{}^{\circ}}$— 8*p* ^3^P_2_
6718.83	300*Z*	14879.44	5*d* ${\;}^{5}D_{1}^{{}^{\circ}}$— 6*p* ^3^P_2_
6720.66	100	14875.30	$\{\;\begin{array}{cccc} 5d^{\prime } & {\;}^{1}P_{1}^{{}^{\circ}}- & 6p^{\prime \prime } & {\;}^{3}P_{2}\\ 6d & {\;}^{3}D_{3}^{{}^{\circ}}- & 7p^{\prime } & {\;}^{1}F_{3} \end{array}$
6721.43	20	14873.68	7*p* ^5^P_2_— 9*s* ${\;}^{5}S_{2}^{{}^{\circ}}$
6725.77	4*c*	14864.08	
6739.92	35	14832.88	
6744.30	5*h*	14823.24	
6745.90	6*c*	14819.73	6*d* ${\;}^{3}D_{3}^{{}^{\circ}}$— 7*p*′ ^3^F_4_
6756.74	6	14795.95	6*p*″ ^3^P_1_— 9*s* ${\;}^{5}S_{2}^{{}^{\circ}}$
6764.17	150*c*	14779.70	5*d*′ ${\;}^{3}F_{4}^{{}^{\circ}}$— 6*p*′ ^1^F_3_
6768.30	4*c*	14770.68	6*d* ${\;}^{5}D_{1}^{{}^{\circ}}$— 4*f*′ ^3^D_2_
6769.53	3*c*	14768.00	6*s*″ ${\;}^{3}P_{2}^{{}^{\circ}}$— 4*f* ^3^F_3_
6771.69	12*c*	14763.29	6*s*″ ${\;}^{3}P_{2}^{{}^{\circ}}$— 6*p*″ ^3^D_2_
6776.53	2	14752.74	
6777.46	3	14750.72	
6779.17	8*c*	14747.00	
6787.19	2	14729.57	
6796.21	4	14710.02	5*f* ^5^F_5_—10*g* ${\;}^{5}G_{6}^{{}^{\circ}}$
6801.80	2	14697.93	6*d* ${\;}^{3}D_{1}^{{}^{\circ}}$— 4*f*′ ^3^D_1_
6812.57	4000*Z*	14674.70	5*d* ${\;}^{5}D_{3}^{{}^{\circ}}$— 6*p* ^5^P_3_
6816.46	4*c*	14666.32	7*d* ${\;}^{3}D_{3}^{{}^{\circ}}$— 11_3,4_
6820.93	3	14656.71	
6822.98	2	14652.31	6*d*′ ${\;}^{3}D_{3}^{{}^{\circ}}$— 9*f* ^3^F_4_
6824.09	2	14649.93	5d″ ${\;}^{3}D_{1}^{{}^{\circ}}$— 5*f* ^3^F_2_
6825.23	150*c*	14647.48	6*s*′ ${\;}^{3}D_{3}^{{}^{\circ}}$— 6*p*′ ^3^F_2_
6825.79	3	14646.28	
6826.83	2	14644.05	
6831.15	3	14634.79	7*p* ^3^P_1_— 8*d* ${\;}^{5}D_{2}^{{}^{\circ}}$
6832.89	10	14631.06	
6838.60	3	14618.84	
6855.90	3	14581.95	5*d*″ ${\;}^{3}F_{2}^{{}^{\circ}}$— 7*p*′ ^3^D_3_
6860.47	1*c*	14572.24	6*d* ${\;}^{3}D_{1}^{{}^{\circ}}$— 7*p*′ ^3^F_2_
6869.78	1	14552.49	
6879.12	10	14532.73	6*p*′ ^3^P_1_— 7*s*′ ${\;}^{3}D_{2}^{{}^{\circ}}$
6880.25	3	14530.35	6*p*′ ^3^P_1_— 7*s*′ ${\;}^{3}D_{1}^{{}^{\circ}}$
6888.84	4	14512.23	
6902.13	200*Z*	14484.29	6*s* ${\;}^{3}S_{1}^{{}^{\circ}}$**—** 6*p* ^5^P_2_
6904.77	10*c*	14478.75	6*s*′ ${\;}^{1}D_{2}^{{}^{\circ}}$— 6*p*′ ^3^D_2_
6906.81	12	14474.47	7*p* ^5^P_3_— 9*s* ${\;}^{5}S_{2}^{{}^{\circ}}$
6909.98	2	14467.83	6*p*′ ^1^D_2_— 7*S*′ ${\;}^{3}D_{3}^{{}^{\circ}}$
6956.46	20	14371.16	6*d* ${\;}^{5}D_{4}^{{}^{\circ}}$— 8*p* ^5^P_3_?
6958.78	1000*Z*	14366.37	5*d* ${\;}^{5}D_{4}^{{}^{\circ}}$— 6*p* ^5^P_3_
6970.13	1	14342.98	6*d* ${\;}^{5}D_{4}^{{}^{\circ}}$— 4*f*′ ^3^D_3_
6999.29	1	14283.22	6*d* ${\;}^{5}D_{1}^{{}^{\circ}}$— 4*f*′ ^3^F_2_
7018.91	70*c*	14243.30	5*d*′ ${\;}^{3}D_{3}^{{}^{\circ}}$— 6*p*′ ^3^F_3_
7021.60	20	14237.84	5*d* ${\;}^{5}D_{2}^{{}^{\circ}}$— 6*p* ^5^P_3_
7022.54	2*h*	14235.94	5*d*″ ${\;}^{3}F_{2}^{{}^{\circ}}$— 4*f*′ ^3^D_2_
7027.61	5	14225.67	6*d* ${\;}^{5}D_{2}^{{}^{\circ}}$— 8*p* ^5^P_2_?
7027.88	3	14225.12	5*d*′ ${\;}^{3}S_{1}^{{}^{\circ}}$**—** 6*p*″ ^3^D_1_
7028.04	1	14224.80	
7032.99	300*c*	14214.78	5*d*′ ${\;}^{3}D_{2}^{{}^{\circ}}$— 6*p*′ ^3^F_2_
7042.26	100*c*	14196.07	5*d*′ ${\;}^{3}G_{4}^{{}^{\circ}}$— 6*p*′ ^3^F_4_
7057.21	2*h*	14166.00	5*d*″ ${\;}^{3}D_{1}^{{}^{\circ}}$— 5*f* ^5^F_1_
7057.81	3*h*	14164.80	6*d* ${\;}^{5}D_{1}^{{}^{\circ}}$— 8*p* ^3^P_1_
7059.07	1*h*	14162.27	
7078.72	20	14122.95	5*d*′ ${\;}^{1}S_{0}^{{}^{\circ}}$**—** 6*p*″ ^3^P_1_
7085.21	200*c*	14110.02	5*d*′ ${\;}^{3}G_{4}^{{}^{\circ}}$— 6*p*′ ^3^D_3_
7100.06	15	14080.51	5*d*′ ${\;}^{3}P_{2}^{{}^{\circ}}$— 6*p*″ ^3^S_1_
7101.72	2	14077.22	6*p*′ ^1^P_1_— 5*d*″ ${\;}^{1}P_{1}^{{}^{\circ}}$?
7115.08	10*c*	14050.78	5*p*^5^ ${\;}^{1}P_{1}^{{}^{\circ}}$— 6*p*′ ^3^D_1_
7133.66	2	14014.19	7*d* ${\;}^{5}D_{4}^{{}^{\circ}}$— 9*f* ^5^F_5_?
7138.97	100*c*	14003.76	5*d*′ ${\;}^{3}D_{3}^{{}^{\circ}}$— 6*p*′ ^3^D_2_
7161.45	2	13959.81	
7186.15	20	13911.82	5*d*′ ${\;}^{1}S_{0}^{{}^{\circ}}$— 7*p* ^5^P_1_
7221.85	10*c*	13843.05	
7225.81	100*Z*	13835.47	5*d* ${\;}^{5}D_{0}^{{}^{\circ}}$— 6*p* ^5^P_1_
7248.06	1*c*	13792.99	5*d*″ ${\;}^{1}F_{3}^{{}^{\circ}}$— 7*p*′ ^3^P_2_
7254.78	3*h*	13780.22	5*d*″ ${\;}^{1}F_{3}^{{}^{\circ}}$— 7*p*′ ^3^D_3_
7282.83	40c, *Z*	13727.14	5*p*^4 3^P_2_— 5*p*^4 1^D_2_
7289.47	1	13714.64	5*f* ^5^F_3_— 9*g* ${\;}^{5}G_{4}^{{}^{\circ}}$?
7291.34	1	13711.12	5*f* ^5^F_4_—9*g* ${\;}^{5}G_{5}^{{}^{\circ}}$
7311.57	2	13673.19	5*f* ^5^F_5_— 9*g* ${\;}^{5}G_{6}^{{}^{\circ}}$
7333.13	1	13632.99	5*f* ^5^F_2_— 9*g* ${\;}^{5}G_{3}^{{}^{\circ}}$
7342.10	1	13616.33	5*f* ^5^F_1_— 9*g* ${\;}^{5}G_{2}^{{}^{\circ}}$
7351.35	500*Z*	13599.20	5*d* ${\;}^{5}D_{3}^{{}^{\circ}}$— 6*p* ^5^P_2_
7370.31	3	13564.21	6*d* ${\;}^{5}D_{3}^{{}^{\circ}}$— 5*f* ^3^F_3_
7370.83	4*c*	13563.26	6*s*″ ${\;}^{1}P_{1}^{{}^{\circ}}$— 4*f* ^3^F_2_
7383.49	15	13540.00	6*d* ${\;}^{5}D_{0}^{{}^{\circ}}$— 5*f* ^5^F_1_
7392.26	10	13523.94	6*d* ${\;}^{5}D_{2}^{{}^{\circ}}$— 5*f* ^3^F_3_
7395.57	5	13517.89	6*d*′ $8_{4}^{{}^{\circ}}$— 11_3,4_
7398.91	4*h*	13511.78	$\{\;\begin{array}{cccc} 5f & {\;}^{3}F_{3}- & 9g & {\;}^{3}G_{4}^{{}^{\circ}}\\ 5f & {\;}^{3}F_{3}- & 9g & {\;}^{5}G_{3}^{{}^{\circ}} \end{array}$
7400.29	20*c*	13509.26	5*d*″ ${\;}^{3}P_{2}^{{}^{\circ}}$— 6*p*″ ^1^D_2_
7407.75	5*c*	13495.66	
7418.52	6	13476.07	4*f*′ ^3^F_2_— $42_{2}^{{}^{\circ}}$
7423.85	15	13466.39	
7435.26	2*c*	13445.73	
7437.64	2	13441.42	
7437.87	4	13441.01	
7438.90	12	13439.15	
7444.29	25	13429.42	6*p*″ ^1^S_0_**—** $32_{1}^{{}^{\circ}}$
7454.79	3	13410.50	
7454.94	5	13410.23	
7459.00	20	13402.93	6*d* ${\;}^{5}D_{2}^{{}^{\circ}}$— 5*f* ^5^F_2_
7461.52	2*d*	13398.41	7*p* ^3^P_1_— 6*d*′ $14_{3}^{{}^{\circ}}$
7477.07	5*c*	13370.54	5*d*′ ${\;}^{3}P_{1}^{{}^{\circ}}$— 6*p*″ ^1^D_2_
7481.52	25	13362.59	6*d* ${\;}^{5}D_{3}^{{}^{\circ}}$— 5*f* ^5^F_4_
7482.88	10	13360.16	6*d* ${\;}^{5}D_{3}^{{}^{\circ}}$— 5*f* ^5^F_3_
7484.77	70	13356.79	6*d* ${\;}^{5}D_{4}^{{}^{\circ}}$— 5*f* ^5^F_5_
7488.87	5	13349.47	
7505.54	30	13319.82	6*d* ${\;}^{5}D_{2}^{{}^{\circ}}$— 5*f* ^5^F_3_
7506.31	6*h*	13318.46	$\{\;\begin{array}{cccc} 5d^{\prime \prime } & {\;}^{3}F_{2}^{{}^{\circ}}- & 4f^{\prime } & {\;}^{3}F_{3}\\ 7p & {\;}^{3}P_{0}- & 6d^{\prime } & 15_{1}^{{}^{\circ}} \end{array}$
7508.34	10	13314.86	6*d* ${\;}^{5}D_{4}^{{}^{\circ}}$— 5*f* ^5^F_4_
7535.77	5	13266.39	
7544.05	3	13251.83	
7557.96	25	13227.44	
7563.20	25	13218.28	
7563.66	2*c*	13217.47	
7569.15	25	13207.89	
7573.40	7*d*	13200.48	6*s*″ ${\;}^{1}P_{1}^{{}^{\circ}}$— 6*p*″ ^3^P_1_
7578.96	20	13190.79	
7579.95	4	13189.07	
7585.53	2*c*	13179.37	
7592.03	1	13168.08	5*f* ^3^F_4_— 9*g* ${\;}^{5}G_{5}^{{}^{\circ}}$
7595.37	300*Z*	13162.29	5*d* ${\;}^{5}D_{2}^{{}^{\circ}}$— 6*p* ^5^P_2_
7596.33	20	13160.63	6*d*′ $1_{2}^{{}^{\circ}}$— 1_2,3_
7612.27	1	13133.07	5*f* ^3^F_2_— 9*g* ${\;}^{3}G_{3}^{{}^{\circ}}$?
7617.95	3*h*	13123.28	6*d*′ $3_{3}^{{}^{\circ}}$— 8*f* ^3^F_4_
7618.50	100*c*	13122.33	5*d*′ ${\;}^{3}D_{3}^{{}^{\circ}}$— 6*p*′ ^3^F_2_
7623.23	20	13114.19	
7627.31	4*h*	13107.17	
7636.62	2	13091.20	7*d* ${\;}^{3}D_{3}^{{}^{\circ}}$— 9*f* ^3^F_4_
7640.28	7*c*	13084.92	
7654.26	1*c*	13061.03	7*d* ${\;}^{3}D_{3}^{{}^{\circ}}$— 4_3,4_
7657.94	150*c*, *Z*	13054.75	5*d* ${\;}^{5}D_{2}^{{}^{\circ}}$— 6*p* ^5^P_1_
7665.69	200*c*	13041.55	5*d*′ ${\;}^{3}F_{4}^{{}^{\circ}}$— 6*p*′ ^3^F_3_
7683.47	8*c*	13011.37	5*d*′ ${\;}^{1}F_{3}^{{}^{\circ}}$— 7*p* ^3^P_2_
7684.82	1*c*	13009.09	
7685.30	3*c*	13008.27	
7690.55	40*c*	12999.39	5*d*′ ${\;}^{3}G_{4}^{{}^{\circ}}$— 6*p*′ ^1^F_3_
7691.29	3	12998.14	6*d* ${\;}^{5}D_{1}^{{}^{\circ}}$— 5*f* ^5^F_1_
7703.21	5	12978.03	6*d* ${\;}^{5}D_{1}^{{}^{\circ}}$— 5*f* ^5^F_2_
7707.95	3*c*	12970.05	
7713.17	4*h*	12961.27	
7724.21	3	12942.75	
7735.78	75*c*	12923.39	5*d*′ ${\;}^{3}D_{2}^{{}^{\circ}}$— 6*p*′ ^3^D_1_
7756.05	15	12889.61	6*d*″ ${\;}^{2}P_{2}^{{}^{\circ}}$*—* 6*p*″ ^1^P_1_
7757.58	5	12887.07	
7769.09	1*c*	12867.98	5*d*″ ${\;}^{1}D_{2}^{{}^{\circ}}$— 7*p* ^3^P_1_
7786.18	2	12839.74	
7787.04	3*c*	12838.32	6*p* ^3^P_1_— 5*d*′ ${\;}^{3}P_{2}^{{}^{\circ}}$
7798.98	500	12818.66	
7824.91	3	12776.18	
7840.37	2	12750.99	} 5*d*′ ${\;}^{3}P_{1}^{{}^{\circ}}$— 6*p*″ ^1^P_1_
7840.46	2	12750.85	
7843.31	3*c*	12746.21	6*d*′ $9_{3}^{{}^{\circ}}$— 11_3,4_
7862.79	3	12714.63	6*d*′ $9_{3}^{{}^{\circ}}$— 10_2,3_
7876.51	1	12692.49	
7881.21	1	12684.92	7*d* ${\;}^{5}D_{2}^{{}^{\circ}}$— 8*f* ^3^F_3_
7884.39	10*c*	12679.80	
7886.55	2*c*	12676.33	
7886.82	2	12675.89	
7887.70	3	12674.48	
7905.57	1	12645.83	
7925.58	2*c*	12613.90	
7956.47	1	12564.93	7*d* ${\;}^{5}D_{2}^{{}^{\circ}}$— 8*f* ^5^F_3_
7956.89	3*c*	12564.27	5*d*″ ${\;}^{3}F_{2}^{{}^{\circ}}$— 5*f* ^3^F_3_
7968.61	5	12545.79	
7990.27	2	12511.78	
7991.36	10*c*	12510.07	5*d*″ ${\;}^{1}F_{3}^{{}^{\circ}}$— 4*f*′ ^3^G_4_
7993.29	3	12507.05	
7994.65	2*c*	12504.93	6*d*′ $2_{3}^{{}^{\circ}}$— 1_2,3_
8000.09	2	12496.42	7*d* ${\;}^{5}D_{3}^{{}^{\circ}}$— 8*f* ^5^F_4_
8008.32	2	12483.58	
8009.28	3	12482.08	
8009.84	1	12481.21	
8014.03	1	12474.69	
8049.51	3	12419.70	7*d* ${\;}^{5}D_{4}^{{}^{\circ}}$— 8*f* ^5^F_5_
8055.06	20	12411.14	
8056.54	2	12408.86	
8060.37	1	12402.97	7*d* ${\;}^{5}D_{1}^{{}^{\circ}}$— 8*f* ^5^F_2_
8150.57	1	12265.71	5*f* ^5^F_4_—8*g* ${\;}^{3}G_{5}^{{}^{\circ}}$
8152.46	2	12262.87	5*f* ^5^F_3_—8*g* ${\;}^{5}G_{4}^{{}^{\circ}}$
8153.83	30*c*	12260.80	6*p*″ ^3^P_2_— 6*g* ${\;}^{5}G_{2}^{{}^{\circ}}$
8154.53	3	12259.75	5*f* ^5^F_4_— 8*g* ${\;}^{5}G_{5}^{{}^{\circ}}$
8159.49	10	12252.30	5*d*′ ${\;}^{1}G_{4}^{{}^{\circ}}$— 6*p*′ ^3^F_4_
8162.42	1	12247.90	8*p* ^3^P_1_— $37_{2}^{{}^{\circ}}$
8166.29	1	12242.10	6*p*″ ^1^P_1_— 7s″ ${\;}^{3}P_{0}^{{}^{\circ}}$
8170.07	100*c*	12236.43	5*d*′ ${\;}^{3}F_{3}^{{}^{\circ}}$— 6*p*′ ^3^D_2_
8177.60	1*c*	12225.17	
8179.15	4	12222.85	5*f* ^5^F_5_— 8*g* ${\;}^{5}G_{6}^{{}^{\circ}}$
8180.76	1*c*	12220.44	
8183.27	30	12216.70	
8185.11	8*c*	12213.95	
8194.84	40	12199.45	
8205.68	2	12183.33	
8206.08	4	12182.74	
8206.49	2	12182.13	5*f* ^5^F_2_— 8*g* ${\;}^{5}G_{3}^{{}^{\circ}}$
8217.11^e^	8	12166.38	5*f* ^5^F_1_— 8*g* ${\;}^{5}G_{2}^{{}^{\circ}}$
8217.27	30	12166.15	5*d*′ ${\;}^{1}G_{4}^{{}^{\circ}}$— 6*p*′ ^3^D_3_
8235.20	15	12139.66	
8246.21	30	12123.45	5*d*″ ${\;}^{3}D_{2}^{{}^{\circ}}$— 6*p*″ ^1^D_2_
8251.79	100*c*, *Z*	12115.25	5*d* ${\;}^{3}D_{2}^{{}^{\circ}}$— 6*p* ^3^P_2_
8253.84	25	12112.24	5*d*′ ${\;}^{3}P_{0}^{{}^{\circ}}$— 6*p*′ ^3^D_1_
8256.54	1*c*	12108.28	7*p*′ ^3^P_2_— $40_{3}^{{}^{\circ}}$
8272.90	1*c*	12084.34	5*d*′ ${\;}^{1}F_{3}^{{}^{\circ}}$— 4*f* ^3^F_4_
8286.65	2*c*	12064.29	
8288.55	2	12061.52	5*f* ^3^F_3_— 8*g* ${\;}^{3}G_{4}^{{}^{\circ}}$
8296.73	2*c*	12049.63	4*f* ^5^F_4_— 6*d*′ $8_{4}^{{}^{\circ}}$
8307.51	2	12033.99	6*d* ${\;}^{3}D_{1}^{{}^{\circ}}$— 7*p*′ ^3^P_2_
8316.16	15*c*	12021.48	$\{\;\begin{array}{cccc} 4f & {\;}^{5}F_{3}- & 6d^{\prime } & 8_{4}^{{}^{\circ}}\\ 7p & {\;}^{5}P_{3}- & 6d^{\prime } & 9_{3}^{{}^{\circ}} \end{array}$
8321.03	5*c*	12014.44	
8323.23	4	12011.27	
8330.75	1*h*	12000.42	8*p* ^3^P_2_— 11*d* ${\;}^{3}D_{3}^{{}^{\circ}}$
8356.90	2*c*	11962.87	6*d*′ $10_{2}^{{}^{\circ}}$— 12_1,2_
8366.51	3*c*	11949.13	
8372.94	35	11939.96	4*f* ^5^F_4_— 5*g* ${\;}^{3}G_{5}^{{}^{\circ}}$
8380.68	1*c*	11928.93	6*p*″ ^1^S_0_— $21_{1}^{{}^{\circ}}$
8385.74	6*c*	11921.73	$\{\;\begin{array}{cccc} 5d^{\prime } & {\;}^{3}P_{2}^{{}^{\circ}}- & 7p & {\;}^{3}P_{2}\\ 4f & {\;}^{5}F_{3}- & 5g & {\;}^{3}G_{3}^{{}^{\circ}} \end{array}$
8398.74	3*h*	11903.28	6*d*′ $10_{2}^{{}^{\circ}}$— 10_2,3_
8400.03	15	11901.45	4*f* ^5^F_3_— 5*g* ${\;}^{5}G_{3}^{{}^{\circ}}$
8403.62	12*c*	11896.36	4*f* ^5^F_4_— 5*g* ${\;}^{5}G_{4}^{{}^{\circ}}$
8408.67	80	11889.22	4*f* ^5^F_4_— 5*g* ${\;}^{5}G_{5}^{{}^{\circ}}$
8412.78	6	11883.41	4*f* ^5^F_3_— 5*g* ${\;}^{3}G_{4}^{{}^{\circ}}$
8414.32^f^		11881.24	4*f* ^5^F_5_— 5*g* ${\;}^{3}G_{5}^{{}^{\circ}}$
8414.60	150*Z*	11880.84	4*f* ^5^F_5_— 5*g* ${\;}^{5}G_{6}^{{}^{\circ}}$
8417.50	2	11876.75	
8418.93	7	11874.73	
8421.01	7	11871.80	
8423.60	60*w*, *Z*	11868.15	4*f* ^5^F_3_— 5*g* ${\;}^{5}G_{4}^{{}^{\circ}}$
8436.08	20	11850.59	4*f* ^5^F_2_— 5*g* ${\;}^{3}G_{3}^{{}^{\circ}}$
8436.92	5	11849.41	4*f* ^5^F_2_— 5*g* ${\;}^{5}G_{2}^{{}^{\circ}}$
8445.31	6*c*	11837.64	$\{\;\begin{array}{cccc} 5d^{\prime \prime } & {\;}^{3}P_{2}^{{}^{\circ}}- & 6p^{\prime \prime } & {\;}^{3}P_{2}\\ 4f & {\;}^{5}F_{5}- & 5g & {\;}^{5}G_{4}^{{}^{\circ}} \end{array}$
8450.62	30*Z*	11830.20	4*f* ^5^F_2_— 5*g* ${\;}^{5}G_{3}^{{}^{\circ}}$
8455.66	8	11823.15	
8474.57	2*h*	11796.77	
8475.37	40	11795.65	
8476.46	10*c*	11794.14	5*d*″ ^1^D_2_— 6*p*″ ^3^P_1_
8478.21	1*h*	11791.70	
8478.85	50	11790.81	4*f* ^5^F_1_—5*g* ${\;}^{5}G_{2}^{{}^{\circ}}$
8479.80	30	11789.49	
8497.89	2	11764.40	
8505.90	20	11753.32	5*d*′ ${\;}^{3}P_{2}^{{}^{\circ}}$— 7*p* ^3^P_1_
8528.14	1	11722.67	5*f* ^3^F_4_— 8*g* ${\;}^{3}G_{5}^{{}^{\circ}}$
8556.54	1	11683.76	5*f* ^3^F_2_— 8*g* ${\;}^{3}G_{3}^{{}^{\circ}}$
8564.35	3	11673.10	
8605.13	10	11617.78	
8606.54	2	11615.88	
8623.44	1*Z*	11593.12	5*d* ${\;}^{5}D_{1}^{{}^{\circ}}$— 6*p* ^5^P_2_
8638.89	3	11572.38	
8639.88	3	11571.06	5*d*′ ${\;}^{3}F_{2}^{{}^{\circ}}$— 6*p*′ ^1^D_2_
8643.72	3	11565.92	5*d*″ ${\;}^{3}D_{3}^{{}^{\circ}}$— 6*p*″ ^3^D_2_
8704.22	10*Z*	11485.53	5*d* ${\;}^{5}D_{1}^{{}^{\circ}}$— 6*p* ^5^P_1_
8793.87	10*Z*	11368.44	4*f* ^3^F_3_— 5*g* ${\;}^{3}G_{4}^{{}^{\circ}}$
8804.23	10*c*	11355.06	5*d*′ ${\;}^{3}F_{3}^{{}^{\circ}}$— 6*p*′ ^3^F_2_
8877.61	20	11261.20	5*d*′ ${\;}^{3}G_{4}^{{}^{\circ}}$— 6*p*′ ^3^F_3_
8968.84^g^		11146.66	4*f* ^3^F_4_— 5*g* ${\;}^{3}G_{5}^{{}^{\circ}}$
8969.42	30*Z*	11145.93	5*d* ${\;}^{3}D_{2}^{{}^{\circ}}$— 6*p* ^3^P_1_
8999.07	7	11109.21	4*f* ^3^F_2_— 5*g* ${\;}^{3}G_{3}^{{}^{\circ}}$
9009.86	10	11095.91	4*f* ^3^F_4_— 5*g* ${\;}^{5}G_{5}^{{}^{\circ}}$
9015.63	2	11088.80	4*f* ^3^F_2_— 5*g* ${\;}^{5}G_{3}^{{}^{\circ}}$
9036.92	2	11062.68	
9042.76	40	11055.54	5*d*′ ${\;}^{1}G_{4}^{{}^{\circ}}$— 6*p*′ ^1^F_3_
9063.59	1	11030.13	
9185.41	3	10883.84	6s″ ${\;}^{3}P_{0}^{{}^{\circ}}$— 6*p*′ ^3^P_1_
9195.87	30	10871.46	5*d* ${\;}^{3}D_{1}^{{}^{\circ}}$— 6*p* ^3^P_0_
9214.67	2	10849.28	6*d* ${\;}^{3}D_{2}^{{}^{\circ}}$— 4*f*′ ^3^F_3_
9236.12	6	10824.09	
9244.02	1	10814.84	
9255.17	3	10801.81	6*d* ${\;}^{3}D_{3}^{{}^{\circ}}$— 4*f*′ ^3^G_4_
9259.42	1	10796.85	6*d* ${\;}^{3}D_{2}^{{}^{\circ}}$— 8*p* ^5^P_2_?
9260.84	2	10795.19	4*f*′ ^3^G_3_— $47_{1,2}^{{}^{\circ}}$
9262.73	3	10792.99	
9265.97	4	10789.22	
9274.68	1*h*	10779.09	
9302.41	1	10746.95	
9304.65	2*c*	10744.37	5*d*′ ${\;}^{3}P_{2}^{{}^{\circ}}$— 4*f* ^3^F_3_
9361.26	1	10679.39	5*d*′ ${\;}^{3}P_{2}^{{}^{\circ}}$— 6*p*″ ^3^P_1_
9394.32	1*h*	10641.81	
9405.73	2	10628.90	
9406.75	1	10627.75	5*d*″ ${\;}^{3}P_{2}^{{}^{\circ}}$— 6*p*″ ^3^S_1_
9420.09	1	10612.70	
9480.33	10*c*	10545.26	5*d*′ ${\;}^{1}D_{2}^{{}^{\circ}}$— 6*p*′ ^1^D_2_
9506.70	1	10516.01	
9538.51	3	10480.94	5*d* ${\;}^{3}D_{1}^{{}^{\circ}}$— 6*p* ^3^P_2_
9541.02	1	10478.19	6*d* ${\;}^{3}D_{2}^{{}^{\circ}}$— 5*f* ^3^F_2_
9616.67	4	10395.76	6*d* ${\;}^{3}D_{3}^{{}^{\circ}}$— 5*f* ^3^F_4_
9625.06	4	10386.70	6*d* ${\;}^{3}D_{1}^{{}^{\circ}}$— 5*f* ^3^F_2_
9766.73	1	10236.03	
9908.38	1	10089.70	5*d*′ ${\;}^{3}D_{1}^{{}^{\circ}}$— 6*p*′ ^3^P_0_
10405.49	6	9607.68	5*d* ${\;}^{3}D_{3}^{{}^{\circ}}$— 6*p* ^3^P_2_
10418.15	1	9596.00	7*p* ^5^P_1_— 7*d* ${\;}^{5}D_{1}^{{}^{\circ}}$
10691.12	1	9351.00	
11084.68	1	9018.99	7*p* ^5^P_3_— 7*d* ${\;}^{5}D_{4}^{{}^{\circ}}$

Meanings of the letters following the intensities in this table:

*c* Complex line, showing resolved or partially resolved structure. Most of the lines having large intensities not followed by this symbol actually have complex structures masked by line width.

*d* Double line. In the air region this is usually hyperfine structure.

*h* Ha*Z*y line.

*w* Wide line.

*Z Z*eeman pattern given in table 10.2.

Notes:

a The wavelength of this line is calculated, since it is partly masked by the II line at 4850.35 A.

b The line at this position belongs partly to II.

c The relative intensities of this line in the high and low pressure discharges indicate that it belongs primarily to the II spectrum; however, since both 7*s*″ ^3^P_0_ and 7*s*″ ^3^P_1_ of I ii combine with 7*p* levels, it is likely that the transition given here occurs.

d Part of this double line is due to an II transition.

e The intensity of this line is given as it appears on the plate. The line at 8217.27 A probably has a component overlapping the weaker line expected at 8217.11 A.

f The calculated wavelength of this line is coincident with an II line of intensity 40.

g Line not resolved from the II line of intensity 300 at 8969.04 A. The wavelength given for the expected I II line is a calculated value.

**Table 10.2:** Zeeman effect data for *I ii*

λ	Zeeman patterns
4173.810	(…) 0.659, 1.434
4225.542	(0.00*w*) 1.187*w*
4235.511	(…) **0.719**, 1.095
4342.078	(0.841) 1.467, 2.304
4376.139	(0.00*w*) 0.795 *A*
4403.566	(0.00*w*) 1.238 *B*
4408.954	(0.00*w*) 0.841 *A*
4412.333	(…) 1.747
4423.725	(**0.000**, 0.873) **0.602**, 1.440, 2.301
4428.195	(0.00*w*) 1.33*w*
4442.562	(0.000) 0.687
4444.873	(0.272, **0.550**) 1.187, **1.452, 1.715**, 1.980
4446.742	(0.000) 2.308
4452.858	(**0.000**, 0.264, 0.548) **0.862**, 1.108, 1.432, 1.718
4456.625	(…) 1.388, **1.821**
4460.185	(1.512) 0.000
4464.338	(0.00*w*) 1.127*w*
4473.412	(0.00*w*) 1.252 *B*
4476.037	(0.000) 0.674
4488.552	(0.586) 0.908, 1.497
4540.644	(…) **1.084**, 1.425
4560.612	(0.00*w*) 1.136*w*
4574.090	(0.841) 1.438, 2.298
4576.550	(0.00*w*) 0.465*w*
4590.930	(0.00*w*) 1.203 *B*
4599.769	(0.00*w*) 0.561 *A*
4611.224	(0.330) **0.671**, 1.083
4621.854	(0.00*w*) 1.084*w*
4632.446	(0.423, **0.814**) 1.160, **1.514, 1.899**, 2.314
4675.530	(0.168*w*) 1.048*w*
4806.402	(0.453*w*) 0.794, **1.105, 1.310***w*
4828.252	(…) 1.478*w*
4884.828	(0.00*w*) 1.104*w*
4986.922	(**0.000**, 0.484) 0.686, 1.157, **1.629**
5046.43	(0.000) 0.656
5065.37	(0.00*w*) 1.565*w*
5156.41	(**0.000**, 0.406) **1.507**, 1.891, 2.319
5161.20	(**0.000**, 0.267, 0.589) **1.052**, 1.343, 1.720, 2.045
5214.08	(0.00*w*) 1.590 *B*
5216.27	(**0.000**, 0.218) 0.924, **1.143**
5245.71	(0.00*w*) 1.353*w*
5265.16	(0.351) 0.643, 1.016
5322.80	(0.000) 1.536
5338.22	(0.00*w*) 1.025*w*
5345.15	(0.00*w*) 1.139*w*
5405.42	(0.00*w*) 1.622*w*
5438.00	(**0.000**, 0.380) **0.629**, 1.013, 1.397
5491.50	(**0.000**, 0.262, 0.532) **1.084**, 1.353, 1.623, 1.882
5504.72	(0.000) 1.773
5593.12	(0.224) 0.680, 0.903
5625.69	(**0.000**, 0.253) 1.232, 1.494, 1.743
5678.08	(0.329, **0.659**) 0.567, 0.892, 1.242, 1.584
5690.91	(**0.000**, 0.208, 0.415) 1.196, 1.454, **1.631**
5702.05	(0.00*w*) 1.064*w*
5710.53	(0.362, **0.529**) 1.275*w*
5738.27	(0.000) 1.519
5760.72	(**0.000**, 0.524) 0.649, 1.170, 1.681
5920.59	(0.00*w*) 1.402*w*
5950.25	(0.240) 1.514, 1.760
6068.93	(**0.000**, 0.243) 0.911, **1.145**
6074.98	(**0.000**, 0.139, 0.286) … 1.269, **1.393**
6127.49	(**0.000**, 0.309) 0.932, 1.219, **1.548**
6148.34	(0.000) 1.533
6204.86	(0.00*w*) 1.026*w*
6516.18	(0.00*w*) 0.847*w*
6665.96	(0.785) 1.525, 2.316
6718.83	(0.00*w*) 1.518*w*
6812.57	(0.168, 0.341, **0.496**) 1.131, 1,303, **1.462, 1.623**, 1.791, 1.949
6902.13	(0.00*w*) 1.715*w*
6958.78	(**0.000**, 0.151, 0.300, 0.446) **1.028**, 1.173, 1.330, 1.491
7225.81	(0.000) 2.310
7282.83	(… 1.042*w*, 1.459, 1.860) 0.393, **0.808***W*
7351.35	(**0.000**, 0.257, 0.527) **0.943**, 1.199, 1.472, 1.730, 1.968
7595.37	(0.309, **0.570**) 1.136, **1.435, 1.737**, 2.047
7657.94	(**0.000**, 0.874) **0.561**, 1.436, 2.316
8251.79	(0.372, **0.759**) 0.751, **1.123, 1.502**, 1.886
8414.60	(0.00*W*) 1.05*w*
8423.60	(0.00*W*) 1.03*w*
8450.62	(0.00*W*) 0.69*w*
8623.44	(**0.000**, 0.304) 1.728, **1.995**
8704.22	(0.823) **1.483**, 2.325
8793.87	(0.00*w*) 0.97*w*
8969.42	(**0.000**, 0.440) **0.713**, 1.134

The magnetic displacements, in Lorentz units, of the Zeeman components with polarization parallel to the field are given in parentheses. The displacements of the perpendicularly-polarized components follow, outside the parentheses. Bold type indicates strongest components.

The letters *A* and *B* in this table indicate the type of shading displayed by the unresolved patterns:

*A* indicates ,

*B* indicates ,

*w* Wide line, usually several unresolved components,

*W* Very wide line.

Unresolved or incomplete patterns were treated as “blend” or “strongest line only” patterns in the calculation of *g_J_* values.

**Table 10.3:** Even levels of *I ii*

Designation	Level	*g_j_*
		
5*p*^4 3^P_2_	0.0	1.457
5*p*^4^ ^3^P_1_	7087.0	1.51
5*p*^4 3^P_0_	6447.9	
5*p*^4 1^D_2_	13727.2	1.046
5*p*^4 1^S_0_	29501.3	
6*p ^5^*P_1_	99219.61	2.309
6*p* ^5^P_2_	99327.14	1.714
6*p* ^5^P_3_	100402.68	1.638
6*p* ^3^P_2_	102613.52	1.501
6*p* ^3^P_1_	101644.21	1.520
6*p* ^3^P_0_	103004.04	
6*p*′ ^3^D_1_	110006.71	0.915
6*p*′ ^3^F_2_	111298.09	0.915
6*p*′ ^3^D_2_	112179.49	1.158
6*p*′ ^3^F_3_	112419.04	1.12
6*p*′ ^1^P_1_	113812.79	0.985
6*p*′ ^1^F_3_	114157.21	1.13
6*p*′ ^3^P_0_	114635.82	
6*p*′ ^3^D_3_	115267.82	1.212
6*p*′ ^3^F_4_	115353.94	1.29
6*p*′ ^3^P_2_	115708.45	1.425
6*p*′ ^3^P_1_	116084.84	1.39
6*p*′ ^1^D_2_	119082.78	1.11
6*p″* ^3^D_1_	123520.74	
6*p*″^3^P_0_	125006.38	
6*p*″ ^3^P_1_	125162.00	
6*p*″ ^3^D_2_	125222.20	0.83 L^a^
6*p*″ ^3^S_1_	128563.14	1.73 L
6*p*″ ^3^D_3_	129431.27	1.32 L
6*p*″ ^3^P_2_	129772.76	
6*p*″ ^1^P_1_	130824.99	
6*p*″ ^1^D_2_	131444.69	1.26 L
6*p*″ ^1^S_0_	134363.21	
4*f* ^5^F_5_	124742.50	
4*f* ^5^F_4_	124683.79	
4*f* ^5^F_3_	124711.93	
4*f* ^5^F_2_	124783.18	
4*f* ^5^F_1_	124841.77	
7*p* ^5^P_1_	124950.92	
7*p* ^5^P_2_	125084.32	
7*p* ^5^P_3_	125483.51	
4*f* ^3^F_4_	125477.08	
4*f* ^3^F_3_	125226.92	
4*f* ^3^F_2_	125524.56	0.83 L
7*p* ^3^P_2_	126404.16	
7*p* ^3^P_1_	126235.91	
7*p* ^3^P_0_	127181.90	
5*f* ^5^F_5_	135182.87	
5*f* ^5^F_4_	135140.95	
5*f* ^5^F_3_	135138.51	
5*f* ^5^F_2_	135221.58	
5*f* ^3^F_1_	135241.72	
5*f* ^3^F_4_	135684.08	
5*f* ^3^F_3_	135342.60	
5*f* ^3^F_2_	135725.82	
8*p* ^5^P_2_?	136044.28	
8*p* ^5^P_3_?	136197.24	
4*f*′ ^3^G_4_	136090.15	
4*f*′ ^3^F_3_	136096.79	
4*f*′ ^3^D_3_	136169.16	
4*f*′ ^3^F_2_	136526.91	
4*f*′ ^3^H_5_	136752.36	
4*f*′ ^3^H_4_	136802.13	
4*f*′ ^3^D_2_	137014.34	
4*f*′ ^3^P_1_	137393.02	
4*f*′ ^3^P_2_	137640.93	
4*f*′ ^3^P_0_	138236.41	
4*f*′ ^3^G_5_	139449.71	
4*f*′^3^F_4_	139499.57	
4*f*′ ^3^G_3_	139691.25	
4*f*′ ^1^G_4_	139719.12	
4*f*′ ^3^D_1_	140036.78	
4*f*′ ^1^F_3_	140387.24	
4*f*′ ^1^D_2_	140492.31	
4*f*′ ^1^P_1_	140560.53	
7*p*′ ^3^D_1_	136209.25	
7*p*′ ^3^P_1_	136659.84	
7*p*′ ^3^D_3_	137360.30	
7*p*′ ^3^P_2_	137373.06	
7*p*′ ^3^F_2_	139911.38	
7*p*′ ^3^F_4_	140108.06	
7*p*′ ^1^F_3_	140163.63	
7*p*′ ^1^D_2_	140189.27	
7*p*′ ^1^P_1_	140254.97	
7*p*′ ^3^P_0_	140327.95	
7*p*′ ^3^F_3_	140383.95	
7*p*′ ^3^D_2_	142039.80	
8*p* ^3^P_2_	136700.57	
8*p* ^3^P_1_	136408.45	
6*f* ^5^F_5_	141088.90	
6*f* ^5^F_4_	141073.17	
6*f* ^5^F_3_	141080.89	
6*f* ^5^F_2_	141094.09 ?	
6*f* ^3^F_2_	141471.00	
6*f* ^3^F_3_	141398.31	
6*f* ^3^F_4_	141529.89	
7*f* ^5^F_5_	144629.91	
7*f* ^5^F_4_	144619.97	
7*f* ^5^F_3_	144630.32	
7*f* ^3^F_2_	144896.23	
7*f* ^3^F_3_	144810.22	
7*f* ^3^F_4_	144919.15	
$\begin{array}{ll} \begin{array}{c} 5f^{\prime }\\ 4f^{\prime \prime }\\ 7p^{\prime \prime }\\ 8p^{\prime } \end{array} & \{\begin{array}{l} 1_{2,3}\\ 2_{2,3}\\ 3_{2,3}\\ 4_{3,4}\\ 5_{2,3}\\ 6_{2,3,4}\\ 7_{2,3}\\ 8_{1,2}\\ 10_{2,3}\\ 11_{3,4}\\ 12_{1,2}\\ 13_{2,3} \end{array} \end{array}$	146460.44	
	146973.00	
	147942.43	
	148646.01	
	148830.03	
	148972.30	
	149204.87	
	149367.05	
	150219.69	
	150251.30	
	150279.40	
	150648.09	
8*f* ^5^F_5_	146922.08	
8*f* ^5^F_4_	146938.07	
8*f* ^5^F_3_	146934.47	
8*f* ^5^F_2_	146949.97	
8*f* ^3^F_3_	147054.27	
8*f* ^3^F_4_	147147.10	
9*f* ^5^F_5_	148516.58 ?	
9*f* ^5^F_4_	148513.52 ?	
9*f* ^5^F_3_	148519.48 ?	
9*f* ^3^F_4_	148676.16	

a The letter “L” following a *g_J_* value indicates that the value is taken from Lacroute [3].

**Table 10.4:** Odd levels of *I ii*

Designation	Level	*g_J_*
		
6*s* ${\;}^{5}S_{2}^{{}^{\circ}}$	81032.70	1.95
6*s* ${\;}^{3}S_{1}^{{}^{\circ}}$	84842.87	1.753
5*p*^5^ ${\;}^{3}P_{2}^{{}^{\circ}}$	81907.83	1.54
5*p*^5^ ${\;}^{3}P_{1}^{{}^{\circ}}$	84222.19	1.530
5*p*^5^ ${\;}^{3}P_{0}^{{}^{\circ}}$	90404.95	
5*d* ${\;}^{5}D_{0}^{{}^{\circ}}$	85384.15	
5*d* ${\;}^{5}D_{1}^{{}^{\circ}}$	87734.06	1.472
5*d* ${\;}^{5}D_{2}^{{}^{\circ}}$	86164.86	1.434
5*d* ${\;}^{5}D_{3}^{{}^{\circ}}$	85727.98	1.457
5*d* ${\;}^{5}D_{4}^{{}^{\circ}}$	86036.32	1.480
5*d* ${\;}^{3}D_{1}^{{}^{\circ}}$	92132.61	0.685
5*d* ${\;}^{3}D_{2}^{{}^{\circ}}$	90498.31	1.138
5*d* ${\;}^{3}D_{3}^{{}^{\circ}}$	93005.95	1.35
6*s*′ ${\;}^{3}D_{1}^{{}^{\circ}}$	94825.33	0.653
6*s*′ ${\;}^{3}D_{2}^{{}^{\circ}}$	93691.35	1.16
6*s*′ ${\;}^{3}D_{3}^{{}^{\circ}}$	96650.55	1.36
5*p*^5^ ${\;}^{1}P_{1}^{{}^{\circ}}$	95955.85	1.05
6*s*′ ${\;}^{1}D_{2}^{{}^{\circ}}$	97700.81	0.996
5*d*′ ${\;}^{3}D_{2}^{{}^{\circ}}$	97083.30	1.26 L^a^
5*d*′ ${\;}^{3}P_{0}^{{}^{\circ}}$	97894.42	
5*d*′ ${\;}^{3}D_{3}^{{}^{\circ}}$	98175.75	
5*d*′ ${\;}^{3}F_{4}^{{}^{\circ}}$	99377.47	
5*d*′ ${\;}^{3}F_{3}^{{}^{\circ}}$	99943.10	
5*d*′ ${\;}^{3}G_{4}^{{}^{\circ}}$	101157.79	
5*d*′ ${\;}^{1}G_{4}^{{}^{\circ}}$	103101.57	
5*d*′ ${\;}^{3}D_{1}^{{}^{\circ}}$	104546.19	
5*d*′ ${\;}^{3}F_{2}^{{}^{\circ}}$	107511.77	
5*d*′ ${\;}^{3}G_{3}^{{}^{\circ}}$	107625.89	
5*d*′ ${\;}^{1}D_{2}^{{}^{\circ}}$	108537.50	1.45 L
5*d*′ ${\;}^{3}S_{1}^{{}^{\circ}}$	109295.45	
5*d*′ ${\;}^{1}S_{0}^{{}^{\circ}}$	111039.06	
5*d*′ ${\;}^{1}F_{3}^{{}^{\circ}}$	113392.94	
5*d*′ ${\;}^{3}P_{2}^{{}^{\circ}}$	114482.59	
5*d*′ ${\;}^{1}P_{1}^{{}^{\circ}}$	114897.43	
5*d*′ ${\;}^{3}P_{1}^{{}^{\circ}}$	118074.09	
6*s*″ ${\;}^{3}P_{0}^{{}^{\circ}}$	105200.97	
6s″ ${\;}^{3}P_{1}^{{}^{\circ}}$	106103.28	
6s″ ${\;}^{3}P_{2}^{{}^{\circ}}$	110458.93	
6s″ ${\;}^{1}P_{1}^{{}^{\circ}}$	111961.58	
5*d*″ ${\;}^{1}D_{2}^{{}^{\circ}}$	113367.99	
5*d*″ ${\;}^{3}D_{3}^{{}^{\circ}}$	113656.16	
5*d*″ ${\;}^{3}P_{2}^{{}^{\circ}}$	117935.41	
5*d*″ ${\;}^{3}D_{2}^{{}^{\circ}}$	119321.09	
5*d*″ ${\;}^{3}D_{1}^{{}^{\circ}}$	121075.75	1.443
5*d*″ ${\;}^{3}F_{3}^{{}^{\circ}}$	121195.57	1.17 L
5*d*″ ${\;}^{3}F_{2}^{{}^{\circ}}$	122778.31	
5*d*″ ${\;}^{1}F_{3}^{{}^{\circ}}$	123580.02	
5*d*″ ${\;}^{3}P_{0}^{{}^{\circ}}$	125040.?	
5*d*″ ${\;}^{1}P_{1}^{{}^{\circ}}$	127890.23	0.89 L
5*d*″ ${\;}^{3}P_{1}^{{}^{\circ}}$	132734.92	
7s ${\;}^{5}S_{2}^{{}^{\circ}}$	118607.56	1.894
7*s* ${\;}^{3}S_{1}^{{}^{\circ}}$	119888.77	
6*d* ${\;}^{5}D_{0}^{{}^{\circ}}$	121701.69	
6*d* ${\;}^{5}D_{1}^{{}^{\circ}}$	122243.58	1.466
6*d* ${\;}^{5}D_{2}^{{}^{\circ}}$	121818.65	1.453
6*d* ${\;}^{5}D_{3}^{{}^{\circ}}$	121778.34	1.424
6*d* ${\;}^{5}D_{4}^{{}^{\circ}}$	121826.09	1.476
6*d* ${\;}^{3}D_{3}^{{}^{\circ}}$	125288.29	1.28
6*d* ${\;}^{3}D_{2}^{{}^{\circ}}$	125247.50	1.095
6*d* ${\;}^{3}D_{1}^{{}^{\circ}}$	125339.01	0.674
7*s*′ ${\;}^{3}D_{1}^{{}^{\circ}}$	130615.21	
7*s*′ ${\;}^{3}D_{2}^{{}^{\circ}}$	130617.58	
7*s*′ ${\;}^{3}D_{3}^{{}^{\circ}}$	133550.54	1.23 L
8*s* ${\;}^{5}S_{2}^{{}^{\circ}}$	132837.24	
8*s* ${\;}^{3}S_{1}^{{}^{\circ}}$	133297.08	
6*d*′ $1_{2}^{{}^{\circ}}$	133299.80	
6*d*′ $2_{3}^{{}^{\circ}}$	133955.51	1.16
6*d*′ $3_{3}^{{}^{\circ}}$	134023.88	0.81
6*d*′ $4_{0}^{{}^{\circ}}$	134235.94	
6*d*′ $5_{1}^{{}^{\circ}}$	134913.96	
6*d*′ $6_{1,2}^{{}^{\circ}}$	136443.37	
6*d*′ $7_{1,2}^{{}^{\circ}}$	136561.79	
6*d*′ $8_{4}^{{}^{\circ}}$	136733.42	1.21
6*d*′ $9_{3}^{{}^{\circ}}$	137505.08	1.24 L
6*d*′ $10_{2}^{{}^{\circ}}$	138316.49	1.27 L
6*d*′ $11_{0}^{{}^{\circ}}$	138364.08	
6*d*′ $12_{1}^{{}^{\circ}}$	138977.84	
6*d*′ $13_{1,2}^{{}^{\circ}}$	139551.74	1.09 L
6*d*′ $14_{3}^{{}^{\circ}}$	139634.07	1.04 L
6*d*′ $15_{1}^{{}^{\circ}}$	140500.35	
7*s*′ ${\;}^{1}D_{2}^{{}^{\circ}}$	134037.06	1.02 L
7*d* ${\;}^{5}D_{0}^{{}^{\circ}}$	134501.34	
7*d* ${\;}^{5}D_{1}^{{}^{\circ}}$	134546.93	
7*d* ${\;}^{5}D_{2}^{{}^{\circ}}$	134369.52	
7*d* ${\;}^{5}D_{3}^{{}^{\circ}}$	134441.71	
7*d* ${\;}^{5}D_{4}^{{}^{\circ}}$	134502.39	
7*d* ${\;}^{3}D_{3}^{{}^{\circ}}$	135584.94	
7*d* ${\;}^{3}D_{2}^{{}^{\circ}}$	134960.14	
7*d* ${\;}^{3}D_{1}^{{}^{\circ}}$	136187.03	
5*g* ${\;}^{5}G_{6}^{{}^{\circ}}$	136623.34	
5*g* ${\;}^{5}G_{5}^{{}^{\circ}}$	136573.00	
5*g* ${\;}^{5}G_{4}^{{}^{\circ}}$	136580.12	
5*g* ${\;}^{5}G_{3}^{{}^{\circ}}$	136613.38	
5*g* ${\;}^{5}G_{2}^{{}^{\circ}}$	136632.59	
5*g* ${\;}^{3}G_{5}^{{}^{\circ}}$	136623.74	
5*g* ${\;}^{3}G_{4}^{{}^{\circ}}$	136595.35	
5*g* ${\;}^{3}G_{3}^{{}^{\circ}}$	136633.77	
9*s* ${\;}^{5}S_{2}^{{}^{\circ}}$	139957.94	
9*s* ${\;}^{3}S_{1}^{{}^{\circ}}$	140125.66	
8*d* ${\;}^{5}D_{0}^{{}^{\circ}}$	140863.42	
8*d* ${\;}^{5}D_{1}^{{}^{\circ}}$	140887.40	
8*d* ${\;}^{5}D_{2}^{{}^{\circ}}$	140870.64	
8*d* ${\;}^{5}D_{3}^{{}^{\circ}}$	140879.23	
8*d* ${\;}^{5}D_{4}^{{}^{\circ}}$	140889.98	
8*d* ${\;}^{3}D_{1}^{{}^{\circ}}$	141520.48	
8*d* ${\;}^{3}D_{2}^{{}^{\circ}}$	141463.16	
8*d* ${\;}^{3}D_{3}^{{}^{\circ}}$	141634.22	
6*g* ${\;}^{5}G_{6}^{{}^{\circ}}$	142028.75	
6*g* ${\;}^{5}G_{5}^{{}^{\circ}}$	142018.43	
6*g* ${\;}^{5}G_{4}^{{}^{\circ}}$	142018.60	
6*g* ${\;}^{5}G_{3}^{{}^{\circ}}$	142022.92	
6*g* ${\;}^{5}G_{2}^{{}^{\circ}}$	142033.75	
6*g* ${\;}^{3}G_{5}^{{}^{\circ}}$	142030.64	
6*g* ${\;}^{3}G_{4}^{{}^{\circ}}$	142023.04	
6*g* ${\;}^{3}G_{3}^{{}^{\circ}}$	142035.14	
7*s*″ ${\;}^{3}P_{0}^{{}^{\circ}}$	143067.20	
7*s*″ ${\;}^{3}P_{1}^{{}^{\circ}}$	143246.49	
10*s* ${\;}^{5}S_{2}^{{}^{\circ}}$	144023.57	
10*s* ${\;}^{3}S_{1}^{{}^{\circ}}$	144182.29	
9*d* ${\;}^{5}D_{3}^{{}^{\circ}}$	144587.78	
9*d* ${\;}^{5}D_{4}^{{}^{\circ}}$	144591.35 ?	
9*d* ${\;}^{3}D_{2}^{{}^{\circ}}$	144832.02	
9*d* ${\;}^{3}D_{3}^{{}^{\circ}}$	144964.91	
8*s*′ ${\;}^{3}D_{1}^{{}^{\circ}}$	144641.34	
8*s*′ ${\;}^{3}D_{2}^{{}^{\circ}}$	144677.46	
7*g* ${\;}^{5}G_{6}^{{}^{\circ}}$	145289.63	
7*g* ${\;}^{5}G_{5}^{{}^{\circ}}$	145282.77	
7*g* ${\;}^{5}G_{4}^{{}^{\circ}}$	145283.23	
7*g* ${\;}^{5}G_{3}^{{}^{\circ}}$	145285.87	
7*g* ${\;}^{5}G_{2}^{{}^{\circ}}$	145292.52	
7*g* ${\;}^{3}G_{5}^{{}^{\circ}}$	145291.03	
7*g* ${\;}^{3}G_{4}^{{}^{\circ}}$	145285.17	
7*g* ${\;}^{3}G_{3}^{{}^{\circ}}$	145293.69	
$\begin{array}{cc} \begin{array}{r} 6d^{\prime \prime }\\ 7d^{\prime } \end{array} & \{\begin{array}{l} 20_{1,2}^{\circ }\\ 21_{1}^{\circ }\\ 24_{1,2}^{\circ }\\ 25_{1,2}^{\circ }\\ 26_{3}^{\circ }\\ 29_{1}^{\circ } \end{array} \end{array}$	145837.74	
	146291.96	
	146481.11	
	146784.94	
	146850.17	
	147020.40	
11*s* ${\;}^{5}S_{2}^{{}^{\circ}}$	146577.46	
11*s* ${\;}^{3}S_{1}^{{}^{\circ}}$	146589.32	
10*d* ${\;}^{3}D_{2}^{{}^{\circ}}$ ?	147141.92	
10*d* ${\;}^{3}D_{3}^{{}^{\circ}}$	147307.25	
$\begin{array}{ll} \begin{array}{r} 6d^{\prime \prime }\\ 7d^{\prime }\\ 7s^{\prime \prime }\\ 8s^{\prime }\\ 5g^{\prime } \end{array} & \{\begin{array}{l} 30_{2}^{{}^{\circ}}\\ 31_{0}^{{}^{\circ}}\\ 32_{1}^{{}^{\circ}}\\ 33_{3}^{{}^{\circ}}\\ 33.5_{1}^{{}^{\circ}}\\ 34_{2}^{{}^{\circ}}\\ 35_{2,3}^{{}^{\circ}}\\ 36_{2}^{{}^{\circ}}\\ 37_{2}^{{}^{\circ}}\\ 38_{1}^{{}^{\circ}}\\ 39_{4}^{{}^{\circ}}\\ 40_{3}^{{}^{\circ}} \end{array} \end{array}$	147242.50	
	147356.42	
	147792.67	
	147824.13	
	147863.97	
	147987.56	
	148079.42	
	148307.35	
	148656.42	
	148898.91	
	149266.12	
	149481.13	
8*g* ${\;}^{5}G_{6}^{{}^{\circ}}$	147405.72	
8*g* ${\;}^{5}G_{5}^{{}^{\circ}}$	147400.79	
8*g* ${\;}^{5}G_{4}^{{}^{\circ}}$	147401.38	
8*g* ${\;}^{5}G_{3}^{{}^{\circ}}$	147403.79	
8*g* ${\;}^{5}G_{2}^{{}^{\circ}}$	147408.13	
8*g* ${\;}^{3}G_{5}^{{}^{\circ}}$	147406.74	
8*g* ${\;}^{3}G_{4}^{{}^{\circ}}$	147404.11	
8*g* ${\;}^{3}G_{3}^{{}^{\circ}}$	147409.54	
12*s* ${\;}^{3}S_{1}^{{}^{\circ}}$	148354.?	
11*d* ${\;}^{3}D_{3}^{{}^{\circ}}$	148700.85	
9*g* ${\;}^{5}G_{6}^{{}^{\circ}}$	148856.10	
9*g* ${\;}^{5}G_{5}^{{}^{\circ}}$	148852.11	
9*g* ${\;}^{5}G_{4}^{{}^{\circ}}$	148853.15 ?	
9*g* ${\;}^{5}G_{3}^{{}^{\circ}}$	148854.55	
9*g* ${\;}^{5}G_{2}^{{}^{\circ}}$	148857.98	
9*g* ${\;}^{5}G_{5}^{{}^{\circ}}$	148856.65	
9*g* ${\;}^{5}G_{4}^{{}^{\circ}}$	148854.34	
9*g* ${\;}^{5}G_{3}^{{}^{\circ}}$	148858.89 ?	
12*d* ${\;}^{5}G_{3}^{{}^{\circ}}$	149644.47	
10*g* ${\;}^{5}G_{6}^{{}^{\circ}}$	149892.90	
$\begin{array}{cc} \begin{array}{l} 6d^{\prime \prime }\\ 7d^{\prime }\\ 5g^{\prime } \end{array} & \{\begin{array}{l} 41_{1,2,3}^{\circ }\\ 42_{2}^{\circ }\\ 43_{1,2}^{\circ }\\ 44_{2,3}^{\circ }\\ 45_{1,2}^{\circ }\\ 46_{2}^{\circ }\\ 47_{1,2}^{\circ }\\ 48_{1,2}^{\circ }\\ 49_{1,2}^{\circ }\\ 50_{3,4}^{\circ }\\ 51_{1,2,3}^{\circ } \end{array} \end{array}$	149866.64 ?	
	150002.97	
	150219.03	
	150324.10	
	150345.98	
	150366.61	
	150486.29	
	150594.	
	151744.	
	151917.75	
	152199.	
13*d* ${\;}^{3}D_{3}^{{}^{\circ}}$	150608.?	
11*g* ${\;}^{5}G_{6}^{{}^{\circ}}$	150659.78	
14*d* ${\;}^{3}D_{3}^{{}^{\circ}}$	151192.?	
12*g* ${\;}^{5}G_{6}^{{}^{\circ}}$	151242.53	

a The letter “L” following a *g_J_* value indicates that the value is taken from Lacroute [3].
